# Assessment of Studies of Quality Improvement Strategies to Enhance Outcomes in Patients With Cardiovascular Disease

**DOI:** 10.1001/jamanetworkopen.2021.13375

**Published:** 2021-06-14

**Authors:** Kavita Singh, Vidit Singh Bawa, Nikhil Srinivasapura Venkateshmurthy, Mareesha Gandral, Shuchita Sharma, Sugandha Lodhi, Q. Eileen Wafford, Shivani A. Patel, Nikhil Tandon, K. M. Venkat Narayan, Dorairaj Prabhakaran, Mark D. Huffman

**Affiliations:** 1 Centre for Chronic Conditions and Injuries, Public Health Foundation of India, Gurugram, India; 2 Centre for Chronic Disease Control, New Delhi, India; 3 Indian Institute of Public Health, Delhi, Public Health Foundation of India, Gurugram, India; 4 Feinberg School of Medicine, Northwestern University, Chicago, Illinois; 5 Rollins School of Public Health, Emory University, Atlanta, Georgia; 6 Department of Endocrinology, All India Institute of Medical Sciences, New Delhi, India; 7 The George Institute for Global Health, University of New South Wales, Kensington, Sydney, Australia

## Abstract

**Importance:**

Clinical care quality improvement (QI) strategies are critical to prevent and control cardiovascular disease (CVD). However, there is limited evidence regarding which components of the health system–, clinician-, and patient-based QI strategies contribute to their impact on CVD.

**Objectives:**

To identify, map, and organize evidence on the effectiveness and implementation of cardiovascular QI strategies that seek to improve outcomes in patients with CVD.

**Evidence Review:**

Eight electronic databases (MEDLINE, EMBASE, CINAHL, PsycINFO, the Cochrane Library, ProQuest, ClinicalTrials.gov, and the World Health Organization International Clinical Trials Registry Platform) were searched for studies published between January 1, 2009, and October 25, 2019. Eligible study designs included randomized trials and preintervention and postintervention evaluations. Descriptive findings of included studies were reported using several frameworks to map the intervention components stratified by target population, setting, outcomes, and overall results.

**Findings:**

From 8066 screened titles and abstracts, 456 unique studies with 150 148 unique patients (38.1% women and 61.9% men; mean [SD] age, 64.6 [7.1] years) were identified, including 427 randomized trials, 21 quasi-randomized studies, and 8 preintervention and postintervention studies. Of 336 studies from 45 countries that were classified, 255 (75.9%) were from high-income countries; 68 (20.2%), upper-middle–income countries; 13 (3.9%), lower-middle–income countries; and 0, low-income countries, with diverse clinical settings and target patient populations (post–myocardial infarction, stroke, heart failure). Patient support (311 studies), information communication technology (ICT) for health (78 studies), community support (18 studies), supervision (15 studies), and high-intensity training (14 studies) were the most commonly evaluated QI strategies. Other strategies were group problem-solving (7 studies), printed information (5 studies), strengthening infrastructure (4 studies), and financial incentives (3 studies). Patient support, ICT for health, training, and community support were strategies that had been evaluated the most for clinical end points and showed modest associations with several clinical outcomes. The other strategies did not have outcome-driven evaluations reported. Group problem-solving was associated with improved patient self-care and quality of life. Strengthening infrastructure was associated with improved treatment satisfaction. Printed information and financial incentives showed no meaningful effect.

**Conclusions and Relevance:**

This systematic review found that substantial variations exist in the types, effectiveness, and implementation of QI strategies for patients with CVD. A comprehensive map of QI strategies created by this study would be useful for researchers to identify where new knowledge is needed to improve cardiovascular outcomes. Outcome-driven evaluations and long-term studies are needed, particularly in low-income settings, to better understand the effects of QI strategies on prevention and control of CVD.

## Key Points

**Question:**

What are effective quality improvement strategies (patient-, clinician-, and health system–level) for improving outcomes in patients with cardiovascular disease (CVD), and how are these strategies best implemented?

**Findings:**

In this systematic scoping review, 456 studies were identified from 45 countries involving 150 148 patients that used 186 unique interventions to improve outcomes in patients with CVD. Strategies of patient support, information communication technology for health, and training were evaluated the most often for clinical end points and showed modest associations with several clinical outcomes.

**Meaning:**

These findings may inform decision makers and clinicians on the selection of quality improvement strategies to improve CVD outcomes.

## Teaser

This systematic and scoping review assesses evidence on the effectiveness and implementation of quality improvement strategies to improve outcomes in patients with cardiovascular disease (CVD).

## Introduction

Globally, cardiovascular disease (CVD) causes approximately 18 million deaths each year, and approximately 35 million people experience nonfatal cardiovascular events annually.^1^ The presence of CVD increases the risk of recurrent myocardial infarction, stroke, heart failure, or death by 2% to 5% per year compared with the absence of CVD.^2^ Evidence from high-income countries suggests that quality improvement (QI) programs such as multidisciplinary disease management strategies are beneficial for reducing CVD mortality, as well as all-cause and CVD-specific hospital admissions among patients with heart failure.^3,4,5^ Furthermore, QI programs for CVD in low- and middle-income countries (LMICs) have demonstrated various degrees of success.^6,7,8,9^ However, limited evidence exists regarding which components of the health system–, clinician-, and patient-based QI strategies (delivered by whom and to which targets) contribute to their impact.

In 2018, Rowe et al^10^ published the Health Care Provider Performance Review (HCPPR), a systematic review of studies that evaluated the effectiveness of a wide array of QI strategies to improve clinician performance in LMICs. However, there is no comprehensive global review of QI interventions (health system–, clinician-, and patient-level) for chronic care of CVD that can be locally adaptable to the outpatient clinic level in resource-constrained LMIC settings. The goal of the present systematic scoping review was to identify, map, and organize the available evidence from studies that assessed the effectiveness and implementation of cardiovascular QI interventions that seek to improve outcomes in patients with CVD in outpatient settings. Furthermore, we sought to report results that inform decisions on whether to use QI strategies and how best to implement them, as well as identify knowledge gaps in cardiovascular QI strategies for future research.

## Methods

We conducted a systematic scoping review in accordance with the methods outlined by Arksey and O’Malley^11^ and the Preferred Reporting Items for Systematic Review and Meta-analysis extension for Scoping Reviews (PRISMA-ScR) checklist.^12^ A protocol for the review was registered online.^13^ This study was reviewed and approved by the institutional ethics committee of the Public Health Foundation of India, Gurugram.

### Eligibility Criteria

We used the Population, Intervention, Comparator, Outcomes, and Study design elements to define eligibility criteria.^14,15^ For population, we included studies with adults (aged ≥18 years) who had established CVD and were enrolled in the study from outpatient clinics or patients who were recruited at the time of hospital discharge but were followed up longitudinally in outpatient settings. For intervention, we included studies that evaluated the effect of any QI intervention (health system–, clinician-, or patient-level) aimed at improving outcomes for patients with CVD at the outpatient clinic level. For comparators, we considered any standard or usual CVD care as the control group. For outcomes, we included studies that evaluated any of our primary outcomes of (1) changes in cardiovascular risk factors (blood pressure and total cholesterol level) and (2) major adverse cardiovascular events (MACEs). Our secondary outcomes included medication adherence, tobacco cessation, physical activity level, health-related quality of life, cost-effectiveness, hospital readmission rates, all-cause deaths, and treatment satisfaction. In addition, implementation outcomes included *acceptability* (willingness of patients/clinicians to receive/provide service), *fidelity* (degree to which the intervention was delivered as intended), and *feasibility* (in terms of capital intensity and technical complexity required to deliver a QI strategy). For study design, we included randomized clinical trials (RCTs), cluster RCTs, quasi-randomized studies, and preintervention and postintervention evaluations. Exclusion criteria included cohort, cross-sectional, and case-control studies; case reports; and opinion-driven reports (eg, editorials and reviews).

### Search Strategy

The search strategy was developed in collaboration with an information specialist (Q.E.W.) who incorporated keywords and controlled vocabulary terms for CVD, outpatient care, sources of quality improvement (health system, clinician, or patient), cardiovascular risk factors, cardiovascular events, and medication adherence. We adapted the search strategy and applied it to MEDLINE (Ovid), EMBASE (Elsevier), CINAHL with Full Text (EBSCO), PsycINFO (EBSCO), the Cochrane Library (Wiley), and ProQuest Dissertations & Theses Global. We incorporated the Cochrane Highly Sensitive Search Strategy to identify RCTs.^16^ We searched ClinicalTrials.gov and the World Health Organization International Clinical Trials Registry Platform for relevant studies. We performed the search without restrictions to language. We limited the search to studies published since January 1, 2009, to capture recent advances in health technology and therapeutics that would make contextualization and adaptation of QI interventions more relevant and applicable to current health care settings. We searched the databases on October 25, 2019. The detailed search strategy for each of the selected databases, including MeSH terms and key words, is provided in eMethods in the Supplement.

### Data Collection

All abstracts were reviewed for eligibility by a minimum of 2 reviewers (K.S., V.S.B., and/or N.S.V.) using an online software (Covidence [Cochrane Community]).^17^ Two independent reviewers (K.S. and V.S.B.) then reviewed the full-text articles to assess eligibility. Disagreements were resolved by consulting another author (M.D.H.) to achieve consensus. A standardized data collection tool was used by 5 authors (K.S., V.S.B., M.G., S.S., and S.L.) to extract relevant data to align with key domains captured in the HCPPR, such as study characteristics, target population (ie, health conditions), geography and type of health care setting, QI intervention, implementation strategy, duration of intervention, and outcome categories. Relevant extracted data were entered into an Excel spreadsheet, and graphs were made using Excel, version 2016 (Microsoft Corporation).

### Statistical Analysis

We reported descriptive tables to provide an overview of the included study characteristics. We reported studies excluded at the full-text review stage and the reasons for exclusion in the PRISMA flowchart. We characterized QI intervention components according to the HCPPR^10^ and the context using the 6 elements of the chronic care model^18^ because they provide a comprehensive approach to map the intervention components and context from the health system, clinician, and patient perspectives. Furthermore, we mapped the implementation strategies using the ERIC (Expert Recommendations for Implementing Change) study recommendations^19^ (eTable 1 in the Supplement). To map QI interventions and implementation strategies, we extracted information on (1) QI intervention and components, (2) predominant implementation strategy, (3) implementers and recipients (target population) of the intervention, and (4) frequency and duration of the intervention. The narrative analysis involved summarizing findings for each outcome using the HCPPR framework without meta-analysis given the purpose of this review and the expected heterogeneity.

## Results

### Database Search and Screening Results

**Figure 1.**
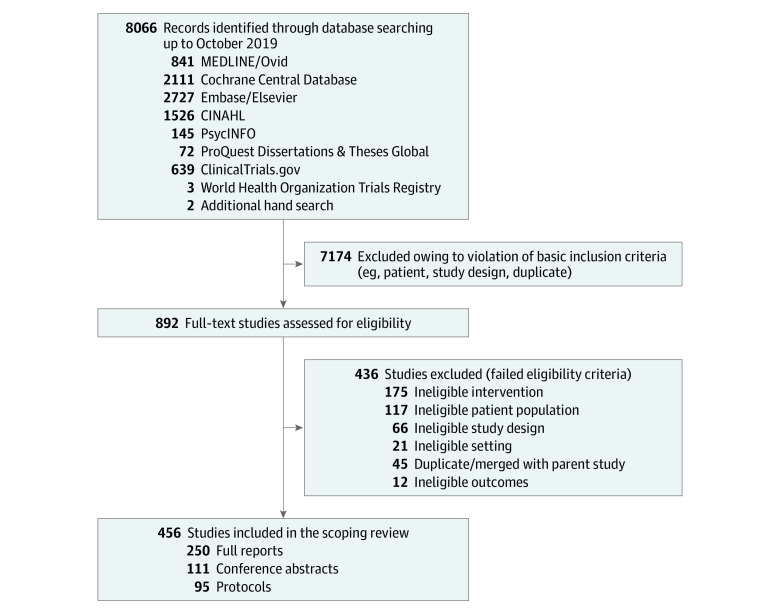
Summary of Scoping Review Search of the Literature

The literature search identified 8066 publications, which were reduced to 892 after screening of the abstracts. Of these, 456 unique studies met the eligibility criteria, including 250 full reports, 111 conference abstracts, and 95 study protocols. The summary of the search is provided in Figure 1. We noted a steady increase in the number of reports with data on cardiovascular QI strategies during the study period (2009-2019) (eFigure 1 in the Supplement). Overall, the included studies were from 45 countries involving 150 148 unique patients (38.1% women and 61.9% men; mean [SD] age, 64.6 [7.1] years) and evaluated 186 unique interventions.

### Description of Included Studies

The Table presents the descriptive characteristics of the 456 included studies, and a reference list is presented in the Supplement. Among the 456 studies, 427 were randomized trials, 21 were quasi-randomized studies, and 8 were preintervention and postintervention studies. The most common CVD conditions (target population studied) were heart failure (173 studies [37.9%]), stroke (126 studies [27.6%]), post–myocardial infarction (64 studies [14.0%]), and stable coronary artery disease (46 studies [10.1%]). Intervention components were mapped using several frameworks. Roughly two-thirds of the studies (311 [68.2%]) used patient support as the main QI intervention, followed by information communication technology (ICT) for health (78 [17.1%]), community support (18 [3.9%]), supervision (15 [3.3%]), and high-intensity training (14 [3.1%]). Few studies used group problem-solving (7 [1.5%]), printed information (5 [1.1%]), strengthening infrastructure (4 [0.9%]), or clinician-directed financial incentives (3 [0.7%]). We found that most strategies used a combination of the 6 elements of the chronic care model (200 studies [39.2%]), followed by decision support (113 studies [21.9%]), delivery system design (99 studies [19.2%]), and self-management support (68 studies [13.2%]). Even fewer studies reported use of community resources (4 [0.8%]) or organizational support (2 [0.4%]).

**Table.** Descriptive Characteristics of Patient Populations of the Included Studies

Characteristic	No. (%) of studies (N = 456)
Heart failure	173 (37.9)
Stroke	126 (27.6)
Post–myocardial infarction	64 (14.0)
Stable coronary artery disease	46 (10.1)
CVD	40 (8.8)
CVD plus comorbid diabetes	5 (1.1)
Peripheral arterial disease	2 (0.4)
Intervention types (HCPPR framework)	
Patient support	311 (68.2)
ICT for health	78 (17.1)
Community support	18 (3.9)
Supervision	15 (3.3)
High-intensity training	14 (3.1)
Group problem-solving	7 (1.5)
Printed information	5 (1.1)
Strengthening infrastructure	4 (0.9)
Financial incentives	3 (0.7)
Other management techniques	1 (0.2)
Comparator	
Usual care	396 (86.8)
Active comparator	33 (7.2)
Not reported	27 (5.9)
Study design	
RCTs	413 (90.6)
Quasi-randomized studies	21 (4.6)
Cluster randomized trials	14 (3.1)
Preintervention and postintervention evaluations	8 (1.8)
Clinical setting	
Tertiary care hospital	221 (48.5)
Hospital- plus home-based care	130 (28.5)
Primary care hospital	19 (4.2)
Secondary care hospital	8 (1.8)
Community level hospital	8 (1.8)
Not reported	70 (15.4)
Moment of intervention delivery	
Rehabilitation^a^	228 (50.0)
Discharge period	123 (27.0)
Chronic phase	82 (18.0)
Combination	19 (4.2)
Before and after discharge	1 (0.2)
Not reported	3 (0.7)
Intervention delivery mode	
NPHW	152 (33.3)
Technology	41 (9.0)
Telephone calls	39 (8.6)
Text messages	9 (2.0)
Combination^b^	170 (37.3)
Not reported	45 (9.9)
Intervention frequency	
Weekly to biweekly	103 (22.6)
Monthly to bimonthly	37 (8.1)
Every 3 mo	24 (5.3)
Irregular	141 (30.9)
Single episode	6 (1.3)
Not reported	145 (31.8)
Intervention duration, y	
<1	258 (56.6)
1-2	123 (27.0)
3-5	11 (2.4)
>5	1 (0.2)
Not reported	63 (13.8)
Location (World Bank region)	
Europe and Central Asia	110 (24.1)
East Asia and the Pacific	97 (21.3)
North America	93 (20.4)
Middle East and North Africa	12 (2.6)
South Asia	10 (2.2)
Latin America and the Caribbean	10 (2.2)
Sub-Saharan Africa	4 (0.9)
Not reported	120 (26.3)
Income group^c^	
High-income countries	255 (75.9)
Upper-middle–income countries	68 (20.2)
Lower-middle–income countries	13 (3.9)
Low-income countries	0
Funding source	
Government	146 (32.0)
Private	25 (5.5)
Nonprofit organization	23 (5.0)
No funding	19 (4.2)
Not reported	243 (53.3)

Abbreviations: CVD, cardiovascular disease; HCPPR, Health Care Provider Performance Review; ICT, information communication technology; NPHW, nonphysician health worker; RCT, randomized clinical trial.

^a^ Indicates cardiac rehabilitation (eg, a customized outpatient program of exercise and education).

^b^ Indicates more than 1 intervention delivery mode (eg, NPHW, technology, and/or telephone calls were combined).

^c^ Indicates World Bank classification for 45 countries in 336 studies that were classified.

Most studies evaluated QI strategies vs usual care/status quo (396 [86.8%]), and fewer studies used an active comparator (33 [7.2%]). Individual-level RCTs (413 [90.6%]) were the most commonly used study design. Nearly half of included studies (221 [48.5%]) were conducted in tertiary care hospitals, and more than one-quarter of studies (130 [28.5%]) involved both hospital- and home-based care. Few studies involved community-level (8 [1.8%]), primary care (19 [4.2%]), or secondary care (8 [1.8%]) hospitals. The most common moments of intervention delivery were during the rehabilitation phase (228 studies [50.0%]), at discharge (123 studies [27.0%]), during the chronic phase (82 studies [18.0%]), and a combination of discharge, rehabilitation, and outpatient settings (19 studies [4.2%]). Multifactorial strategies (eg, a combination of task-sharing plus text messages plus technology) (160 studies [35.1%]) and nonphysician health workers (NPHWs) alone (152 studies [33.3%]) constituted the most commonly evaluated intervention delivery modes. Intervention frequency varied from weekly to biweekly (103 studies [22.6%]), monthly to bimonthly (37 studies [8.1%]), every 3 months (24 studies [5.3%]), and irregular (141 studies [30.9%]). More than one-half of studies reported an intervention duration of less than 1 year (258 [56.6%]), and only 12 studies (2.6%) reported a duration that was longer than 3 years. Notably, no studies from low-income economies were included. Of 336 studies in which economies were classified, 255 (75.9%) were from high-income countries, 68 (20.2%) were from upper-middle–income countries, and 13 (3.9%) were from lower-middle–income countries. The number of studies by location is provided in the Table and in eFigure 2 in the Supplement.

eFigures 3 to 7 in the Supplement present a comparative description of intervention types vs implementation strategies, patient population, clinical setting, study location, and overall study results. eTable 2 in the Supplement reports a descriptive summary of study outcomes, follow-up duration, sample size, and overall results. The mean follow-up duration reported was relatively short (9.1 [SD, 9.7] months). Sample sizes ranged from 10 to 21 752 participants. Nearly half of the studies reported positive/significant results (227 [49.8%]) for primary outcomes, 45 (9.9%) had mixed results, and 76 (16.7%) had nonsignificant/neutral or negative results. Fewer than one-fifth of studies (84 [18.4%]) reported economic outcomes.

**Figure 2.**
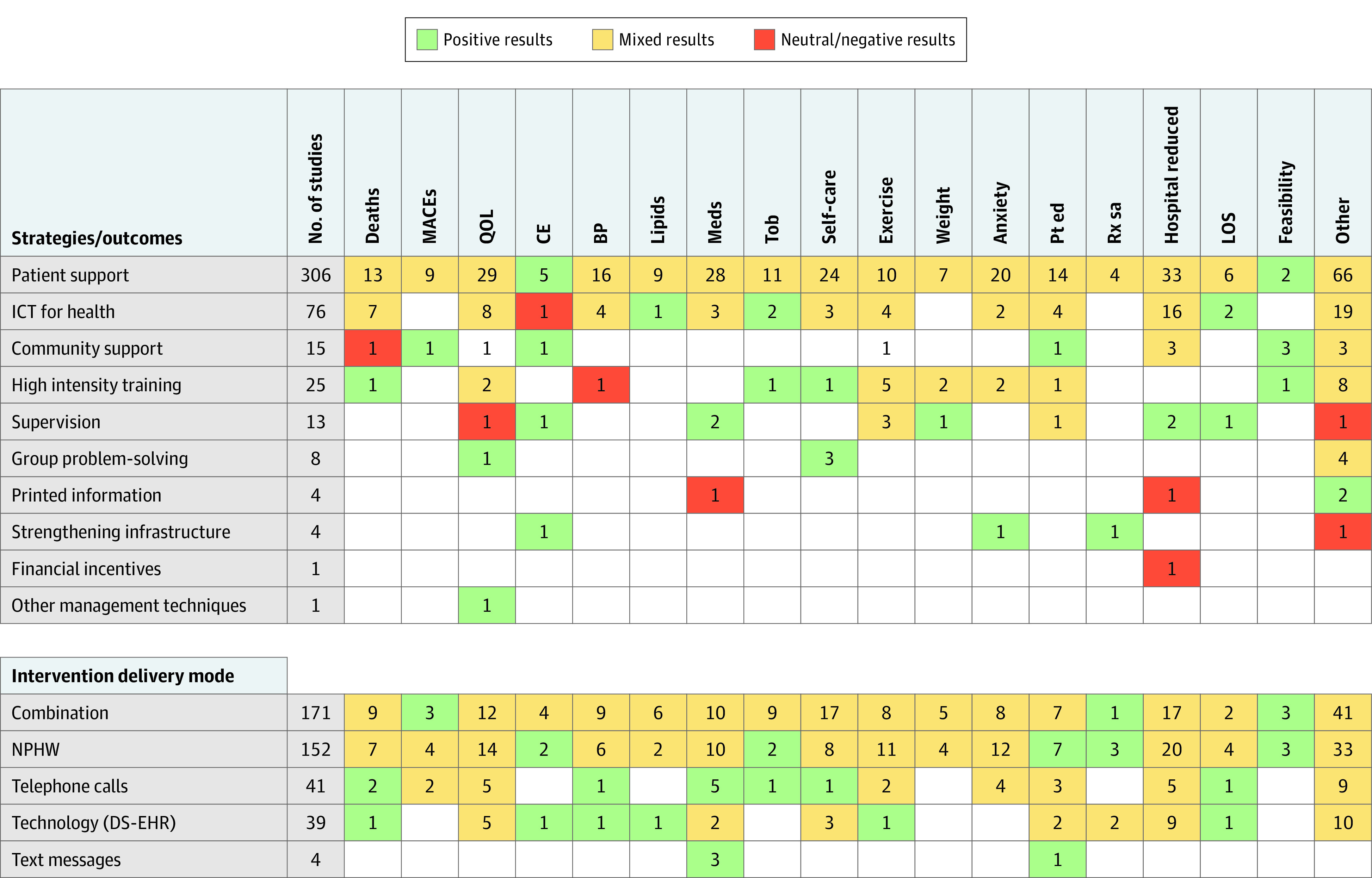
Quality Improvement Strategies, Primary Outcomes, and Overall Results BP indicates blood pressure changes; CE, cost-effectiveness; DS-EHR, decision-support electronic health records; ICT, information communication technology; Lipids, control of lipid levels; LOS, length of stay; MACEs, major adverse cardiovascular events; Meds, medication adherence; NPHW, nonphysician health worker; Pt ed, patient education; QOL, quality of life; Rx sa, treatment satisfaction; and Tob, tobacco cessation.

Figure 2 presents the overall study results for selected primary outcomes stratified by QI strategies. Overall, 422 studies (92.5%) investigated patient support, ICT for health, community support, and training measures, 32 of which evaluated clinical outcomes such as mortality or MACEs, with findings ranging from positive to negative/neutral. One study involving community support showed improvements in MACE outcomes for participants in the intervention group. Few studies evaluated changes in blood pressure (21 [4.6%]) or blood cholesterol level (10 [2.2%]) as primary outcomes, mostly using patient support or ICT for health, and all showed mixed results except for 1 study that was associated with a significant reduction in total cholesterol levels among participants allocated to an ICT for health strategy. Overall results for other outcomes such as hospital readmission, quality of life, medication adherence, and self-care behaviors varied substantially across different QI strategies. Eight studies investigated a group problem-solving measure, 3 of which demonstrated improvement in patient self-care, and 1 study showed improvements in quality of life. One study each found strengthening infrastructure to be cost effective, associated with a lower rate of anxiety/depression, and associated with improved treatment satisfaction. All 6 studies that assessed the feasibility of implementing patient support (n = 2), community support (n = 3), and training (n = 1) had positive findings.

In a subanalysis of overall study results for primary outcomes stratified by intervention delivery mode, we found that the use of NPHWs alone and a combination strategy (NPHW, technology, and telephone calls or text messages) mostly led to mixed results. Notably, the NPHW-based strategy was cost-effective (2 studies) and was associated with improved patient education (7 studies), treatment satisfaction (3 studies), and tobacco cessation (2 studies), whereas the combination strategy improved MACE outcomes (3 studies) and treatment satisfaction (1 study) and demonstrated intervention feasibility (3 studies). Quality improvement interventions delivered via telephone calls (eg, telehealth services) were associated with mortality (2 studies) and length of hospital stay (1 study) and improved medication adherence (5 studies), tobacco cessation (1 study), and self-care (1 study). Likewise, QI interventions that involved use of technology, such as decision-support electronic health records, were cost-effective and were associated with reduced deaths and improved control of blood pressure and blood cholesterol levels. Last, interventions delivered using text messages were associated with improved medication adherence and patient education (eFigure 7 in the Supplement).

## Discussion

We reviewed 456 studies, mostly RCTs, involving 150 148 unique patients and 186 unique interventions to map and organize the existing evidence on cardiovascular QI strategies in patients with CVD. To the best of our knowledge, this systematic scoping review is the most comprehensive global review of studies that have evaluated the effectiveness and implementation of cardiovascular QI strategies in patients with CVD. We found a large number of studies that had a robust study design (413 RCTs) and evaluated diverse QI strategies (186 interventions) to improve performance/outcomes for several cardiovascular conditions (eg, coronary artery disease, stroke, or heart failure), which were conducted in a wide variety of clinical settings and regions.

The goal of mapping and organizing results from this review was challenged by the methodological and contextual heterogeneity of the included studies, as well as the heterogeneity of the QI interventions and implementation strategies. Several strategies were tested by a single study, which limits generalizability, and studies generally had short follow-up duration (mean follow-up, 9.1 months), which reduces their relevance to programs that require strategies with sustained effect. However, we identified several intermingled or divergent findings regarding cardiovascular QI interventions for different outcome measures. Although patient support was found to be cost effective and feasible, it showed mixed results for clinical outcomes such as deaths, MACEs, medication adherence, and control of blood pressure and blood cholesterol levels. Information communication technology for health was associated with reducing blood cholesterol levels and length of hospital stays and increasing tobacco cessation, but was not cost-effective. One study investigating a community support measure showed an association with lower MACE outcomes. Group problem-solving, although studied exclusively in high-income countries, was associated with improved quality of life and patient self-care. Strengthening infrastructure was cost effective and improved treatment satisfaction. Both printed information and financial incentives were not associated with medication adherence and readmissions. We observed substantial variations in effectiveness within most strategies, including inconsistencies across primary study outcomes. These inconsistencies were likely due to differences in outcome definitions, measurement methods, and how the effect sizes were calculated. The variability of the effectiveness of QI strategies demonstrates the difficulty in estimating the effectiveness of any strategy in pooled analysis and suggests that it may be more important to monitor implementation and effectiveness within specific practice settings for a longer term. Also, most QI strategies (group problem-solving, supervision, financial incentives, health system strengthening) lack clinical outcome-based evaluations.

Our findings support several conclusions about the potential effectiveness and implementation of QI strategies among patients with CVD. The QI strategies such as patient support, community support, supervision, strengthening infrastructure, NPHWs, and technology-delivered strategies were all found to be cost-effective. Nevertheless, most patient support and ICT for health strategies had modest associations with several clinical outcomes. The feasibility of most QI strategies has been established across high-income countries of North America, Europe, and Asia. Furthermore, few studies tested strategies such financial incentives, strengthening infrastructure, or other management techniques.

Our results are consistent with previous systematic reviews^6,10^ evaluating QI interventions in patients who are at high risk for CVD. A 2016 systematic review of 49 studies focused on improving quality of cardiovascular care in LMICs found that QI strategies can be implemented successfully but emphasized longer-term follow-up to inform sustainability of interventions.^6^ The 2018 review by Rowe et al^10^ of 337 studies (118 QI strategies) sought to assess the effectiveness of clinician-level strategies in LMICs. The authors found that group problem-solving alone (28-37 percentage points of improvement), community support and clinician training (8-125 percentage points of improvement), and multifaceted strategies were associated with the largest improvements in clinician performance. Training and supervision had moderate associations (10-16 percentage points of improvement) with clinician performance. However, no benefits in clinician practices were found with only implementing a technology-based strategy (median effect size, 1.0 percentage points) or only providing printed information (1.4 percentage points of improvement). Notably, that review^10^ had important limitations (and high risk of bias) that threatened internal validity.

This review only includes studies that assessed the effectiveness and implementation of QI strategies for CVD. However, decisions about which strategies a health program should adopt in a given setting depend on many factors, such as intervention effectiveness, cost, feasibility, appropriateness, availability, resource requirements, technical complexities, and political and cultural acceptability. Future research should consider using standardized methods, stronger study designs, longer follow-up, and replication of research to have a better understanding of the influence of context on QI strategy effectiveness and implementation (Box). Furthermore, how health care teams and health systems can ensure continuity of patient support, community support, and ICT for health and the barriers and facilitators of scale-up in diverse settings should be studied. Policies to enable wider implementation and uptake of proven QI strategies (such as establishment of patient safety and quality councils to monitor implementation of QI programs and integration of NPHWs and clinical decision support systems in chronic care delivery models) are warranted to reduce deaths due to CVD and disability and to improve patients’ self-care behaviors or quality of life.

Box. **Quality Improvement Research Priorities to Improve Cardiovascular Health Care**

**Populations and Contexts**

- Establish quality improvement (QI) collaboratives and learning networks in resource-constrained settings, particularly within low-income countries.
- Expand QI implementation to include a broad array of cardiovascular diseases.
- Understand sex differences, including strategies to equitably provide quality cardiovascular care for women, as well as other historically disadvantaged groups.
- Promote cross-collaboration and multicounty studies evaluating similar QI strategies using standardized methods to better understand contextual implementation and effectiveness.

**Interventions and Implementation Strategies**

- Promote contextualization and implementation of community-based interventions, training, supervision, and group problem-solving measures in diverse clinical settings, including rural and underserved communities, given their effectiveness.
- Determine relative benefits of multicomponent QI interventions compared with simpler approaches.
- Promote integrated, team-based, people-centered care across tertiary, secondary, and primary care facilities due to low ratio of physicians to patients in low- and middle-income countries.
- Develop, implement, and evaluate QI innovations with minimal clinician burden.

**Clinical Outcome Measurement and Study Design**

- Create an integrated research framework to include detailed cardiovascular event adjudication for longer time horizons.
- Capture patient-centered outcomes and measures of experiential quality of cardiovascular disease care.
- Embed clinical research, including clinical trials, into routinely collected health record systems in low- and middle-income countries.

### Strengths and Limitations

One of the strengths of this systematic scoping review is its comprehensive search strategy in multiple databases. The breadth of the review is wider than the most recent systematic and narrative reviews^2,3,4,5,6,7,8,9,10^ conducted on this topic. The scoping review concept and methods allowed for a more conceptual investigation and mapping of cardiovascular QI interventions, implementation strategies, their components, context, target population, and a wide array of study outcomes reported by the researchers to determine the effectiveness and implementation of QI strategies for chronic care of CVD across different regions worldwide. The inclusion of different study designs (ie, RCTs and nonrandomized preintervention and postintervention controlled studies) enabled us to review different types of interventions used for managing CVD in outpatient settings. We incorporated studies with diverse cardiovascular conditions and clinical settings and attempted to organize and map evidence not only on intervention effectiveness, but also its implementation.

This scoping review has some limitations. First, many of the included studies had limitations in design and reporting, such as inadequate details of QI strategy and context (including how and why strategies were chosen), heterogeneity of QI strategy implementation methods, and difficulty in assessing fidelity of implementation and risk of bias because they were reported as conference abstracts with no published, full-length studies to provide more context regarding the intervention. Second, our analytical approach was designed to identify broad patterns across all studies using several frameworks. Our results could not reflect important nuances pertaining to multifaceted interventions such as the pooled/additive effect of combining more than 1 QI strategy. Future analyses would benefit from more specific classification (eg, separating out results from different combination strategies using patient support and ICT for health) and network meta-analyses. Third, we may have missed relatively new publications. Fourth, our review focused solely on chronic outpatient care interventions. A future scoping review should evaluate the quality of acute CVD care (eg, interventions targeted to reduce door-to-needle time, measurement of ejection fraction before discharge) that could have a considerable effect on CVD outcomes at population level.

## Conclusions

A comprehensive map of cardiovascular QI strategies created from this systematic scoping review may be useful for researchers to identify where new knowledge is needed to improve cardiovascular outcomes. Considering substantial variations in types, effectiveness, and implementation, future research should focus on high-quality, outcome-driven, and longer-term studies to understand the potential effect of QI strategies on cardiovascular health.

## Notes

Supplement. **eMethods.** Literature Search Strategy **eReferences.** List of Included Studies in Chronological Order (N = 456) **eFigure 1.** Number and Types of Studies by Publication Year **eFigure 2. **Number of Studies by Location **eFigure 3.** Comparative Description of Intervention Type and Implementation Strategies **eFigure 4.** Intervention Type and Patient Population **eFigure 5.** Intervention Type and Clinical Setting **eFigure 6.** Intervention Type and Study Location **eFigure 7. **Detailed Summary of Overall Study Results Using Matrix Framework for the Cardiovascular Quality Improvement Strategies and Primary Outcomes Evaluated in the Included Studies (N = 456) **eTable 1.** Domains and Frameworks Used to Map Intervention Components, Implementation Strategies, and Context **eTable 2.** Descriptive Summary of Study Outcomes, Follow-up Duration, Sample Size, and Overall Results in the Included Studies (N = 456)
