# Supplementary material for: Assessment of Studies of Quality Improvement Strategies to Enhance Outcomes in Patients With Cardiovascular Disease
Source: JAMA Netw Open. 2021 Jun 14;4(6):e2113375. doi: 10.1001/jamanetworkopen.2021.13375 (PMC8204210; doi:10.1001/jamanetworkopen.2021.13375)
Supplement: Supplement. — eMethods. Literature Search Strategy eReferences. List of Included Studies in Chronological Order (N = 456) eFigure 1. Number and Types of Studies by Publication Year eFigure 2. Number of Studies by Location eFigure 3. Comparative Description of Intervention Type and Implementation Strategies eFigure 4. Intervention Type and Patient Population eFigure 5. Intervention Type and Clinical Setting eFigure 6. Intervention Type and Study Location eFigure 7. Detailed Summary of Overall Study Results Using Matrix Framework for the Cardiovascular Quality Improvement Strategies and Primary Outcomes Evaluated in the Included Studies (N = 456) eTable 1. Domains and Frameworks Used to Map Intervention Components, Implementation Strategies, and Context eTable 2. Descriptive Summary of Study Outcomes, Follow-up Duration, Sample Size, and Overall Results in the Included Studies (N = 456) [file jamanetwopen-e2113375-s001.pdf]

Supplementary Online Content

Singh K, Bawa VS, Venkateshmurthy NS, et al. Assessment of studies of quality improvement strategies to enhance outcomes in patients with cardiovascular disease. *JAMA Netw Open*. 2021;4(6):e2113375. doi:10.1001/jamanetworkopen.2021.13375

eMethods. Literature Search Strategy

eReferences. List of Included Studies in Chronological Order (N = 456)

eFigure 1. Number and Types of Studies by Publication Year

eFigure 2. Number of Studies by Location

eFigure 3. Comparative Description of Intervention Type and Implementation Strategies

eFigure 4. Intervention Type and Patient Population

eFigure 5. Intervention Type and Clinical Setting

eFigure 6. Intervention Type and Study Location

eFigure 7. Detailed Summary of Overall Study Results Using Matrix Framework for the Cardiovascular Quality Improvement Strategies and Primary Outcomes Evaluated in the Included Studies (N = 456)

eTable 1. Domains and Frameworks Used to Map Intervention Components, Implementation Strategies, and Context

eTable 2. Descriptive Summary of Study Outcomes, Follow-up Duration, Sample Size, and Overall Results in the Included Studies (N = 456)

This supplementary material has been provided by the authors to give readers additional information about their work.

© 2021 Singh K et al. *JAMA Network Open*.

eMethods. Literature Search Strategy

Ovid MEDLINE(R) and Epub Ahead of Print, In-Process & Other Non-Indexed Citations, Daily and Versions(R) 1946 to October 24, 2019

Search Strategy:

#	Searches	Results
1	exp Cardiovascular Diseases/	2314179
2	exp Stroke/	126594
3	(acute coronary syndrome or angina pectoris or asystole* or cardiac arrest* or cardiac infarct* or cardiovascular disease* or cardiovascular outcome* or cardiovascular event* or cardiovascular risk* or cardiovascular stroke or cardiopulmonary arrest* or coronary artery disease* or coronary artery obstruction* or coronary artery thrombos* or coronary occlusion or coronary heart disease* or heart arrest* or heart attack* or heart failure or heart infarct* or high blood pressure or hypertension or ischaemic heart disease* or ischemic heart disease* or myocardial infarct* or myocardial failure or myocardium infarct* or myocardium failure or percutaneous coronary intervention* or peripheral arterial disease or stenocardia* or stroke* or subclinical atherosclerosis or subclinical cardiovascular).ti,ab.	1152578
4	or/1-3	2674261
5	exp Patient Discharge/	27998
6	exp Home Care Services/	46279
7	exp Outpatient Clinics, Hospital/	16883
8	exp Outpatients/	15098
9	(patient discharge* or post hospitalization or post hospitalisation or home care or homecare or outpatient* or "out patient").ti,ab.	188653
10	or/5-9	260519
11	"health care quality, access, and evaluation"/	0
12	exp quality assurance, health care/	318636
13	"quality of health care"/	70775
14	*clinical competence/	44216
15	"outcome and process assessment (health care)"/	26242
16	"outcome assessment (health care)"/	69700
17	"process assessment (health care)"/	4508
18	peer review, health care/	1420
19	exp program evaluation/	73203
20	quality improvement/	21918
21	quality indicators, health care/	14998
22	*Patient Satisfaction/	28392
23	Management Quality Circles/	1229
24	Employee Performance Appraisal/	4585
25	exp Reminder Systems/	3304
26	*Checklist/	2492
27	*Patient Education as Topic/	38151
28	*health education/	34757
29	exp Inservice Training/	28480
30	management audit/	2500
31	(checklist* or check list* or quality circle* or quality management or patient satisfaction or patient education or change management or continuing education or reminder system* or process assess*).ti,ab.	107741
32	(quality adj3 (indicat* or improv* or assurance* or assess* or evaluat* or feedback)).ti,ab.	259905
33	(practice* adj3 complian*).ti,ab.	1312
34	(guideline* adj3 (adherence or complian*)).ti,ab.	9071
35	(HCP adj3 (attitude* or documentation* or knowledge or satisfaction)).ti,ab.	110
36	(audit adj3 feedback).ti,ab.	1262
37	(benchmark* adj3 (assess* or performance or analysis or quality or evalulat*)).ti,ab.	3432
38	(train* adj (inservice or "in service" or staff)).ti,ab.	2166
39	or/11-38	956967
40	(randomized controlled trial or clinical trial).pt.	797133
41	trial.ti.	206914
42	(randomized or randomised or randomly or groups or "interrupted time series" or "before and after intervention study" or "before and after intervention studies" or controlled).ti,ab.	2917987

© 2021 Singh K et al. *JAMA Network Open*.

43	exp animals/ not humans.sh.	4636899
44	(40 or 41 or 42) not 43	2815401
45	4 and 10 and 39 and 44	1502
46	limit 45 to yr="2009 -Current"	841

Cochrane Library

ID Search Hits
#1 MeSH descriptor: [Cardiovascular Diseases] explode all trees 98574
#2 MeSH descriptor: [Stroke] explode all trees 8542
#3 (acute coronary syndrome or angina pectoris or asystole* or cardiac arrest* or cardiac infarct* or cardiovascular disease* or cardiovascular outcome* or cardiovascular event* or cardiovascular risk* or cardiovascular stroke or cardiopulmonary arrest* or coronary artery disease* or coronary artery obstruction* or coronary artery thrombos* or coronary occlusion or coronary heart disease* or heart arrest* or heart attack* or heart failure or heart infarct* or high blood pressure or hypertension or ischaemic heart disease* or ischemic heart disease* or myocardial infarct* or myocardial failure or myocardium infarct* or myocardium failure or percutaneous coronary intervention* or peripheral arterial disease or stenocardia* or stroke* or subclinical atherosclerosis or subclinical cardiovascular):ti,ab 186262
#4 #1 or #2 or #3 224286
#5 MeSH descriptor: [Patient Discharge] explode all trees 1385
#6 MeSH descriptor: [Home Care Services] explode all trees 2323
#7 MeSH descriptor: [Outpatient Clinics, Hospital] explode all trees 649
#8 MeSH descriptor: [Outpatients] explode all trees 1161
#9 (patient discharge* or post hospitalization or post hospitalisation or home care or homecare or outpatient* or "out patient"):ti,ab,kw 72616
#10 #5 OR #6 OR #7 OR #8 OR #9 72806
#11 MeSH descriptor: [Health Care Quality, Access, and Evaluation] this term only 0
#12 MeSH descriptor: [Quality Assurance, Health Care] explode all trees 3155
#13 MeSH descriptor: [Quality of Health Care] this term only 832
#14 MeSH descriptor: [Clinical Competence] this term only 3096
#15 MeSH descriptor: [Outcome and Process Assessment (Health Care)] this term only 2055
#16 MeSH descriptor: [Outcome Assessment (Health Care)] this term only 6920
#17 MeSH descriptor: [Process Assessment (Health Care)] this term only 206
#18 MeSH descriptor: [Peer Review, Health Care] this term only 34
#19 MeSH descriptor: [Program Evaluation] explode all trees 5895
#20 MeSH descriptor: [Quality Improvement] this term only 611
#21 MeSH descriptor: [Quality Indicators, Health Care] this term only 211
#22 MeSH descriptor: [Patient Satisfaction] this term only 10989
#23 MeSH descriptor: [Management Quality Circles] this term only 19
#24 MeSH descriptor: [Employee Performance Appraisal] this term only 40
#25 MeSH descriptor: [Reminder Systems] explode all trees 864
#26 MeSH descriptor: [Checklist] this term only 248
#27 MeSH descriptor: [Patient Education as Topic] this term only 8374
#28 MeSH descriptor: [Health Education] this term only 3721
#29 MeSH descriptor: [Inservice Training] explode all trees 784

© 2021 Singh K et al. *JAMA Network Open*.

Embase Session Results

No.	Query	Results
**#36**	**#35** AND [2009-2019]/py	**2,727**
**#35**	**#33** NOT **#34**	**3,676**
**#34**	('**animal**'/exp OR '**nonhuman**'/exp) NOT '**human**'/exp	**7,047,449**
**#33**	**#3** AND **#9** AND **#29** AND **#32**	**3,685**
**#32**	**#30** OR **#31**	**9,483,905**
**#31**	'**controlled clinical trial**'/de OR '**controlled study**'/de OR '**randomized controlled trial**'/de	**7,085,221**
**#30**	**randomized**:ti,ab OR **randomised**:ti,ab OR **randomly**:ti,ab OR **groups**:ti,ab OR **trial**:ti OR '**interrupted time series**':ti,ab OR '**before and after intervention study**':ti,ab OR '**before and after intervention studies**':ti,ab OR **controlled**:ti,ab	**4,146,791**
**#29**	**#10** OR **#11** OR **#12** OR **#13** OR **#14** OR **#15** OR **#16** OR **#17** OR **#18** OR **#19** OR **#20** OR **#21** OR **#22** OR **#23** OR **#24** OR **#25** OR **#26** OR **#27** OR **#28**	**829,196**
**#28**	(**train*** NEAR/1 (**inservice** OR '**in service**' OR **staff**)):ti,ab	**10,234**
**#27**	(**benchmark*** NEAR/3 (**assess*** OR **performance** OR **analysis** OR **quality** OR **evalulat***)):ti,ab	**4,446**
**#26**	(**audit** NEAR/3 **feedback**):ti,ab	**1,873**
**#25**	(**hcp** NEAR/3 (**attitude*** OR **documentation*** OR **knowledge** OR **satisfaction**)):ti,ab	**231**
		**16,067**

© 2021 Singh K et al. *JAMA Network Open*.

#24	(**guideline*** NEAR/3 (**adherence** OR **complian***)):ti,ab	**2,030**
#23	(**practice*** NEAR/3 **complian***):ti,ab	**373,388**
#22	(**quality** NEAR/3 (**indicat*** OR **improv*** OR **assurance*** OR **assess*** OR **evaluat*** OR **feedback**)):ti,ab	**153,292**
#21	**checklist***:ti,ab OR **'check list*'**:ti,ab OR **'quality circle*'**:ti,ab OR **'quality management'**:ti,ab OR **'patient satisfaction'**:ti,ab OR **'patient education'**:ti,ab OR **'change management'**:ti,ab OR **'continuing education'**:ti,ab OR **'reminder system*'**:ti,ab OR **'process assess*'**:ti,ab	**6,709**
#20	**'in service training'**/mj	**38,143**
#19	**'health education'**/mj	**28,637**
#18	**'patient education'**/mj	**3,562**
#17	**'checklist'**/mj	**1,095**
#16	**'reminder system'**/mj	**23,262**
#15	**'patient satisfaction'**/mj	**23,158**
#14	**'clinical competence'**/mj	**13,625**
#13	**'program evaluation'**/de	**4,112**
#12	**'performance measurement system'**/de	**1,824**
#11	**'quality control procedures'**/de	**235,524**
#10	**'health care quality'**/de	

© 2021 Singh K et al. *JAMA Network Open*.

**#9**<br>#**4** OR #**5** OR #**6** OR #**7** OR #**8**	**518,653**
**#8**<br>**'patient discharge*'**:ab,ti OR **'post hospitalization'**:ab,ti OR **'post hospitalisation'**:ab,ti OR **'home care'**:ab,ti OR **homecare**:ab,ti OR **outpatient***:ab,ti OR **'out patient'**:ab,ti	**303,629**
**#7**<br>**'outpatient'**/exp	**119,073**
**#6**<br>**'outpatient department'**/exp	**83,544**
**#5**<br>**'home care'**/exp	**73,238**
**#4**<br>**'hospital discharge'**/exp	**115,915**
**#3**<br>#**1** OR #**2**	**4,591,744**
**#2**<br>**'acute coronary syndrome'**:ab,ti OR **'angina pectorisasystole*'**:ab,ti OR **'cardiac arrest*'**:ab,ti OR **'cardiac infarct*'**:ab,ti OR **'cardiovascular disease*'**:ab,ti OR **'cardiovascular outcome*'**:ab,ti OR **'cardiovascular event*'**:ab,ti OR **'cardiovascular risk*'**:ab,ti OR **'cardiopulmonary arrest*'**:ab,ti OR **'coronary artery disease*'**:ab,ti OR **'coronary artery obstruction*'**:ab,ti OR **'coronary artery thrombos*'**:ab,ti OR **'coronary occlusion'**:ab,ti OR **'coronary heart disease*'**:ab,ti OR **'heart arrest*'**:ab,ti OR **'heart attack*'**:ab,ti OR **'heart failure'**:ab,ti OR **'heart infarct*'**:ab,ti OR **'high blood pressure'**:ab,ti OR **hypertension**:ab,ti OR **'ischaemic heart disease*'**:ab,ti OR **'ischemic heart disease*'**:ab,ti OR **'myocardial infarct*'**:ab,ti OR **'myocardial failure'**:ab,ti OR **'myocardium infarct*'**:ab,ti OR **'myocardium failure'**:ab,ti OR **'percutaneous coronary intervention*'**:ab,ti OR **'peripheral arterial disease'**:ab,ti OR **stenocardia***:ab,ti OR **stroke***:ab,ti OR **'subclinical atherosclerosis'**:ab,ti OR **'subclinical cardiovascular'**:ab,ti	**1,761,005**
**#1**<br>**'cardiovascular disease'**/exp	**4,401,003**

CINAHL Plus with Full Text

#	Query	Results
S29	S4 AND S9 AND S25 AND S28	1,526
S28	S26 OR S27	922,738
S27	TI trial	95,944
S26	randomized or randomised or randomly or groups or "interrupted time series" or "before and after intervention	904,986

© 2021 Singh K et al. *JAMA Network Open*.

	study" or "before and after intervention studies" or controlled	
S25	S10 OR S11 OR S12 OR S13 OR S14 OR S15 OR S16 OR S17 OR S18 OR S19 OR S20 OR S21 OR S22 OR S23 OR S24	922,200
S24	train* N (inservice OR "in service" OR staff)	1
S23	benchmark* N3 (assess* OR performance OR analysis OR quality OR evalulat*)	1,309
S22	audit N3 feedback	834
S21	HCP N3 (attitude* OR documentation* OR knowledge OR satisfaction)	192
S20	guideline* N3 (adherence OR complian*)	16,813
S19	practice* N3 complian*	927
S18	quality N3 (indicat* OR improv* OR assurance* OR assess* OR evaluat* OR feedback)	165,627
S17	checklist* OR "check list*" OR "quality circle*" OR "quality management" OR "patient satisfaction" OR "patient education" OR "change management" OR "continuing education" OR "reminder system*" OR "process assess*"	183,997
S16	(MM "Staff Development")	10,743
S15	(MM "Health Education")	14,462
S14	(MM "Patient Education")	23,741
S13	(MM "Reminder Systems")	1,365
S12	(MM "Patient Compliance")	12,742
S11	(MH "Employee Performance Appraisal+")	7,099
S10	(MH "Quality of Health Care+")	690,963
S9	S5 OR S6 OR S7 OR S8	145,040
S8	patient discharge* or post hospitalization or post hospitalisation or home care or homecare or outpatient* or "out patient"	138,818
S7	(MH "Outpatients")	43,300
S6	(MH "Outpatient Service")	7,986
S5	(MH "Patient Discharge+")	27,701
S4	S1 OR S2 OR S3	621,803
S3	acute coronary syndrome or angina pectorisasystole* or cardiac arrest* or cardiac infarct* or cardiovascular disease* or cardiovascular outcome* or cardiovascular event* or cardiovascular risk* or cardiovascular stroke or cardiopulmonary arrest* or coronary artery disease* or coronary artery obstruction* or coronary artery thrombos* or coronary occlusion or coronary heart disease* or heart arrest* or heart attack* or heart failure or heart infarct* or high blood pressure or hypertension or ischaemic heart disease* or ischemic heart disease* or myocardial infarct* or myocardial failure or myocardium infarct* or myocardium failure or percutaneous coronary intervention* or peripheral arterial disease or stenocardia* or stroke* or subclinical atherosclerosis or subclinical cardiovascular	407,578
S2	(MH "Stroke+")	62,118
S1	(MH "Cardiovascular Diseases+")	518,707

© 2021 Singh K et al. *JAMA Network Open*.

PsycINFO (EBSCOhost)

#	Query	Results
S29	S4 AND S9 AND S25 AND S28	145
S28	S26 OR S27	1,143,596
S27	TI trial	36,651
S26	randomized or randomised or randomly or groups or "interrupted time series" or "before and after intervention study" or "before and after intervention studies" or controlled	1,135,652
S25	S10 OR S11 OR S12 OR S13 OR S14 OR S15 OR S16 OR S17 OR S18 OR S19 OR S20 OR S21 OR S22 OR S23 OR S24	180,283
S24	train* N (inservice OR "in service" OR staff)	3,028
S23	benchmark* N3 (assess* OR performance OR analysis OR quality OR evalulat*)	1,086
S22	audit N3 feedback	174
S21	HCP N3 (attitude* OR documentation* OR knowledge OR satisfaction)	104
S20	guideline* N3 (adherence OR complian*)	3,191
S19	practice* N3 complian*	336
S18	quality N3 (indicat* OR improv* OR assurance* OR assess* OR evaluat* OR feedback)	58,459
S17	checklist* OR "check list*" OR "quality circle*" OR "quality management" OR "patient satisfaction" OR "patient education" OR "change management" OR "continuing education" OR "reminder system*" OR "process assess*"	115,300
S16	(MM "Staff Development")	1,139
S15	(MM "Health Education")	10,740
S14	(MM "Patient Education")	1,012
S13	(MM "Reminder Systems")	792
S12	(MM "Patient Compliance")	755
S11	(MH "Employee Performance Appraisal+")	147
S10	(MH "Quality of Health Care+")	976
S9	S5 OR S6 OR S7 OR S8	89,689
S8	patient discharge* or post hospitalization or post hospitalisation or home care or homecare or outpatient* or "out patient"	89,689
S7	(MH "Outpatients")	1
S6	(MH "Outpatient Service")	320
S5	(MH "Patient Discharge+")	245
S4	S1 OR S2 OR S3	82,696
S3	acute coronary syndrome or angina pectorisasystole* or cardiac arrest* or cardiac infarct* or cardiovascular disease* or cardiovascular outcome* or cardiovascular event* or cardiovascular risk* or cardiovascular stroke or cardiopulmonary arrest* or coronary artery disease* or coronary artery obstruction* or coronary artery thrombos* or coronary occlusion or coronary heart disease* or heart arrest* or heart attack* or heart failure or heart infarct* or high blood pressure or hypertension or ischaemic heart disease* or ischemic heart disease* or myocardial infarct* or myocardial failure or myocardium infarct* or myocardium failure or percutaneous coronary intervention* or peripheral arterial disease or stenocardia* or stroke* or subclinical atherosclerosis or subclinical cardiovascular	82,696

© 2021 Singh K et al. *JAMA Network Open*.

S2	(MH "Stroke+")	11
S1	(MH "Cardiovascular Diseases+")	67

ProQuest Dissertations & Theses Global

Select all	Set	Search	Results
Select item 10	**S10**	S1 AND S2 AND S3 AND S4Limits applied<br>Databases:<br>ProQuest Dissertations & Theses Global<br>Narrowed by:<br>Entered date: 2009 - 2019-10-31	**72**
Select item 9	**S9**	S1 AND S2 AND S3 AND S4<br>Databases:<br>ProQuest Dissertations & Theses Global	**129**
Select item 8	**S4**	noft(patient discharge* or post hospitalization or post hospitalisation or home care or homecare or outpatient* or "out patient")<br>Databases:<br>ProQuest Dissertations & Theses Global	**26,230**
Select item 7	**S3**	noft(train* NEAR/1 (inservice OR "in service" OR staff)) OR noft(benchmark* NEAR/3 (assess* OR performance OR analysis OR quality OR evalulat*)) OR noft(audit NEAR/3 feedback) OR noft(HCP NEAR/3 (attitude* OR documentation* OR knowledge OR satisfaction)) OR noft(guideline* NEAR/3 (adherence OR complian*)) OR noft(practice* NEAR/3 complian*) OR noft(quality NEAR/3 (indicat* OR improv* OR assurance* OR assess* OR evaluat* OR feedback)) OR noft(checklist* OR "check list*" OR "quality circle*" OR "quality management" OR "patient satisfaction" OR "patient education" OR "change management" OR "continuing education" OR "reminder system*" OR "process assess*")<br>Databases:<br>ProQuest Dissertations & Theses Global	**110,089**
Select item 2	**S2**	noft(randomized or randomised or randomly or groups or "interrupted time series" or "before and after intervention study" or "before and after intervention stud*" or controlled) OR ti(trial)<br>Databases:<br>ProQuest Dissertations & Theses Global	**744,339**
Select item 1	**S1**	noft(acute coronary syndrome OR angina pectorisasystole* OR cardiac arrest* OR cardiac infarct* OR cardiovascular disease* OR cardiovascular outcome* OR cardiovascular event* OR cardiovascular risk* OR cardiovascular stroke OR cardiopulmonary arrest* OR coronary artery disease* OR coronary artery obstruction* OR coronary artery thrombos* OR coronary occlusion OR coronary heart disease* OR heart arrest* OR	**2,004**

© 2021 Singh K et al. *JAMA Network Open*.

		heart attack* OR heart failure OR heart infarct* OR high blood pressure OR hypertension OR ischaemic heart disease* OR ischemic heart disease* OR myocardial infarct* OR myocardial failure OR myocardium infarct* OR myocardium failure OR percutaneous coronary intervention* OR peripheral arterial disease OR stenocardia* OR stroke* OR subclinical atherosclerosis OR subclinical cardiovascular) AND noft(patient discharge* OR post hospitalization OR post hospitalisation OR home care OR homecare OR outpatient* OR "out patient")<br>Databases:<br>ProQuest Dissertations & Theses Global	

Clinical Trial Registry Search Overview

Last searched 25 November 2019

ClinicalTrials.gov

- 565 Studies found for: outpatient | Completed, Suspended, Terminated Studies | Cardiovascular Diseases OR heart disease | Adult
- Applied Filters: Completed, Suspended, Terminated, Adult (18–64)
- https://clinicaltrials.gov/ct2/results?term=outpatient&cond=Cardiovascular+Diseases+OR+heart+disease&recrs=g&recrs=h&recrs=e&age_v=&age=1&gndr=&type=&rslt=&Search=Apply

WHO International Clinical Trials Registry Platform

Search (cardiovascular disease OR heart disease) AND outpatient – Recruitment status is: ALL

Recruitment status	Prospective Registration	Main ID		Public Title	Date of Registration	Results available
Not Recruiting	No	JPRN-UMIN000021454		Multi-center study for evaluating distribution of RHI in Japanese subjects without cardiovascular diseases for estimating clinical reference value of RHI in Japan	12/03/2016	
Not recruiting	Yes	ACTRN12614000527662		Sedentary behaviour in people with cardiovascular disease: a pilot randomised controlled trial	19/05/2014	
Recruiting	No	CTRI/2013/08/003928		A study on risk of developing heart disease in patients with skin disease called psoriasis marked by red, itchy, scaly patches.	29-08-2013	

© 2021 Singh K et al. *JAMA Network Open*.

1. Allen K, Hazelett S, Jarjoura D, Hua K, Wright K, Weinhardt J, et al. A Randomized Trial Testing the Superiority of a Postdischarge Care Management Model for Stroke Survivors. Journal of Stroke and Cerebrovascular Diseases. 2009 Nov;18(6):443–52.

2. Torres-Arreola L del P, Doubova Dubova SV, Hernandez SF, Torres-Valdez LE, Constantino-Casas NP, Garcia-Contreras F, et al. Effectiveness of two rehabilitation strategies provided by nurses for stroke patients in Mexico: *Effectiveness of rehabilitation in stroke*. Journal of Clinical Nursing. 2009 Nov;18(21):2993–3002.

3. Bakas T, Farran CJ, Austin JK, Given BA, Johnson EA, Williams LS. Stroke Caregiver Outcomes from the Telephone Assessment and Skill-Building Kit (TASK). Topics in Stroke Rehabilitation. 2009 Mar;16(2):105–21.

4. Battersby M, Hoffmann S, Cadilhac D, Osborne R, Lalor E, Lindley R. 'Getting your Life Back on Track after Stroke': A Phase II Multi-Centered, Single-Blind, Randomized, Controlled Trial of the Stroke Self-Management Program Vs. the Stanford Chronic Condition Self-Management Program or Standard Care in Stroke Survivors. International Journal of Stroke. 2009 Apr;4(2):137–44.

5. Boysen G, Krarup L-H, Zeng X, Oskedra A, Korv J, Andersen G, et al. ExStroke Pilot Trial of the effect of repeated instructions to improve physical activity after ischaemic stroke: a multinational randomised controlled clinical trial. BMJ. 2009 Jul 22;339(jul20 3):b2810–b2810.

6. Brotons C, Falces C, Alegre J, Ballarín E, Casanovas J, Catà T, et al. Randomized Clinical Trial of the Effectiveness of a Home-Based Intervention in Patients With Heart Failure: The IC-DOM Study. Revista Española de Cardiología (English Edition). 2009 Apr;62(4):400–8.

7. Brubaker PH, Moore JB, Stewart KP, Wesley DJ, Kitzman DW. Endurance Exercise Training in Older Patients with Heart Failure: Results from a Randomized, Controlled, Single-Blind Trial: EXERCISE TRAINING IN ELDERLY PATIENTS WITH HF. Journal of the American Geriatrics Society. 2009 Nov;57(11):1982–9.

8. Dar O, Riley J, Chapman C, Dubrey SW, Morris S, Rosen SD, et al. A randomized trial of home telemonitoring in a typical elderly heart failure population in North West London: results of the Home-HF study. European Journal of Heart Failure. 2009 Mar;11(3):319–25.

9. Forster A, Young J, Green J, Patterson C, Wanklyn P, Smith J, et al. Structured re-assessment system at 6 months after a disabling stroke: a randomised controlled trial with resource use and cost study. Age and Ageing. 2009 Sep;38(5):576–83.

10. Freedland KE, Skala JA, Carney RM, Rubin EH, Lustman PJ, Dávila-Román VG, et al. Treatment of Depression After Coronary Artery Bypass Surgery: A Randomized Controlled Trial. Arch Gen Psychiatry. 2009 Apr 1;66(4):387.

11. Giallauria F, Lucci R, D'Agostino M, Vitelli A, Maresca L, Mancini M, et al. Two-year multicomprehensive secondary prevention program: favorable effects on cardiovascular

© 2021 Singh K et al. *JAMA Network Open*.

functional capacity and coronary risk profile after acute myocardial infarction. Journal of Cardiovascular Medicine. 2009 Oct;10(10):772–780.

12. Jolly K, Taylor RS, Lip GYH, Davies M, Davis R, Mant J, et al. A randomized trial of the addition of home-based exercise to specialist heart failure nurse care: the Birmingham Rehabilitation Uptake Maximisation study for patients with Congestive Heart Failure (BRUM-CHF) study. European Journal of Heart Failure. 2009 Feb;11(2):205–13.

13. Joubert J, Reid C, Barton D, Cumming T, McLean A, Joubert L, et al. Integrated care improves risk-factor modification after stroke: initial results of the Integrated Care for the Reduction of Secondary Stroke model. Journal of Neurology, Neurosurgery & Psychiatry. 2009 Mar 1;80(3):279–84.

14. Parry M, Watt-Watson J, Hodnett E, Tranmer J, Dennis C-L, Brooks D. Cardiac Home Education and Support Trial (CHEST): A pilot study. Canadian Journal of Cardiology. 2009 Dec;25(12):e393–8.

15. Sahebalzamani M, Aliloo L, Shakibi A. The efficacy of self-care education on rehabilitation of stroke patients. :5.

16. Stone PW. Nurse-led heart failure management improved quality of life and was cost-effectiveCommentary. Evidence-Based Nursing. 2009 Apr 1;12(2):59–59.

17. Wakefield BJ, Holman JE, Ray A, Scherubel M, Burns TL, Kienzle MG, et al. Outcomes of a home telehealth intervention for patients with heart failure. J Telemed Telecare. 2009 Jan;15(1):46–50.

18. DeWalt DA, Broucksou KA, Hawk V, Baker DW, Schillinger D, Ruo B, et al. Comparison of a one-time educational intervention to a teach-to-goal educational intervention for self-management of heart failure: design of a randomized controlled trial. BMC Health Serv Res. 2009 Dec;9(1):99.

19. Zhao Y, Wong FKY. Effects of a postdischarge transitional care programme for patients with coronary heart disease in China: a randomised controlled trial. Journal of Clinical Nursing. 2009 Sep;18(17):2444–55.

20. Hawkes AL, Atherton J, Taylor CB, Scuffham P, Eadie K, Miller NH, et al. Randomised controlled trial of a secondary prevention program for myocardial infarction patients ('ProActive Heart'): study protocol. BMC Cardiovasc Disord. 2009 Dec;9(1):16.

21. Rollman BL, Belnap BH, LeMenager MS, Mazumdar S, Schulberg HC, Reynolds CF. The Bypassing the Blues Treatment Protocol: Stepped Collaborative Care for Treating Post-CABG Depression: Psychosomatic Medicine. 2009 Feb;71(2):217–30.

22. Aguado O, Morcillo C, Delàs J, Rennie M, Bechich S, Schembari A, et al. Long-term implications of a single home-based educational intervention in patients with heart failure. Heart & Lung: The Journal of Cardiopulmonary and Acute Care. 2010 Nov 1;39(6):S14–22.

© 2021 Singh K et al. *JAMA Network Open*.

23. Andryukhin A, Frolova E, Vaes B, Degryse J. The impact of a nurse-led care programme on events and physical and psychosocial parameters in patients with heart failure with preserved ejection fraction: A randomized clinical trial in primary care in Russia. European Journal of General Practice. 2010 Dec;16(4):205–14.

24. Beckie TM, Beckstead JW. Predicting Cardiac Rehabilitation Attendance in a Gender-Tailored Randomized Clinical Trial: Journal of Cardiopulmonary Rehabilitation and Prevention. 2010 May;30(3):147–56.

25. Chumbler NR, Rose DK, Griffiths P, Quigley P, McGee-Hernandez N, Carlson KA, et al. Study protocol: home-based telehealth stroke care: a randomized trial for veterans. Trials. 2010 Dec;11(1):74.

26. DeVon HA, Rankin SH, Paul SM, Ochs AL. The Know & Go! program improves knowledge for patients with coronary heart disease in pilot testing. Heart & Lung. 2010 Nov;39(6):S23–33.

27. Fan H, Shi H, Jin W, Zhu Y, Huang D, Yan Y, et al. [Effects of integrated disease management program on the outcome of patients with heart failure]. Zhonghua Xin Xue Guan Bing Za Zhi. 2010 Jul;38(7):592–6.

28. Kowloon Hospital, Hong Kong. Patient Engagement Program for Stroke - Pilot Study [Internet]. clinicaltrials.gov; 2010 Apr [cited 2020 Aug 25]. Report No.: NCT01112488. Available from: https://clinicaltrials.gov/ct2/show/NCT01112488

29. Gillham S, Endacott R. Impact of enhanced secondary prevention on health behaviour in patients following minor stroke and transient ischaemic attack: a randomized controlled trial. Clin Rehabil. 2010 Sep;24(9):822–30.

30. Guidetti S, Andersson K, Andersson M, Tham K, Koch LV. Client-centred self-care intervention after stroke: A feasibility study. Scandinavian Journal of Occupational Therapy. 2010 Dec;17(4):276–85.

31. Johnston SC, Sidney S, Hills NK, Grosvenor D, Klingman JG, Bernstein A, et al. Standardized discharge orders after stroke: Results of the quality improvement in stroke prevention (QUISP) cluster randomized trial. Ann Neurol. 2010;NA-NA.

32. MacKay-Lyons M, Gubitz G, Giacomantonio N, Wightman H, Marsters D, Thompson K, et al. Program of rehabilitative exercise and education to avert vascular events after non-disabling stroke or transient ischemic attack (PREVENT Trial): a multi-centred, randomised controlled trial. BMC Neurol. 2010 Dec;10(1):122.

33. Mainardi L, Iazzolino E, Asteggiano R, Lusardi R, Varbella F, Sasso L, et al. Confronto tra gestione infermieristica telefonica ed ambulatoriale integrata dei pazienti affetti da scompenso cardiaco cronico in un vasto territorio piemontese. 2010;11:8.

34. Marsden D, Quinn R, Pond N, Golledge R, Neilson C, White J, et al. A multidisciplinary group programme in rural settings for community-dwelling chronic stroke survivors and their carers: a pilot randomized controlled trial. Clin Rehabil. 2010 Apr;24(4):328–41.
© 2021 Singh K et al. *JAMA Network Open*.

35. Mosca L, Christian AH, Mochari-Greenberger H, Kligfield P, Smith SC. A Randomized Clinical Trial of Secondary Prevention Among Women Hospitalized with Coronary Heart Disease. Journal of Women's Health. 2010 Feb;19(2):195–202.

36. Representing the CAM2 Project working group, Muñiz J, Gómez-Doblas JJ, Santiago-Pérez MI, Lekuona-Goya I, Murga-Eizagaetxebarría N, et al. The effect of post-discharge educational intervention on patients in achieving objectives in modifiable risk factors six months after discharge following an episode of acute coronary syndrome, (CAM-2 Project): a randomized controlled trial. Health Qual Life Outcomes. 2010 Dec;8(1):137.

37. Smeulders ESTF, Van Haastregt JCM, Ambergen T, Uszko-Lencer NHKM, Janssen-Boyne JJJ, Gorgels APM, et al. Nurse-led self-management group programme for patients with congestive heart failure: randomized controlled trial: Self-management of heart failure. Journal of Advanced Nursing. 2010 May 13;66(7):1487–99.

38. Shyu Y-IL, Kuo L-M, Chen M-C, Chen S-T. A clinical trial of an individualised intervention programme for family caregivers of older stroke victims in Taiwan: Family caregiver intervention programme. Journal of Clinical Nursing. 2010 Apr 1;19(11–12):1675–85.

39. Papadopoulou, E. Cardiac failure patients telephone intervention program by specialized trained nurse and the impact on the occurrence of patient readmissions and cardiac mortality rate. Heart Surgery Forum 2010. 2010;

40. Kirchberger I, Meisinger C, Seidl H, Wende R, Kuch B, Holle R. Nurse-based case management for aged patients with myocardial infarction: study protocol of a randomized controlled trial. BMC Geriatr. 2010 Dec;10(1):29.

41. Rochette A, Korner-Bitensky N, Bishop D, Teasell R, White C, Bravo G, et al. Study protocol of the YOU CALL - WE CALL TRIAL: impact of a multimodal support intervention after a "mild" stroke. BMC Neurol. 2010 Dec;10(1):3.

42. Carrington MJ, Stewart S. Bridging the gap in heart failure prevention: rationale and design of the Nurse-led Intervention for Less Chronic Heart Failure (NIL-CHF) Study. European Journal of Heart Failure. 2010;12(1):82–8.

43. Böhmer A, Wieser M, Weywar I, Frauendorfer H, Kronik G. Das „Kremser Modell": Erfolgreiches Disease- Management zur Betreuung von Patienten mit Herzinsuffizienz. :11.

44. Bowles KH, Hanlon AL, Glick HA, Naylor MD, O'Connor M, Riegel B, et al. Clinical Effectiveness, Access to, and Satisfaction with Care Using a Telehomecare Substitution Intervention: A Randomized Controlled Trial. International Journal of Telemedicine and Applications. 2011;2011:1–13.

45. Bredie SJH, Fouwels AJ, Wollersheim H, Schippers GM. Effectiveness of Nurse Based Motivational Interviewing for Smoking Cessation in High Risk Cardiovascular Outpatients: A Randomized Trial. European Journal of Cardiovascular Nursing. 2011 Sep;10(3):174–9.

© 2021 Singh K et al. *JAMA Network Open*.

46. Dilles A, Heymans V, Martin S, Droogné W, Denhaerynck K, De Geest S. Comparison of a Computer Assisted Learning Program to Standard Education Tools in Hospitalized Heart Failure Patients. European Journal of Cardiovascular Nursing. 2011 Sep;10(3):187–93.

47. Domingo M, Lupón J, González B, Crespo E, López R, Ramos A, et al. Noninvasive Remote Telemonitoring for Ambulatory Patients With Heart Failure: Effect on Number of Hospitalizations, Days in Hospital, and Quality of Life. CARME (CAtalan Remote Management Evaluation) Study. Revista Española de Cardiología (English Edition). 2011 Apr;64(4):277–85.

48. Bossone E, Limongelli G, Malizia G, Ferrara F, Vriz O, Citro R, et al. The T.O.S.CA. Project: research, education and care. Monaldi Arch Chest Dis. 2011 Dec;76(4):198–203.

49. Fjærtoft H, Rohweder G, Indredavik B. Stroke Unit Care Combined With Early Supported Discharge Improves 5-Year Outcome: A Randomized Controlled Trial. Stroke. 2011 Jun;42(6):1707–11.

50. Research Report Abstracts. Physiotherapy. 2011 Jun;97:eS18–1415.

51. Huffman JC, Mastromauro CA, Sowden GL, Wittmann C, Rodman R, Januzzi JL. A Collaborative Care Depression Management Program for Cardiac Inpatients: Depression Characteristics and In-Hospital Outcomes. Psychosomatics. 2011 Jan;52(1):26–33.

52. Abstracts. International Journal of Stroke. 2011 Sep;6(1_suppl):1–34.

53. Leventhal M, Denhaerynck K, Brunner-La Rocca H, Burnand B, Conca-Zeller A, Bernasconi A, et al. Swiss Interdisciplinary Management Programme for Heart Failure (SWIM-HF): A randomised controlled trial study of an outpatient inter-professional management programme for heart failure patients in Switzerland. Swiss Med Wkly [Internet]. 2011 Mar 8 [cited 2020 Aug 27]; Available from: http://doi.emh.ch/smw.2011.13171

54. Levine DA. Improving Care After Myocardial Infarction Using a 2-Year Internet-Delivered Intervention: The Department of Veterans Affairs Myocardial Infarction–Plus Cluster-Randomized Trial. Arch Intern Med. 2011 Nov 28;171(21):1910.

55. McKenna. Research Report Abstracts. Physiotherapy. 2011 Jun;97:eS18–1415.

56. Oerkild B, Frederiksen M, Hansen JF, Simonsen L, Skovgaard LT, Prescott E. Home-based cardiac rehabilitation is as effective as centre-based cardiac rehabilitation among elderly with coronary heart disease: results from a randomised clinical trial. Age and Ageing. 2011 Jan;40(1):78–85.

57. Otsu H, Moriyama M. Effectiveness of an educational self-management program for outpatients with chronic heart failure: Self-management of chronic heart failure. Japan Journal of Nursing Science. 2011 Dec;8(2):140–52.

58. Shanmugasegaram S, Kovacs AH, Ardern C, Oh P, Stewart DE, Grace SL. 430 Relationships among physical activity and correlates of depressive symptoms in female cardiac patients. Canadian Journal of Cardiology. 2011 Sep;27(5):S217–8.

© 2021 Singh K et al. *JAMA Network Open*.

59. Seto E, Leonard KJ, Cafazzo JA, Masino C, Barnsley J, Ross HJ. MOBILE PHONE-BASED REMOTE PATIENT MONITORING IMPROVES HEART FAILURE MANAGEMENT AND OUTCOMES: A RANDOMIZED CONTROLLED TRIAL. Journal of the American College of Cardiology. 2011 Apr;57(14):E1260.

60. Muzdi. Research Report Abstracts. Physiotherapy. 2011 Jun;97:eS18–1415.

61. Persson-2011-Results of a randomized clinical.pdf [Internet]. [cited 2020 Aug 27]. Available from: https://regroup-production.s3.amazonaws.com/documents/ReviewReference/149745317/Persson-2011-Results%20of%20a%20randomized%20clinical.pdf?AWSAccessKeyId=AKIAJBZQODCMKJA4H7DA&Expires=1598541814&Signature=mbsrynJoJNdJJtWZDLyNVoIJi%2F0%3D

62. Markle-Reid M, Orridge C, Weir R, Browne G, Gafni A, Lewis M, et al. Interprofessional Stroke Rehabilitation for Stroke Survivors Using Home Care. Can J Neurol Sci. 2011 Mar;38(2):317–34.

63. Stauffer BD. Effectiveness and Cost of a Transitional Care Program for Heart Failure: A Prospective Study With Concurrent Controls. Arch Intern Med. 2011 Jul 25;171(14):1238.

64. Stewart S, Carrington MJ, Marwick T, Davidson PM, Macdonald P, Horowitz J, et al. The WHICH? trial: rationale and design of a pragmatic randomized, multicentre comparison of home- vs. clinic-based management of chronic heart failure patients. European Journal of Heart Failure. 2011 Aug;13(8):909–16.

65. Wang S-P, Lin L-C, Lee C-M, Wu S-C. Effectiveness of a Self-Care Program in Improving Symptom Distress and Quality of Life in Congestive Heart Failure Patients: A Preliminary Study. Journal of Nursing Research. 2011 Dec;19(4):257–66.

66. Gersh BJ. Secondary Prevention After Coronary Artery Bypass Graft Surgery: Findings of a National Randomized Controlled Trial and Sustained Society-Led Incorporation Into Practice. Yearbook of Cardiology. 2012 Jan;2012:240–2.

67. Tsuchihashi-Makaya M, Matsuo H, Kakinoki S, Takechi S, Tsutsui H. Rationale and design of the Japanese Heart Failure Outpatients Disease Management and Cardiac Evaluation (J-HOMECARE). Journal of Cardiology. 2011 Sep;58(2):165–72.

68. de Vries AE, de Jong RM, van der Wal MH, Jaarsma T, van Dijk RB, Hillege HL. The value of INnovative ICT guided disease management combined with Telemonitoring in OUtpatient clinics for Chronic Heart failure patients. Design and methodology of the IN TOUCH study: a multicenter randomised trial. BMC Health Serv Res. 2011 Dec;11(1):167.

69. Graven C, Brock K, Hill K, Ames D, Cotton S, Joubert L. From rehabilitation to recovery: protocol for a randomised controlled trial evaluating a goal-based intervention to reduce depression and facilitate participation post-stroke. BMC Neurol. 2011 Dec;11(1):73.

70. Shah BR, Adams M, Peterson ED, Powers B, Oddone EZ, Royal K, et al. Secondary Prevention Risk Interventions Via Telemedicine and Tailored Patient Education (SPRITE): A

© 2021 Singh K et al. *JAMA Network Open*.

Randomized Trial to Improve Postmyocardial Infarction Management. Circ Cardiovasc Qual Outcomes. 2011 Mar;4(2):235–42.

71. Amariles P, Sabater-Hernández D, García-Jiménez E, Rodríguez-Chamorro MÁ, Prats-Más R, Marín-Magán F, et al. Effectiveness of Dader Method for Pharmaceutical Care on Control of Blood Pressure and Total Cholesterol in Outpatients with Cardiovascular Disease or Cardiovascular Risk: EMDADER-CV Randomized Controlled Trial. JMCP. 2012 May 1;18(4):311–23.

72. Barker A, Barlis P, Berlowitz D, Page K, Jackson B, Lim WK. Pharmacist directed home medication reviews in patients with chronic heart failure: A randomised clinical trial. International Journal of Cardiology. 2012 Aug;159(2):139–43.

73. Blumenthal JA, Sherwood A, Babyak MA, Watkins LL, Smith PJ, Hoffman BM, et al. Exercise and Pharmacological Treatment of Depressive Symptoms in Patients With Coronary Heart Disease. Journal of the American College of Cardiology. 2012 Sep;60(12):1053–63.

74. Blumenthal J. Effects of Exercise Training on Health Status in Patients With Chronic Heart Failure: HF-ACTION Randomized Controlled Trial. :9.

75. Cawthon C, Walia S, Osborn CY, Niesner KJ, Schnipper JL, Kripalani S. Improving Care Transitions: The Patient Perspective. Journal of Health Communication. 2012 Oct;17(sup3):312–24.

76. Chaiyawat P, Kulkantrakorn K. Randomized controlled trial of home rehabilitation for patients with ischemic stroke: impact upon disability and elderly depression: Home rehabilitation for ischemic stroke. Psychogeriatrics. 2012 Sep;12(3):193–9.

77. Chan SSC, Leung DYP, Wong DCN, Lau C-P, Wong VT, Lam T-H. A randomized controlled trial of stage-matched intervention for smoking cessation in cardiac out-patients: Stage-matched smoking cessation intervention. Addiction. 2012 Apr;107(4):829–37.

78. Chi C, Chen H. DAILY-BASED SELF-MANAGEMENT FOR NON-HOSPITALISED HEART FAILURE PATIENTS IMPROVE PROGNOSIS. Heart. 2012 Oct;98(Suppl 2):E231–2.

79. Donner Alves F. ORIENTACION NUTRICIONAL, CONOCIMIENTO Y CALIDAD DE LA DIETA EN LA INSUFICIENCIA CARDÍACA; ENSAYO CLÍNICO ALEATORIZADO. NUTRICION HOSPITALARIA. 2012 Mar 1;(2):441–8.

80. Faulkner J, Lambrick D, Woolley B, Stoner L, Wong L. Health-enhancing physical activity programme (HEPAP) for transient ischaemic attack and non-disabling stroke: recruitment and compliance. 2012;125(1364):9.

81. Franklin BA. Multifactorial cardiac rehabilitation did not reduce mortality or morbidity after acute myocardial infarction. Ann Intern Med. 2012 Jul 17;157(2):JC1.

82. Furze G, Cox H, Morton V, Chuang L-H, Lewin RJP, Nelson P, et al. Randomized controlled trial of a lay-facilitated angina management programme: RCT of a lay-facilitated angina management programme. Journal of Advanced Nursing. 2012 Oct;68(10):2267–79.

© 2021 Singh K et al. *JAMA Network Open*.

83. He Y, Lu Z, Gu Y, Pan J, Gao M, Wei M. [Impact of multifactor intensive intervention on self management, risk factor control and outcome of post percutaneous transluminal coronary intervention patients]. Zhonghua Xin Xue Guan Bing Za Zhi. 2012 Dec;40(12):1037–40.

84. Uysal H, Özcan Ş. The effect of individual training and counselling programme for patients with myocardial infarction over patients' quality of life: Training programme for myocardial infarction. Int J Nurs Pract. 2012 Oct;18(5):445–53.

85. IJzelenberg W, Hellemans IM, van Tulder MW, Heymans MW, Rauwerda JA, van Rossum AC, et al. The effect of a comprehensive lifestyle intervention on cardiovascular risk factors in pharmacologically treated patients with stable cardiovascular disease compared to usual care: a randomised controlled trial. BMC Cardiovasc Disord. 2012 Dec;12(1):71.

86. Kripalani S, Roumie CL, Dalal AK, Cawthon C, Businger A, Eden SK, et al. Effect of a Pharmacist Intervention on Clinically Important Medication Errors After Hospital Discharge. :12.

87. Alexa ID, Axinte CI. An underdiagnosed cause of CK-MB elevation in absence of acute coronary syndrome in elderly patients. European Geriatric Medicine. 2012 Sep;3:S41.

88. UK Stroke Forum 2012 Conference, North Yorkshire 4–6 December 2012. International Journal of Stroke. 2012 Dec;7(2_suppl):1–79.

89. Rodríguez-Gázquez M de los Á, Arredondo-Holguín E, Herrera-Cortés R. Effectiveness of an educational program in nursing in the self-care of patients with heart failure: randomized controlled trial. Rev Latino-Am Enfermagem. 2012 Apr;20(2):296–306.

90. European Stroke Conference, Stroke Meeting for Nurses P Speech and Occupational Therapists, Study/Research Assistant, editors. Abstract book. Basel: Karger; 2012.

91. Sturchio A, Di Gianni A, Campana B, Genua M, Storti M, Di Iasi G, et al. Coronary Artery RIsk MAnagement Programme (CARIMAP) Delivered by a Rehabilitation Day-Hospital: IMPACT ON PATIENTS WITH CORONARY ARTERY DISEASE. Journal of Cardiopulmonary Rehabilitation and Prevention. 2012;32(6):386–93.

92. Wang W, Chair SY, Thompson DR, Twinn SF. Effects of home-based rehabilitation on health-related quality of life and psychological status in Chinese patients recovering from acute myocardial infarction. Heart & Lung. 2012 Jan;41(1):15–25.

93. West RR, Jones DA, Henderson AH. Rehabilitation after myocardial infarction trial (RAMIT): multi-centre randomised controlled trial of comprehensive cardiac rehabilitation in patients following acute myocardial infarction. Heart. 2012 Apr 15;98(8):637–44.

94. Abstracts and Index of Authors. Pharmacotherapy: The Journal of Human Pharmacology and Drug Therapy. 2012;32(10):e178–320.

95. R A, J C, Dr W, K W, R T, S L, et al. Care guides: employing nonclinical laypersons to help primary care teams manage chronic disease. J Ambul Care Manage. 2012 Jan 1;35(1):27–37.

© 2021 Singh K et al. *JAMA Network Open*.

96. Wu C-JJ, Chang AM, Courtney M, Kostner K. Peer supporters for cardiac patients with diabetes: a randomized controlled trial. Int Nurs Rev. 2012 Sep;59(3):345–52.

97. Kärner A, Nilsson S, Jaarsma T, Andersson A, Wiréhn A-B, Wodlin P, et al. The effect of problem-based learning in patient education after an event of CORONARY heart disease – a randomised study in PRIMARY health care: design and methodology of the COR-PRIM study. BMC Fam Pract. 2012 Dec;13(1):110.

98. Tiessen AH, Smit AJ, Broer J, Groenier KH, van der Meer K. Randomized controlled trial on cardiovascular risk management by practice nurses supported by self-monitoring in primary care. BMC Fam Pract. 2012 Dec;13(1):90.

99. UK Stroke Forum 2012 Conference, North Yorkshire 4–6 December 2012. International Journal of Stroke. 2012 Dec;7(2_suppl):1–79.

100. Lambert-Kerzner A, Del Giacco EJ, Fahdi IE, Bryson CL, Melnyk SD, Bosworth HB, et al. Patient-Centered Adherence Intervention After Acute Coronary Syndrome Hospitalization. Circ Cardiovasc Qual Outcomes. 2012 Jul;5(4):571–6.

101. Ivers NM, Schwalm J-D, Grimshaw JM, Witteman H, Taljaard M, Zwarenstein M, et al. Delayed educational reminders for long-term medication adherence in ST-elevation myocardial infarction (DERLA-STEMI): protocol for a pragmatic, cluster-randomized controlled trial. Implement Sci. 2012 Jun 9;7:54.

102. Sackley CM, Burton CR, Herron-Marx S, Lett K, Mant J, Roalfe AK, et al. A cluster randomised controlled trial of an occupational therapy intervention for residents with stroke living in UK care homes (OTCH): study protocol. BMC Neurol. 2012 Dec;12(1):52.

103. Bernal DDL, Stafford L, Bereznicki LRE, Castelino RL, Davidson PM, Peterson GM. Home medicines reviews following acute coronary syndrome: study protocol for a randomized controlled trial. Trials. 2012 Dec;13(1):30.

104. Leemrijse CJ, van Dijk L, Jørstad HT, Peters RJG, Veenhof C. The effects of Hartcoach, a life style intervention provided by telephone on the reduction of coronary risk factors: a randomised trial. BMC Cardiovasc Disord. 2012 Dec;12(1):47.

105. Saywell N, Vandal AC, Brown P, Hanger HC, Hale L, Mudge S, et al. Telerehabilitation to improve outcomes for people with stroke: study protocol for a randomised controlled trial. Trials. 2012 Dec;13(1):233.

106. Forster A, Young J, Nixon J, Kalra L, Smithard D, Patel A, et al. A Cluster Randomized Controlled Trial of a Structured Training Programme for Caregivers of Inpatients after Stroke (TRACS). International Journal of Stroke. 2012 Jan;7(1):94–9.

107. Williams_2012_Working with CALD groups.pdf [Internet]. [cited 2020 Aug 27]. Available from: https://regroup-production.s3.amazonaws.com/documents/ReviewReference/149744829/Williams_2012_Working%20with%20CALD%20groups.PDF?AWSAccessKeyId=AKIAJBZQODCMKJA4H7DA&Expires=1598554123&Signature=SFWddnl%2BseLA%2FIphwC86%2BaRNbbc%3D

© 2021 Singh K et al. *JAMA Network Open*.

108. Aguirrezabal A, Duarte E, Rueda N, Cervantes C, Marco E, Escalada F. Effects of information and training provision in satisfaction of patients and carers in stroke rehabilitation. NRE. 2013 Dec 28;33(4):639–47.

109. Ansinelli DJ. The Effectiveness of a Structured Telephone Support Program for Rural Patients with Heart Failure [Internet] [DNP]. West Virginia University Libraries; 2013 [cited 2020 Aug 28]. Available from: https://researchrepository.wvu.edu/etd/475

110. Carrington MJ, Chan Y-K, Calderone A, Scuffham PA, Esterman A, Goldstein S, et al. A Multicenter, Randomized Trial of a Nurse-Led, Home-Based Intervention for Optimal Secondary Cardiac Prevention Suggests Some Benefits for Men but Not for Women: The Young at Heart Study. Circ Cardiovasc Qual Outcomes. 2013 Jul;6(4):379–89.

111. Cartwright M, Hirani SP, Rixon L, Beynon M, Doll H, Bower P, et al. Effect of telehealth on quality of life and psychological outcomes over 12 months (Whole Systems Demonstrator telehealth questionnaire study): nested study of patient reported outcomes in a pragmatic, cluster randomised controlled trial. BMJ. 2013 Feb 26;346(feb26 2):f653–f653.

112. çavusoglu Yüksel, Zoghi Mehdi, Eren Mehmet, Bozçali Evin, Kozdag Güliz, Sentürk Tuncay, et al. Abstract 17804: Does Cardiologist Lead Enhanced Heart Failure Education and Follow-up Program Affect Cardiovascular Mortality Rate?: Hit-Point. Circulation. 2013 Nov 26;128(suppl_22):A17804–A17804.

113. Chen Y-H, Lin Y-H, Hung C-S, Huang C-C, Yeih D-F, Chuang P-Y, et al. Clinical Outcome and Cost-Effectiveness of a Synchronous Telehealth Service for Seniors and Nonseniors with Cardiovascular Diseases: Quasi-Experimental Study. Journal of Medical Internet Research. 2013;15(4):e87.

114. Drummond A, Whitehead P, Fellows K, Sprigg N, Sampson C, Edwards C, et al. Occupational therapy predischarge home visits for patients with a stroke (HOVIS): results of a feasibility randomized controlled trial. Clin Rehabil. 2013 May;27(5):387–97.

115. Eames S, Hoffmann T, Worrall L, Read S, Wong A. Randomised controlled trial of an education and support package for stroke patients and their carers. BMJ Open. 2013;3(5):e002538.

116. Flemming KD, Allison TG, Covalt JL, Herzig DE, Brown RD. Utility of a Post-Hospitalization Stroke Prevention Program Managed by Nurses. Hospital Practice. 2013 Aug;41(3):70–9.

117. EuroHeartCare 2013. European Journal of Cardiovascular Nursing. 2013 Apr;12(1_suppl):S1–84.

118. Kong J-H, Ha Y. Effects of a Smoking Cessation Program including Telephone Counseling and Text Messaging using Stages of Change for Outpatients after a Myocardial Infarction. J Korean Acad Nurs. 2013;43(4):557.

119. EuroHeartCare 2013. European Journal of Cardiovascular Nursing. 2013 Apr;12(1_suppl):S1–84.

© 2021 Singh K et al. *JAMA Network Open*.

120. Mok VKF, Sit JWH, Tsang ASM, Chair SY, Cheng TL, Chiang C. A Controlled Trial of a Nurse Follow-up Dietary Intervention on Maintaining a Heart-Healthy Dietary Pattern Among Patients After Myocardial Infarction: The Journal of Cardiovascular Nursing. 2013;28(3):256–66.

121. Nundy S, Razi RR, Dick JJ, Smith B, Mayo A, O'Connor A, et al. A Text Messaging Intervention to Improve Heart Failure Self-Management After Hospital Discharge in a Largely African-American Population: Before-After Study. J Med Internet Res. 2013 Mar 11;15(3):e53.

122. Mengensatzproduktion S, Basel DRD, Basel DRD. 10th International Congress on Coronary Artery Disease (ICCAD). Florence, Italy, October 13–16, 2013: Abstracts. CRD. 2013;126(Suppl. 2):1–530.

123. Poortaghi S, Baghernia A, Golzari SE, Safayian A, Atri SB. The effect of home-based cardiac rehabilitation program on self efficacy of patients referred to cardiac rehabilitation center. BMC Res Notes. 2013;6(1):287.

124. Rinfret S, Rodés-Cabau J, Bagur R, Déry J-P, Dorais M, Larose É, et al. Telephone contact to improve adherence to dual antiplatelet therapy after drug-eluting stent implantation. Heart. 2013 Apr 15;99(8):562–9.

125. Sabariego C, Barrera AE, Neubert S, Stier-Jarmer M, Bostan C, Cieza A. Evaluation of an ICF-based patient education programme for stroke patients: A randomized, single-blinded, controlled, multicentre trial of the effects on self-efficacy, life satisfaction and functioning. British Journal of Health Psychology. 2013;18(4):707–28.

126. Schou M, Investigators on behalf of N, Gislason GG, Investigators on behalf of N, Videbaek L, Investigators on behalf of N, et al. Long term adherence in primary care versus a specialized heart failure clinic for outpatients with systolic heart failure. Eur Heart J [Internet]. 2013 Aug 1 [cited 2020 Aug 28];34(suppl_1). Available from: https://academic.oup.com/eurheartj/article/34/suppl_1/806/2860254

127. Shao J-H, Chang AM, Edwards H, Shyu Y-IL, Chen S-H. A randomized controlled trial of self-management programme improves health-related outcomes of older people with heart failure. J Adv Nurs. 2013 Mar;n/a-n/a.

128. Sun-2013-Curative effects on standardized management.pdf [Internet]. [cited 2020 Sep 2]. Available from: https://regroup-production.s3.amazonaws.com/documents/ReviewReference/149750372/Sun-2013-Curative%20effects%20on%20standardized%20management.pdf?AWSAccessKeyId=AKIAJBZQODCMKJA4H7DA&Expires=1599047759&Signature=2COaDBm6y2qRAPdwJHCnDu6dwPA%3D

129. Tsuchihashi-Makaya M, Matsuo H, Kakinoki S, Takechi S, Kinugawa S, Tsutsui H, et al. Home-Based Disease Management Program to Improve Psychological Status in Patients With Heart Failure in Japan. Circ J. 2013;77(4):926–33.

© 2021 Singh K et al. *JAMA Network Open*.

130. Turton AJ, Cunningham P, Heron E, van Wijck F, Sackley C, Rogers C, et al. Home-based reach-to-grasp training for people after stroke: study protocol for a feasibility randomized controlled trial. Trials. 2013;14(1):109.

131. Aghakhani N, Sharif F, Khademvatan K, Rahbar N, Eghtedar S, Shojaei Motlagh V. 279 – The reduction in anxiety and depression by education of patients with myocardial infarction. European Psychiatry. 2013 Jan;28:1.

132. Newhouse RP, Dennison Himmelfarb C, Morlock L, Frick KD, Pronovost P, Liang Y. A phased cluster-randomized trial of rural hospitals testing a quality collaborative to improve heart failure care: organizational context matters. Med Care. 2013 May;51(5):396–403.

133. 42nd ESCP symposium on clinical pharmacy: implementation of pharmacy practice; Prague, Czech Republic, 16-18 October 2013. Int J Clin Pharm. 2013 Dec;35(6):1251–351.

134. Jorstad HT, Birgelen C von, Alings AMW, Liem A, Dantzig JM van, Jaarsma W, et al. Effect of a nurse-coordinated prevention programme on cardiovascular risk after an acute coronary syndrome: main results of the RESPONSE randomised trial. Heart. 2013 Oct 1;99(19):1421–30.

135. Visser MM, Heijenbrok-Kal MH, van 't Spijker A, Ribbers GM, Busschbach JJ. The effectiveness of problem solving therapy for stroke patients: study protocol for a pragmatic randomized controlled trial. BMC Neurol. 2013 Dec;13(1):67.

136. Bendelac H, Pathak A, Molinier L, Ruidavets J-B, Mayère A, Berry M, et al. Optimization of ambulatory monitoring of patients with heart failure using telecardiology (OSICAT). European Research in Telemedicine / La Recherche Européenne en Télémédecine. 2014 Dec;3(4):161–7.

137. Cockayne S, Pattenden J, Worthy G, Richardson G, Lewin R. Nurse facilitated Self-management support for people with heart failure and their family carers (SEMAPHFOR): a randomised controlled trial. International Journal of Nursing Studies. 2014 Sep;51(9):1207–13.

138. Demers C, Patterson C, Archer N, Coallier J, Strachan P, Keshavjee K, et al. A SIMPLE MULTI-COMPONENT INTERVENTION IMPROVES SELF MANAGEMENT IN HEART FAILURE. Canadian Journal of Cardiology. 2014 Oct;30(10):S201.

139. Dickson VV, Melkus GD, Katz S, Levine-Wong A, Dillworth J, Cleland CM, et al. Building skill in heart failure self-care among community dwelling older adults: Results of a pilot study. Patient Education and Counseling. 2014 Aug;96(2):188–96.

140. Dunbar SB, Butts B, Reilly CM, Gary RA, Higgins MK, Ferranti EP, et al. A pilot test of an integrated self-care intervention for persons with heart failure and concomitant diabetes. Nursing Outlook. 2014 Mar;62(2):97–111.

141. Eames S, Hoffmann TC, Phillips NF. Evaluating Stroke Patients' Awareness of Risk Factors and Readiness to Change Stroke Risk-Related Behaviors in a Randomized Controlled Trial. Topics in Stroke Rehabilitation. 2014 Mar 1;21(sup1):S52–62.

© 2021 Singh K et al. *JAMA Network Open*.

142. Fens M, Heugten C, Beusmans G, Metsemakers J, Kester A, Limburg M. Effect of a stroke-specific follow-up care model on the quality of life of stroke patients and caregivers: A controlled trial. J Rehabil Med. 2014;46(1):7–15.

143. González B, Lupón J, Domingo M del M, Cano L, Cabanes R, de Antonio M, et al. Educational level and self-care behaviour in patients with heart failure before and after nurse educational intervention. European Journal of Cardiovascular Nursing. 2014 Oct;13(5):459–65.

144. González-Guerrero JL, Alonso-Fernández T, García-Mayolín N, Gusi N, Ribera-Casado JM. Effectiveness of a follow-up program for elderly heart failure patients after hospital discharge. A randomized controlled trial. European Geriatric Medicine. 2014 Aug;5(4):252–7.

145. Gujral G, Winckel K, Nissen LM, Cottrell WN. Impact of community pharmacist intervention discussing patients' beliefs to improve medication adherence. Int J Clin Pharm. 2014 Oct;36(5):1048–58.

146. Hagglund. EuroHeartCare 2014. European Journal of Cardiovascular Nursing. 2014 Apr;13(1_suppl):S1–91.

147. Huang Tsuey-yuan, Huang Li-Yueh, Tsai Ming-Fen, Wang Chun-Li, Chen Chun-Chi, Lennie Terry A. Abstract 11189: Single Phone Call Follow-up Was an Effective Strategy to Improve Heart Failure Patients' Outcomes. Circulation. 2014 Nov 25;130(suppl_2):A11189–A11189.

148. Duke University. HF-ACTION Depression Education Substudy [Internet]. clinicaltrials.gov; 2014 Sep [cited 2020 Aug 27]. Report No.: NCT01794598. Available from: https://clinicaltrials.gov/ct2/show/NCT01794598

149. Acute Cardiovascular Care 2014. European Heart Journal: Acute Cardiovascular Care. 2014 Oct;3(2_suppl):1–236.

150. Oral Presentations in Order of Conference Program. International Journal of Stroke. 2014 Jul;9(1_suppl):1–26.

151. Koifman E, Grossman E, Elis A, Dicker D, Koifman B, Mosseri M, et al. Multidisciplinary rehabilitation program in recently hospitalized patients with heart failure and preserved ejection fraction: Rationale and design of a randomized controlled trial. American Heart Journal. 2014 Dec;168(6):830-837.e1.

152. Krishnamurthi R, Witt E, Barker-Collo S, McPherson K, Davis-Martin K, Bennett D, et al. Reducing Recurrent Stroke: Methodology of the Motivational Interviewing in Stroke (MIST) Randomized Clinical Trial. International Journal of Stroke. 2014 Jan;9(1):133–9.

153. Lourenço LB de A, Rodrigues RCM, Ciol MA, São-João TM, Cornélio ME, Dantas RAS, et al. A randomized controlled trial of the effectiveness of planning strategies in the adherence to medication for coronary artery disease. J Adv Nurs. 2014 Jul;70(7):1616–28.

154. Lubinskaya-2014-Cost-effectiveness of 2-year c.pdf [Internet]. [cited 2020 Aug 28]. Available from: https://regroup-

© 2021 Singh K et al. *JAMA Network Open*.

production.s3.amazonaws.com/documents/ReviewReference/149750514/Lubinskaya-2014-Cost-effectiveness%20of%202-year%20c.pdf?AWSAccessKeyId=AKIAJBZQODCMKJA4H7DA&Expires=1598613715&Signature=SuHv0Isfune%2FkiS70b1%2FwOe%2BIxs%3D

155. Luttik MLA, Jaarsma T, van Geel PP, Brons M, Hillege HL, Hoes AW, et al. Long-term follow-up in optimally treated and stable heart failure patients: primary care vs. heart failure clinic. Results of the COACH-2 study: Long-term follow-up in optimally treated and stable HF patients. Eur J Heart Fail. 2014 Nov;16(11):1241–8.

156. Mehralian H, Salehi S, Moghaddasi J, Amiri M, Rafiei H. The Comparison of the Effects of Education Provided by Nurses on the Quality of Life in Patients with Congestive Heart Failure (CHF) in Usual and Home-Visit Cares in Iran. GJHS. 2014 Apr 11;6(3):p256.

157. Melin-2014-Patient-centered home-based managem.pdf [Internet]. [cited 2020 Aug 28]. Available from: https://regroup-production.s3.amazonaws.com/documents/ReviewReference/149746842/Melin-2014-Patient-centered%20home-based%20managem.pdf?AWSAccessKeyId=AKIAJBZQODCMKJA4H7DA&Expires=1598613994&Signature=p5Ip4FzwSkkXMKQCVlMz4QbIMoQ%3D

158. Rood LM. Reducing 30-day Hospital Readmissions of the Heart Failure Patient: A Nurse Practitioner-Led Intervention. :60.

159. Saffi MAL, Polanczyk CA, Rabelo-Silva ER. Lifestyle interventions reduce cardiovascular risk in patients with coronary artery disease: A randomized clinical trial. European Journal of Cardiovascular Nursing. 2014 Oct;13(5):436–43.

160. Poster Session 1. European Journal of Heart Failure. 2014;16(s2):19–98.

161. Spatola CA, Manzoni G, Castelnuovo G, Malfatto G, Facchini M, Goodwin CL, et al. The ACTonHEART study: rationale and design of a randomized controlled clinical trial comparing a brief intervention based on Acceptance and Commitment Therapy to usual secondary prevention care of coronary heart disease. Health Qual Life Outcomes. 2014;12(1):22.

162. Thrift AG, Srikanth VK, Nelson MR, Kim J, Fitzgerald SM, Gerraty RP, et al. Risk Factor Management in Survivors of Stroke: A Double-Blind, Cluster-Randomized, Controlled Trial. International Journal of Stroke. 2014 Jul;9(5):652–7.

163. Villani A, Malfatto G, Compare A, Rosa FD, Bellardita L, Branzi G, et al. Clinical and psychological telemonitoring and telecare of high risk heart failure patients. J Telemed Telecare. 2014 Dec;20(8):468–75.

164. Warden BA, Freels JP, Furuno JP, Mackay J. Pharmacy-managed program for providing education and discharge instructions for patients with heart failure. American Journal of Health-System Pharmacy. 2014 Jan 15;71(2):134–9.

165. Wensaas-2014-Effects of a decision aid and add.pdf [Internet]. [cited 2020 Aug 28]. Available from: https://regroup-

© 2021 Singh K et al. *JAMA Network Open*.

production.s3.amazonaws.com/documents/ReviewReference/149750140/Wensaas-2014-Effects%20of%20a%20decision%20aid%20and%20add.pdf?AWSAccessKeyId=AKIAJBZQODCMKJA4H7DA&Expires=1598615101&Signature=FsSxPP6D9hRSebW2RTXnqHnfAfs%3D

166. Xinlei M, Shui Y. GW25-e2251 Effects of Nurse Case Management on Quality of Life in Patients with Chronic Heart Failure Disease. Journal of the American College of Cardiology. 2014 Oct;64(16):C244.

167. Yingying Z, Dan Z, Jinan L, Yongchun C, Liyan Y. GW25-e4210 Research of self-management intervention on patients with chronic heart failure. J Am Coll Cardiol. 2014 Oct 21;64(16 Supplement):C92.

168. Wang W, Lopez V, Chow A, Chan SW-C, Cheng KKF, He H-G. A randomized controlled trial of the effectiveness of a self-help psychoeducation programme on outcomes of outpatients with coronary heart disease: study protocol. J Adv Nurs. 2014 Dec;70(12):2932–41.

169. Bushnell C, Arnan M, Han S. A New Model for Secondary Prevention of Stroke: Transition Coaching for Stroke. Frontiers in Neurology [Internet]. 2014 [cited 2020 Aug 27];5. Available from: https://cyberleninka.org/article/n/379258

170. Donohue JM, Belnap BH, Men A, He F, Roberts MS, Schulberg HC, et al. Twelve-month cost-effectiveness of telephone-delivered collaborative care for treating depression following CABG surgery: a randomized controlled trial. General Hospital Psychiatry. 2014 Sep;36(5):453–9.

171. Delaney C, Apostolidis B, Bartos S, Robbins R, Young AK. Pilot Testing of the Home Care Education, Assessment, Remote-Monitoring, and Therapeutic Activities Intervention. Home Health Care Management & Practice. 2014 Nov;26(4):205–16.

172. Cameron JI, Naglie G, Gignac MAM, Bayley M, Warner G, Green T, et al. Randomized clinical trial of the timing it right stroke family support program: research protocol. BMC Health Serv Res. 2014 Dec;14(1):18.

173. Tielemans NS, Visser-Meily JMA, Schepers VPM, Post MWM, Wade DT, van Heugten CM. Study Protocol of the Restore4Stroke Self-Management Study: A Multicenter Randomized Controlled Trial in Stroke Patients and Their Partners. International Journal of Stroke. 2014 Aug;9(6):818–23.

174. The BEAT-HF Research Group, Black JT, Romano PS, Sadeghi B, Auerbach AD, Ganiats TG, et al. A remote monitoring and telephone nurse coaching intervention to reduce readmissions among patients with heart failure: study protocol for the Better Effectiveness After Transition - Heart Failure (BEAT-HF) randomized controlled trial. Trials. 2014 Dec;15(1):124.

175. Thomas CL, Man M-S, O’Cathain A, Hollinghurst S, Large S, Edwards L, et al. Effectiveness and cost-effectiveness of a telehealth intervention to support the management of long-term conditions: study protocol for two linked randomized controlled trials. Trials. 2014 Dec;15(1):36.

© 2021 Singh K et al. *JAMA Network Open*.

176. Pandey AK, Choudhry N. TEXT MESSAGE REMINDERS TO ADDRESS MEDICATION NON-ADHERENCE IN POST-MI PATIENTS: A ONE YEAR INTERVENTION STUDY. Canadian Journal of Cardiology. 2014 Oct 1;30(10):S179.

177. Lynggaard V, May O, Beauchamp A, Nielsen CV, Wittrup I. LC-REHAB: randomised trial assessing the effect of a new patient education method - learning and coping strategies – in cardiac rehabilitation. BMC Cardiovasc Disord. 2014 Dec;14(1):186.

178. Cruz_2014_Components of life quality evaluation in heart failure clinic.pdf [Internet]. [cited 2020 Aug 28]. Available from: https://regroup-production.s3.amazonaws.com/documents/ReviewReference/149750989/Cruz_2014_Components%20of%20life%20quality%20evaluation%20in%20heart%20failure%20clinic.pdf?AWSAccessKeyId=AKIAJBZQODCMKJA4H7DA&Expires=1598611719&Signature=km7eB7BWBOIxeATBBcZY7iO4i9Q%3D

179. Arthur K, Collier M, Foreman J, Witt J, Worrell M, Dunlap S, et al. Improving 60 minutes of heart failure education is associated with a 30 day readmission rate reduction. Heart & Lung: The Journal of Cardiopulmonary and Acute Care. 2015 Nov 1;44(6):552.

180. Bekelman DB, Plomondon ME, Carey EP, Sullivan MD, Nelson KM, Hattler B, et al. Primary Results of the Patient-Centered Disease Management (PCDM) for Heart Failure Study: A Randomized Clinical Trial. JAMA Intern Med. 2015 May 1;175(5):725.

181. Byrnes J, Carrington M, Chan Y-K, Pollicino C, Dubrowin N, Stewart S, et al. Cost-Effectiveness of a Home Based Intervention for Secondary Prevention of Readmission with Chronic Heart Disease. PLoS ONE. 2015;10(12):e0144545.

182. Clark AP, McDougall G, Riegel B, Joiner-Rogers G, Innerarity S, Meraviglia M, et al. Health Status and Self-care Outcomes After an Education-Support Intervention for People With Chronic Heart Failure: The Journal of Cardiovascular Nursing. 2015;30:S3–13.

183. Fontes-Carvalho R, Sampaio F, Teixeira M, Gama V, Leite-Moreira AF. The role of a structured exercise training program on cardiac structure and function after acute myocardial infarction: study protocol for a randomized controlled trial. Trials. 2015 Dec;16(1):90.

184. Freedland KE, Carney RM, Rich MW, Steinmeyer BC, Rubin EH. Cognitive Behavior Therapy for Depression and Self-Care in Heart Failure Patients: A Randomized Clinical Trial. JAMA Intern Med. 2015 Nov 1;175(11):1773.

185. Fukuoka Y, Hosomi N, Hyakuta T, Omori T, Ito Y, Uemura J, et al. Baseline Feature of a Randomized Trial Assessing the Effects of Disease Management Programs for the Prevention of Recurrent Ischemic Stroke. Journal of Stroke and Cerebrovascular Diseases. 2015 Mar;24(3):610–7.

186. Granger BB, Ekman I, Hernandez AF, Sawyer T, Bowers MT, DeWald TA, et al. Results of the Chronic Heart Failure Intervention to Improve MEdication Adherence study: A randomized intervention in high-risk patients. American Heart Journal. 2015 Apr;169(4):539–48.

© 2021 Singh K et al. *JAMA Network Open*.

187. Hoffmann T, Ownsworth T, Eames S, Shum D. Evaluation of brief interventions for managing depression and anxiety symptoms during early discharge period after stroke: a pilot randomized controlled trial. Topics in Stroke Rehabilitation. 2015 Apr;22(2):116–26.

188. Kara S, Ntsiea MV. The Effect of a Written and Pictorial Home Exercise Prescription on Adherence for People with Stroke. Hong Kong Journal of Occupational Therapy. 2015 Dec;26(1):33–41.

189. Khonsari S, Subramanian P, Chinna K, Latif LA, Ling LW, Gholami O. Effect of a reminder system using an automated short message service on medication adherence following acute coronary syndrome. European Journal of Cardiovascular Nursing. 2015 Apr;14(2):170–9.

190. Köberich S, Lohrmann C, Mittag O, Dassen T. Effects of a hospital-based education programme on self-care behaviour, care dependency and quality of life in patients with heart failure - a randomised controlled trial. J Clin Nurs. 2015 Jun;24(11–12):1643–55.

191. Li X, Xu S, Zhou L, Li R, Wang J. Home-Based Exercise in Older Adults Recently Discharged From the Hospital for Cardiovascular Disease in China: Randomized Clinical Trial. Nursing Research. 2015;64(4):246–55.

192. Maddison R, Pfaeffli L, Whittaker R, Stewart R, Kerr A, Jiang Y, et al. A mobile phone intervention increases physical activity in people with cardiovascular disease: Results from the HEART randomized controlled trial. Eur J Prev Cardiolog. 2015 Jun;22(6):701–9.

193. Effect of Telemanagement in Patients Discharged to a Nursing Facility After Heart Failure (HF) Hospitalization. August 2015. Journal of Cardiac Failure 21(8):S131. DOI: 10.1016/j.cardfail.2015.06.375 | Request PDF [Internet]. ResearchGate. [cited 2020 Aug 28]. Available from: https://www.researchgate.net/publication/330752564_Effect_of_Telemanagement_in_Patients_Discharged_to_a_Nursing_Facility_After_Heart_Failure_HF_Hospitalization_August_2015_Journal_of_Cardiac_Failure_218S131_DOI_101016jcardfail201506375

194. NYSCHP Residency Research & Practice Forum 2015: Platform Presentation Abstracts. Journal of Pharmacy Practice. 2015 Jun;28(3):298–313.

195. Mohammadpour A, Rahmati Sharghi N, Khosravan S, Alami A, Akhond M. The effect of a supportive educational intervention developed based on the Orem's self-care theory on the self-care ability of patients with myocardial infarction: a randomised controlled trial. J Clin Nurs. 2015 Jun;24(11–12):1686–92.

196. Stroemberg A. Supporting Dyads Affected by Heart Failure - A Randomised Controlled Study Evaluating a Psychoeducational Intervention [Internet]. clinicaltrials.gov; 2015 Mar [cited 2020 Aug 27]. Report No.: NCT02398799. Available from: https://clinicaltrials.gov/ct2/show/NCT02398799

197. Neumann A, Mostardt S, Biermann J, Gelbrich G, Goehler A, Geisler BP, et al. Cost-effectiveness and cost-utility of a structured collaborative disease management in the

© 2021 Singh K et al. *JAMA Network Open*.

Interdisciplinary Network for Heart Failure (INH) study. Clin Res Cardiol. 2015 Apr;104(4):304–9.

198. Olaiya-2015-Shared team approach between nurse.pdf [Internet]. [cited 2020 Aug 28]. Available from: https://regroup-production.s3.amazonaws.com/documents/ReviewReference/149745587/Olaiya-2015-Shared%20team%20approach%20between%20nurse.pdf?AWSAccessKeyId=AKIAJBZQODCMKJA4H7DA&Expires=1598622728&Signature=m%2B7qb0hzhIs2oIW9rlfImmokzJM%3D

199. Ostbring J. EuroHeartCare 2015. European Journal of Cardiovascular Nursing. 2015 Jun;14(1_suppl):S1–120.

200. Pedone C, Rossi FF, Cecere A, Costanzo L, Antonelli Incalzi R. Efficacy of a Physician-Led Multiparametric Telemonitoring System in Very Old Adults with Heart Failure. J Am Geriatr Soc. 2015 Jun;63(6):1175–80.

201. US Department of Veterans Affairs. Enhancing Caregiver Support for Patients With Heart Failure [Internet]. clinicaltrials.gov; 2015 Aug [cited 2020 Aug 27]. Report No.: NCT00555360. Available from: https://clinicaltrials.gov/ct2/show/NCT00555360

202. Piotrowicz E, Stepnowska M, Leszczyńska-Iwanicka K, Piotrowska D, Kowalska M, Tylka J, et al. Quality of life in heart failure patients undergoing home-based telerehabilitation versus outpatient rehabilitation – a randomized controlled study. European Journal of Cardiovascular Nursing. 2015 Jun;14(3):256–63.

203. Ruiz Bustillo-2015-Intensive intervention by s.pdf [Internet]. [cited 2020 Aug 28]. Available from: https://regroup-production.s3.amazonaws.com/documents/ReviewReference/149748749/Ruiz%20Bustillo-2015-Intensive%20intervention%20by%20s.pdf?AWSAccessKeyId=AKIAJBZQODCMKJA4H7DA&Expires=1598624094&Signature=Xui3ECbBTZEpBJKjzwSKa8hKS4A%3D

204. Salavati M, Fallahinia G, Vardanjani AE, Rafiei H, Mousavi S, Torkamani M. Comparison Between Effects of Home Based Cardiac Rehabilitation Programs Versus Usual Care on the Patients' Health Related Quality of Life After Coronary Artery Bypass Graft. GJHS. 2015 Aug 19;8(4):196.

205. Schwalm J-D, Ivers NM, Natarajan MK, Taljaard M, Rao-Melacini P, Witteman HO, et al. Cluster randomized controlled trial of Delayed Educational Reminders for Long-term Medication Adherence in ST-Elevation Myocardial Infarction (DERLA-STEMI). American Heart Journal. 2015 Nov;170(5):903–13.

206. Smith CE, Piamjariyakul U, Dalton KM, Russell C, Wick J, Ellerbeck EF. Nurse-Led Multidisciplinary Heart Failure Group Clinic Appointments: Methods, Materials, and Outcomes Used in the Clinical Trial. The Journal of Cardiovascular Nursing. 2015;30:S25–34.

207. Stojanovic U. EuroHeartCare 2015. European Journal of Cardiovascular Nursing. 2015 Jun;14(1_suppl):S1–120.

© 2021 Singh K et al. *JAMA Network Open*.

208. Trofimov E. Abstracts. European Journal of Heart Failure. 2015;17(S1):5–441.

209. Wong FKY, Yeung SM. Effects of a 4-week transitional care programme for discharged stroke survivors in Hong Kong: a randomised controlled trial. Health Soc Care Community. 2015 Nov;23(6):619–31.

210. Wovkulich M. NYSCHP Residency Research & Practice Forum 2015: Platform Presentation Abstracts. Journal of Pharmacy Practice. 2015 Jun;28(3):298–313.

211. Choudhary, Manish. A Randomized Control Trial to Assess the Effectiveness of a Nurse Led Cardiac Rehabilitation Programme on Health Behaviour and Physiological Parameters Among Post Myocardial Infraction Patients Admitted in Cardiology Unit of AIIMS, New Delhi. Ann Arbor All India Institute of Medical Sciences, New Delhi (India) 2015. 2015;

212. Wagenaar KP, Broekhuizen BDL, Dickstein K, Jaarsma T, Hoes AW, Rutten FH. Effectiveness of an interactive platform, and the ESC/HFA heartfailurematters.org website in patients with heart failure: design of the multicentre randomized e-Vita heart failure trial: Design of the multicentre randomized e-Vita heart failure trial. Eur J Heart Fail. 2015 Dec;17(12):1310–6.

213. Fakhr-Movahedi A, Soleimani M, Ghazvininejad R, Maher MK, Ghorbani R. Effect of Patient-Focused Clinical Pathway on Anxiety, Depression and Satisfaction of Patients With Coronary Artery Disease: A Quasi-Experimental Study [Internet]. Vol. 17, Iranian Red Crescent Medical Journal. Kowsar; 2015 [cited 2020 Aug 27]. Available from: https://sites.kowsarpub.com/ircmj/articles/16739.html

214. Dickson VV, Melkus GD, Dorsen C, Katz S, Riegel B. Improving Heart Failure Self-care Through a Community-Based Skill-Building Intervention: A Study Protocol. The Journal of Cardiovascular Nursing. 2015;30:S14–24.

215. Masterson Creber R, Patey M, Dickson VV, DeCesaris M, Riegel B. Motivational Interviewing Tailored Interventions for Heart Failure (MITI-HF): Study design and methods. Contemporary Clinical Trials. 2015 Mar;41:62–8.

216. Deek-2015-Family focused approach to improve h.pdf [Internet]. [cited 2020 Aug 31]. Available from: https://regroup-production.s3.amazonaws.com/documents/ReviewReference/149749260/Deek-2015-Family%20focused%20approach%20to%20improve%20h.pdf?AWSAccessKeyId=AKIAJBZQODCMKJA4H7DA&Expires=1598858267&Signature=p4njVrZCs%2B0bQIRqecHVXfLf1Fc%3D

217. Lord AS, Carman HM, Roberts ET, Torrico V, Goldmann E, Ishida K, et al. Discharge educational strategies for reduction of vascular events (DESERVE): design and methods. International Journal of Stroke. 2015 Oct;10(SA100):151–4.

218. Denis Komkov-2015-Effect of education and tele.pdf [Internet]. [cited 2020 Aug 28]. Available from: https://regroup-production.s3.amazonaws.com/documents/ReviewReference/149749950/Denis%20Komkov-2015-

© 2021 Singh K et al. *JAMA Network Open*.

Effect%20of%20education%20and%20tele.pdf?AWSAccessKeyId=AKIAJBZQODCMKJA4H7DA&Expires=1598618076&Signature=YIVx2WuByF%2BSHQQtnajjmr6zgjw%3D

219. Gencer-2015-Effect of a multi-dimensional seco.pdf [Internet]. [cited 2020 Aug 28]. Available from: https://regroup-production.s3.amazonaws.com/documents/ReviewReference/149750457/Gencer-2015-Effect%20of%20a%20multi-dimensional%20seco.pdf?AWSAccessKeyId=AKIAJBZQODCMKJA4H7DA&Expires=1598618603&Signature=u2nWD7gRdCPsDg%2FDtHC%2FGlKv74E%3D

220. Berna-2016-Investigation of the effect of tele.pdf [Internet]. [cited 2020 Aug 29]. Available from: https://regroup-production.s3.amazonaws.com/documents/ReviewReference/149748191/Berna-2016-Investigation%20of%20the%20effect%20of%20tele.pdf?AWSAccessKeyId=AKIAJBZQODCMKJA4H7DA&Expires=1598692873&Signature=j4Lqus27wKo38WiRUO8Gk9PVZbo%3D

221. Bertilsson A-S, Eriksson G, Ekstam L, Tham K, Andersson M, von Koch L, et al. A cluster randomized controlled trial of a client-centred, activities of daily living intervention for people with stroke: one year follow-up of caregivers. Clin Rehabil. 2016 Aug;30(8):765–75.

222. Abstracts for the 10th World Stroke Congress, 2016, 2016 [Internet]. [cited 2020 Aug 29]. Available from: https://journals.sagepub.com/doi/10.1177/1747493016670567

223. Nahlén Bose C, Persson H, Björling G, Ljunggren G, Elfström ML, Saboonchi F. Evaluation of a Coping Effectiveness Training intervention in patients with chronic heart failure – a randomized controlled trial. European Journal of Cardiovascular Nursing. 2016 Dec;15(7):537–48.

224. Calugi S, Taricco M, Rucci P, Fugazzaro S, Stuart M, Dallolio L, et al. Effectiveness of adaptive physical activity combined with therapeutic patient education in stroke survivors at twelve months: a non-randomized parallel group study. Eur J Phys Rehabil Med. 2016 Feb;52(1):72–80.

225. Chang Y-L, Chiou A-F, Cheng S-M, Lin K-C. Tailored educational supportive care programme on sleep quality and psychological distress in patients with heart failure: A randomised controlled trial. International Journal of Nursing Studies. 2016 Sep;61:219–29.

226. Chen J-T, Lin T-H, Voon W-C, Lai W-T, Huang M-H, Sheu S-H, et al. Beneficial effects of home-based cardiac rehabilitation on metabolic profiles in coronary heart-disease patients. The Kaohsiung Journal of Medical Sciences. 2016;32(5):267–75.

227. Comín J. Telemonitoring and Teleintervention of Heart Failure and Decrease of Non-fatal Events. [Internet]. clinicaltrials.gov; 2016 Feb [cited 2020 Aug 27]. Report No.: NCT01495078. Available from: https://clinicaltrials.gov/ct2/show/NCT01495078

228. Barutcu CD, Mert H. Effect of Support Group Intervention Applied to the Caregivers of Individuals With Heart Failure on Caregiver Outcomes: Holistic Nursing Practice. 2016;30(5):272–82.

© 2021 Singh K et al. *JAMA Network Open*.

229. Deri Armstrong C, Taljaard M, Hogg W, Mark AE, Liddy C. Practice facilitation for improving cardiovascular care: secondary evaluation of a stepped wedge cluster randomized controlled trial using population-based administrative data. Trials. 2016 Dec;17(1):434.

230. Elahi, N. Abstracts. European Journal of Heart Failure. 2016;18(S1):8–521.

231. Fang R, Li X. Electronic messaging support service programs improve adherence to lipid-lowering therapy among outpatients with coronary artery disease: an exploratory randomised control study. J Clin Nurs. 2016 Mar;25(5–6):664–71.

232. Fors A, Taft C, Ulin K, Ekman I. Person-centred care improves self-efficacy to control symptoms after acute coronary syndrome: a randomized controlled trial. European Journal of Cardiovascular Nursing. 2016 Apr;15(2):186–94.

233. Jørstad HT, Minneboo M, Helmes HJM, Fagel ND, Scholte op Reimer WJ, Tijssen JGP, et al. Effects of a nurse-coordinated prevention programme on health-related quality of life and depression in patients with an acute coronary syndrome: results from the RESPONSE randomised controlled trial. BMC Cardiovasc Disord. 2016 Dec;16(1):144.

234. Kidholm K, Rasmussen MK, Andreasen JJ, Hansen J, Nielsen G, Spindler H, et al. Cost-Utility Analysis of a Cardiac Telerehabilitation Program: The Teledialog Project. Telemedicine and e-Health. 2016 Jul;22(7):553–63.

235. Koh KWL, Wang W, Richards AM, Chan MY, Cheng KKF. Effectiveness of advanced practice nurse-led telehealth on readmissions and health-related outcomes among patients with post-acute myocardial infarction: ALTRA Study Protocol. J Adv Nurs. 2016 Jun;72(6):1357–67.

236. Kollia-2016-Clinical outcomes in patients with.pdf [Internet]. [cited 2020 Aug 29]. Available from: https://regroup-production.s3.amazonaws.com/documents/ReviewReference/149751104/Kollia-2016-Clinical%20outcomes%20in%20patients%20with.pdf?AWSAccessKeyId=AKIAJBZQODCMKJA4H7DA&Expires=1598701673&Signature=gLIkBHB6RlDVS%2FmxdvG0GPbnEZQ%3D

237. Larsen P, Pedersen PU. The effectiveness of individual rehabilitation on health status in patients with heart failure: A quasi-experimental study: Health status and heart failure. International Journal of Nursing Practice. 2016 Feb;22(1):15–21.

238. Masterson Creber R, Patey M, Lee CS, Kuan A, Jurgens C, Riegel B. Motivational interviewing to improve self-care for patients with chronic heart failure: MITI-HF randomized controlled trial. Patient Education and Counseling. 2016 Feb;99(2):256–64.

239. McLennon SM, Hancock RD, Redelman K, Scarton LJ, Riley E, Sweeney B, et al. Comparing treatment fidelity between study arms of a randomized controlled clinical trial for stroke family caregivers. Clin Rehabil. 2016 May;30(5):495–507.

240. Consorci Sanitari del Maresme. Randomized Trial of CONTECI Program: Pilot Study [Internet]. clinicaltrials.gov; 2016 May [cited 2020 Aug 27]. Report No.: NCT02762916. Available from: https://clinicaltrials.gov/ct2/show/NCT02762916

© 2021 Singh K et al. *JAMA Network Open*.

241. Prasun MA, Short MA, Maynard JK, Roth J, Bell J, Nallamothu T. Outcomes of an Inter-Professional Intervention on Medication Adherence Among Heart Failure Patients: A Pilot Study. Journal of Cardiac Failure. 2016 Aug 1;22(8):S81.

242. Bernocchi-2016-A multidisciplinary telehealth.pdf [Internet]. [cited 2020 Aug 29]. Available from: https://regroup-production.s3.amazonaws.com/documents/ReviewReference/149747911/Bernocchi-2016-A%20multidisciplinary%20telehealth.pdf?AWSAccessKeyId=AKIAJBZQODCMKJA4H7DA&Expires=1598703505&Signature=Re%2B37EPTy3XFXd7nA9%2Fu7Pit2UI%3D

243. Shelley-2016-Testing the use of practice facil.pdf [Internet]. [cited 2020 Aug 29]. Available from: https://regroup-production.s3.amazonaws.com/documents/ReviewReference/149744878/Shelley-2016-Testing%20the%20use%20of%20practice%20facil.pdf?AWSAccessKeyId=AKIAJBZQODCMKJA4H7DA&Expires=1598703966&Signature=G5uGPNHxA7gXCSUZKGNlCeub6mA%3D

244. Shim JL, Hwang SY. Development and Effects of a Heart Health Diary for Self-Care Enhancement of Patients with Heart Failure. J Korean Acad Nurs. 2016;46(6):881.

245. Central Norway Regional Health Authority. Self-management Behaviour After an Individual Nurse-led Counselling Programme for Patients Early Discharged After Myocardial Infarction: A Randomised Controlled Trial. [Internet]. clinicaltrials.gov; 2016 Nov [cited 2020 Aug 27]. Report No.: NCT02640274. Available from: https://clinicaltrials.gov/ct2/show/NCT02640274

246. Vahedian-Azimi A, Miller AC, Hajiesmaieli M, Kangasniemi M, Alhani F, Jelvehmoghaddam H, et al. Cardiac rehabilitation using the Family-Centered Empowerment Model versus home-based cardiac rehabilitation in patients with myocardial infarction: a randomised controlled trial. Open Heart. 2016 Apr;3(1):e000349.

247. Wan L-H, Zhang X-P, Mo M-M, Xiong X-N, Ou C-L, You L-M, et al. Effectiveness of Goal-Setting Telephone Follow-Up on Health Behaviors of Patients with Ischemic Stroke: A Randomized Controlled Trial. Journal of Stroke and Cerebrovascular Diseases. 2016 Sep;25(9):2259–70.

248. Wang T-C, Huang J-L, Ho W-C, Chiou A-F. Effects of a supportive educational nursing care programme on fatigue and quality of life in patients with heart failure: a randomised controlled trial. European Journal of Cardiovascular Nursing. 2016 Apr;15(2):157–67.

249. Young L, Hertzog M, Barnason S. Effects of a home-based activation intervention on self-management adherence and readmission in rural heart failure patients: the PATCH randomized controlled trial. BMC Cardiovasc Disord. 2016 Dec;16(1):176.

250. Snaterse-Zuidam-2016-Nurse-coordinated care im.pdf [Internet]. [cited 2020 Aug 29]. Available from: https://regroup-production.s3.amazonaws.com/documents/ReviewReference/149747687/Snaterse-Zuidam-2016-Nurse-coordinated%20care%20im.pdf?AWSAccessKeyId=AKIAJBZQODCMKJA4H7DA&Expires=1598704537&Signature=amSS%2BE47be05AcQ7dO%2FyEEKh2ts%3D

© 2021 Singh K et al. *JAMA Network Open*.

251. Sharma KK, Gupta R, Mathur M, Natani V, Lodha S, Roy S, et al. Non-physician health workers for improving adherence to medications and healthy lifestyle following acute coronary syndrome: 24-month follow-up study. Indian Heart Journal. 2016 Nov 1;68(6):832–40.

252. Wan L-H, You L-M, Chen S-X, Zhang X-P, Mo M-M, Zhang Y-M, et al. The effectiveness of a comprehensive reminder system in the secondary prevention of hypertensive ischaemic stroke: randomized controlled trial protocol. J Adv Nurs. 2016 Dec;72(12):3195–206.

253. Late-Breaking Abstracts in Resuscitation Science From the Resuscitation Science Symposium 2016. Circulation. 2016 Dec 20;134(25):e716–20.

254. Yan LL, Chen S, Zhou B, Zhang J, Xie B, Luo R, et al. A randomized controlled trial on rehabilitation through caregiver-delivered nurse-organized service programs for disabled stroke patients in rural china (the RECOVER trial): design and rationale. International Journal of Stroke. 2016 Oct;11(7):823–30.

255. Winstein CJ, Wolf SL, Dromerick AW, Lane CJ, Nelsen MA, Lewthwaite R, et al. Effect of a Task-Oriented Rehabilitation Program on Upper Extremity Recovery Following Motor Stroke. JAMA. 2016 Feb 9;315(6):571–81.

256. Kamal AK, Khoja A, Usmani B, Muqeet A, Zaidi F, Ahmed M, et al. Translating knowledge for action against stroke – using 5-minute videos for stroke survivors and caregivers to improve post-stroke outcomes: study protocol for a randomized controlled trial (Movies4Stroke). Trials. 2016 Dec;17(1):52.

257. Cichosz SL, Ehlers LH, Hejlesen O. Health effectiveness and cost-effectiveness of telehealthcare for heart failure: study protocol for a randomized controlled trial. Trials. 2016 Dec;17(1):590.

258. PhD SMH MD. Determining the Impact of a Medication Delivery Unit on Medication Adherence Immediately Following Hospitalization of Adults With Common Cardiac Conditions [Internet]. clinicaltrials.gov; 2016 Aug [cited 2020 Aug 25]. Report No.: NCT01600677. Available from: https://clinicaltrials.gov/ct2/show/NCT01600677

259. Olaiya MT, Cadilhac DA, Kim J, Ung D, Nelson MR, Srikanth VK, et al. Nurse-Led Intervention to Improve Knowledge of Medications in Survivors of Stroke or Transient Ischemic Attack: A Cluster Randomized Controlled Trial. Front Neurol [Internet]. 2016 Nov 18 [cited 2020 Sep 1];7. Available from: https://www.ncbi.nlm.nih.gov/pmc/articles/PMC5114293/

260. Cavusoglu Y. Post-discharge heart failure monitoring program in Turkey: Hit-PoinT. Anatol J Cardiol [Internet]. 2016 [cited 2020 Aug 31]; Available from: https://www.journalagent.com/anatoljcardiol/pdfs/AJC_17_2_107_112.pdf

261. Cao X, Wang X-H, Wong EM, Chow CK, Chair SY. Type D personality negatively associated with self-care in Chinese heart failure patients. J Geriatr Cardiol. 2016 Jul;13(5):401–7.

© 2021 Singh K et al. *JAMA Network Open*.

262. Lo SHS, Chang AM, Chau JPC. Study protocol: a randomised controlled trial of a nurse-led community-based self-management programme for improving recovery among community-residing stroke survivors. BMC Health Serv Res. 2016 Dec;16(1):387.

263. Demers C. A Randomized Controlled Trial of Enhanced Caregiver Support Versus Usual Care for Managing Older Heart Failure Patients at Hospital Discharge [Internet]. clinicaltrials.gov; 2016 Dec [cited 2020 Aug 27]. Report No.: NCT01886534. Available from: https://clinicaltrials.gov/ct2/show/NCT01886534

264. Ambrosy AP, Cerbin LP, DeVore AD, Greene SJ, Kraus WE, O'Connor CM, et al. Aerobic exercise training and general health status in ambulatory heart failure patients with a reduced ejection fraction—Findings from the Heart Failure and A Controlled Trial Investigating Outcomes of Exercise Training (HF-ACTION)trial. American Heart Journal. 2017 Apr;186:130–8.

265. Berndt N, Lechner L, Mudde A, De Vries H, Bolman C. Feasibility and acceptability of a telephone- and face-to-face-delivered counseling intervention for smoking cessation in Dutch patients with coronary heart disease. Res Nurs Health. 2017 Oct;40(5):444–58.

266. Bertelsen JB, Refsgaard J, Kanstrup H, Johnsen SP, Qvist I, Christensen B, et al. Cardiac rehabilitation after acute coronary syndrome comparing adherence and risk factor modification in a community-based shared care model versus hospital-based care in a randomised controlled trial with 12 months of follow-up. European Journal of Cardiovascular Nursing. 2017 Apr;16(4):334–43.

267. Bikmoradi A, Masmouei B, Ghomeisi M, Roshanaei G, Masiello I. Impact of telephone counseling on the quality of life of patients discharged after coronary artery bypass grafts. Patient Education and Counseling. 2017 Dec;100(12):2290–6.

268. Brouwers RWM, Kraal JJ, Traa SCJ, Spee RF, Oostveen LMLC, Kemps HMC. Effects of cardiac telerehabilitation in patients with coronary artery disease using a personalised patient-centred web application: protocol for the SmartCare-CAD randomised controlled trial. BMC Cardiovasc Disord. 2017 Dec;17(1):46.

269. Cao X-Y, Tian L, Chen L, Jiang X-L. Effects of a hospital-community partnership transitional program in patients with coronary heart disease in Chengdu, China: A randomized controlled trial: Coronary heart disease transitional care. Jpn J Nurs Sci. 2017 Oct;14(4):320–31.

270. Claes J, Buys R, Woods C, Briggs A, Geue C, Aitken M, et al. PATHway I: design and rationale for the investigation of the feasibility, clinical effectiveness and cost-effectiveness of a technology-enabled cardiac rehabilitation platform. BMJ Open. 2017 Jun;7(6):e016781.

271. APhA2017 abstracts of contributed papers. Journal of the American Pharmacists Association. 2017 May 1;57(3):e1–142.

272. Ding H, Jayasena R, Maiorana A, Dowling A, Chen SH, Karunanithi M, et al. Innovative Telemonitoring Enhanced Care Programme for Chronic Heart Failure (ITEC-CHF) to improve

guideline compliance and collaborative care: protocol of a multicentre randomised controlled trial. Open Access. :8.

273. Emmerson KB, Harding KE, Taylor NF. Home exercise programmes supported by video and automated reminders compared with standard paper-based home exercise programmes in patients with stroke: a randomized controlled trial. Clin Rehabil. 2017 Aug;31(8):1068–77.

274. Bravo-Escobar R, González-Represas A, Gómez-González AM, Montiel-Trujillo A, Aguilar-Jimenez R, Carrasco-Ruíz R, et al. Effectiveness and safety of a home-based cardiac rehabilitation programme of mixed surveillance in patients with ischemic heart disease at moderate cardiovascular risk: A randomised, controlled clinical trial. BMC Cardiovasc Disord. 2017 Dec;17(1):66.

275. Figueiras MJ, Maroco J, Monteiro R, Caeiro R, Dias Neto D. Randomized controlled trial of an intervention to change cardiac misconceptions in myocardial infarction patients. Psychology, Health & Medicine. 2017 Mar 16;22(3):255–65.

276. VA Office of Research and Development. Controlling Hypertension Outcomes by Improved Communication & Engagement (CHOICE) [Internet]. clinicaltrials.gov; 2017 Jun [cited 2020 Aug 27]. Report No.: NCT01134887. Available from: https://clinicaltrials.gov/ct2/show/NCT01134887

277. Gao-2017-Different interventions for post-isch.pdf [Internet]. [cited 2020 Aug 31]. Available from: https://regroup-production.s3.amazonaws.com/documents/ReviewReference/149750275/Gao-2017-Different%20interventions%20for%20post-isch.pdf?AWSAccessKeyId=AKIAJBZQODCMKJA4H7DA&Expires=1598859652&Signature=rAK48yMLWASff9%2BC9QYUXwSTyIM%3D

278. Gordon Linda, Malecky Amanda, Althouse Andrew, Ansani Nicole. Abstract 225: A Depression Quality Program and Cardiovascular Disease: Assessing the Impact on Care. Circulation: Cardiovascular Quality and Outcomes. 2017 Mar 1;10(suppl_3):A225–A225.

279. Hill VA, Vickrey BG, Cheng EM, Valle NP, Ayala-Rivera M, Moreno L, et al. A Pilot Trial of a Lifestyle Intervention for Stroke Survivors: Design of Healthy Eating and Lifestyle after Stroke (HEALS). Journal of Stroke and Cerebrovascular Diseases. 2017 Dec;26(12):2806–13.

280. Hoover C, Plamann J, Beckel J. Outcomes of an Interdisciplinary Transitional Care Quality Improvement Project on Self-Management and Health Care Use in Patients With Heart Failure. J Gerontol Nurs. 2017 Jan 1;43(1):23–31.

281. Hwang R, Bruning J, Morris NR, Mandrusiak A, Russell T. Home-based telerehabilitation is not inferior to a centre-based program in patients with chronic heart failure: a randomised trial. Journal of Physiotherapy. 2017 Apr;63(2):101–7.

282. Liu H, Mohammed A, Felix C, Gandhi DB, Verma S, Tugnawat D, et al. Process evaluation of a randomised controlled trial of a post stroke family-led rehabilitation intervention in India. Journal of the Neurological Sciences. 2017 Oct;381:884.

© 2021 Singh K et al. *JAMA Network Open*.

283. Mansfield A, Brooks D, Tang A, Taylor D, Inness EL, Kiss A, et al. Promoting Optimal Physical Exercise for Life (PROPEL): aerobic exercise and self-management early after stroke to increase daily physical activity—study protocol for a stepped-wedge randomised trial. BMJ Open. 2017 Jun;7(6):e015843.

284. Mathews Robin, Shrader Peter, Demyaneko Vladimir, Miller Vincent, Webb Laura, Washam Jeffrey W, et al. Abstract 247: A Randomized Controlled Study of a Platform to Maximize Patient Knowledge of Health Goals After Acute Myocardial Infarction. Circulation: Cardiovascular Quality and Outcomes. 2017 Mar 1;10(suppl_3):A247–A247.

285. Meslot C, Gauchet A, Hagger MS, Chatzisarantis N, Lehmann A, Allenet B. A Randomised Controlled Trial to Test the Effectiveness of Planning Strategies to Improve Medication Adherence in Patients with Cardiovascular Disease. Appl Psychol Health Well-Being. 2017 Mar;9(1):106–29.

286. Minneboo-2017-Community-Based Lifestyle Interv.pdf [Internet]. [cited 2020 Aug 31]. Available from: https://regroup-production.s3.amazonaws.com/documents/ReviewReference/149751529/Minneboo-2017-Community-Based%20Lifestyle%20Interv.pdf?AWSAccessKeyId=AKIAJBZQODCMKJA4H7DA&Expires=1598862294&Signature=%2BrDy3iAMsbVWnxBx5U2y3fj27R4%3D

287. Pekmezaris R. Telehealth Self-Management Program in Older Adults Living With Heart Failure in Health Disparity Communities [Internet]. clinicaltrials.gov; 2017 Oct [cited 2020 Aug 27]. Report No.: NCT02196922. Available from: https://clinicaltrials.gov/ct2/show/NCT02196922

288. Oksman E, Linna M, Hörhammer I, Lammintakanen J, Talja M. Cost-effectiveness analysis for a tele-based health coaching program for chronic disease in primary care. BMC Health Serv Res. 2017 Dec;17(1):138.

289. Sezgin D, Mert H, Özpelit E, Akdeniz B. The effect on patient outcomes of a nursing care and follow-up program for patients with heart failure: A randomized controlled trial. International Journal of Nursing Studies. 2017 May;70:17–26.

290. Souter C, Kinnear A, Kinnear M, Mead G. A pilot study to assess the practicality, acceptability and feasibility of a randomised controlled trial to evaluate the impact of a pharmacist complex intervention on patients with stroke in their own homes. Eur J Hosp Pharm. 2017 Mar;24(2):101–6.

291. Towfighi A, Cheng EM, Ayala-Rivera M, McCreath H, Sanossian N, Dutta T, et al. Randomized controlled trial of a coordinated care intervention to improve risk factor control after stroke or transient ischemic attack in the safety net: Secondary stroke prevention by Uniting Community and Chronic care model teams Early to End Disparities (SUCCEED). BMC Neurol. 2017 Dec;17(1):24.

292. Volpp KG, Troxel AB, Mehta SJ, Norton L, Zhu J, Lim R, et al. Effect of Electronic Reminders, Financial Incentives, and Social Support on Outcomes After Myocardial Infarction: The HeartStrong Randomized Clinical Trial. JAMA Intern Med. 2017 Aug 1;177(8):1093.

© 2021 Singh K et al. *JAMA Network Open*.

293. Wahab KW, Owolabi M, Akinyemi R, Jenkins C, Arulogun O, Akpa O, et al. Short-term pilot feasibility study of a nurse-led intervention to improve blood pressure control after stroke in Nigeria. Journal of the Neurological Sciences. 2017 Jun;377:116–20.

294. Wu C-J, Atherton JJ, MacIsaac RJ, Courtney M, Chang AM, Thompson DR, et al. Effectiveness of the cardiac-diabetes transcare program: protocol for a randomised controlled trial. BMC Health Serv Res. 2017 Dec;17(1):109.

295. Laal N, Shekarriz-Foumani R, Khodaie F, Abadi A, Heidarnia MA. Effects of patient education and follow up after discharge on hospital readmission in heart failure patients. Research in Medicine. 2017 Apr 10;41(1):24–30.

296. Treskes RW, van Winden LA, van Keulen N, Atsma DE, van der Velde ET, van den Akker-van Marle E, et al. Using Smart Technology to Improve Outcomes in Myocardial Infarction Patients: Rationale and Design of a Protocol for a Randomized Controlled Trial, The Box. JMIR Res Protoc. 2017 Sep 22;6(9):e186.

297. Maru S, Byrnes JM, Carrington MJ, Stewart S, Scuffham PA. Long-term cost-effectiveness of home versus clinic-based management of chronic heart failure: the WHICH? study. Journal of Medical Economics. 2017 Apr 3;20(4):318–27.

298. Srisuk N, Cameron J, Ski CF, Thompson DR. Randomized controlled trial of family-based education for patients with heart failure and their carers. J Adv Nurs. 2017 Apr;73(4):857–70.

299. Appalasamy JR, Tha KK, Quek KF, Ramaiah SS, Joseph JP, Md Zain AZ. The effectiveness of culturally tailored video narratives on medication understanding and use self-efficacy among stroke patients: A randomized controlled trial study protocol. Medicine. 2018 Jun;97(22):e10876.

300. Athar MW, Record JD, Martire C, Hellmann DB, Ziegelstein RC. The Effect of a Personalized Approach to Patient Education on Heart Failure Self-Management. JPM. 2018 Nov 27;8(4):39.

301. Avila A, Claes J, Goetschalckx K, Buys R, Azzawi M, Vanhees L, et al. Home-Based Rehabilitation With Telemonitoring Guidance for Patients With Coronary Artery Disease (Short-Term Results of the TRiCH Study): Randomized Controlled Trial. Journal of Medical Internet Research. 2018;20(6):e225.

302. Batalik L, Dosbaba F, Hartman M, Batalikova K, Spinar J. Rationale and design of randomized controlled trial protocol of cardiovascular rehabilitation based on the use of telemedicine technology in the Czech Republic (CR-GPS): Medicine. 2018 Sep;97(37):e12385.

303. Bistola, V. Abstracts. European Journal of Heart Failure. 2018;20(S1):5–638.

304. Caramlau, I. Abstracts. European Journal of Heart Failure. 2018;20(S1):5–638.

305. Chen L, Chen Y, Chen X, Shen X, Wang Q, Sun C. Longitudinal Study of Effectiveness of a Patient-Centered Self-Management Empowerment Intervention During Predischarge

© 2021 Singh K et al. *JAMA Network Open*.

Planning on Stroke Survivors. Worldviews on Evidence-Based Nursing. 2018 Jun;15(3):197–205.

306. Chen Y-W, Wang C-Y, Lai Y-H, Liao Y-C, Wen Y-K, Chang S-T, et al. Home-based cardiac rehabilitation improves quality of life, aerobic capacity, and readmission rates in patients with chronic heart failure: Medicine. 2018 Jan;97(4):e9629.

307. Cheng HY, Chair SY, Chau JPC. Effectiveness of a strength-oriented psychoeducation on caregiving competence, problem-solving abilities, psychosocial outcomes and physical health among family caregiver of stroke survivors: A randomised controlled trial. International Journal of Nursing Studies. 2018 Nov;87:84–93.

308. Clements Linda, Moser Debra K, Lennie Terry A, Frazier Susan K, Chung Misook L. Abstract 15195: Improvement of Heart Failure Self-Care and Patient Readmission With Caregiver Education. Circulation. 2018 Nov 6;138(Suppl_1):A15195–A15195.

309. Coombes JA, Rowett D, Whitty JA, Cottrell WN. Use of a patient-centred educational exchange (PCEE) to improve patient's self-management of medicines after a stroke: a randomised controlled trial study protocol. BMJ Open. 2018 Aug;8(8):e022225.

310. Damush Teresa M, Mackey Jason, Saha Chandan, Slaven James, Myers Laura, Lincoln Flossy, et al. Abstract TMP46: Stroke Self-management Effectiveness Trial. Stroke. 49(Suppl_1):ATMP46–ATMP46.

311. Daniels Brock, Del Toro Cristina, Zelenetz Michael, Gogia Kriti, Hafeez Baria, Greenwald Peter, et al. Abstract 14280: Can Telemedicine-Assisted Paramedicine Reduce Emergency Department Visits and 30-Day Readmissions Among High-Risk Heart Failure Patients? a Pilot Study. Circulation. 2018 Nov 6;138(Suppl_1):A14280–A14280.

312. Compliance of the Web-based Distance Training and Consultancy on Individual's Treatment having Suffered Myocardial Infarction and its Effects on Well-being. J Coll Physicians Surg Pak. 2018 Dec 1;28(12):953–9.

313. Cadilhac DA, Busingye D, Li JC, Andrew NE, Kilkenny MF, Thrift AG, et al. Development of an electronic health message system to support recovery after stroke: Inspiring Virtual Enabled Resources following Vascular Events (iVERVE). Patient Prefer Adherence. 2018 Jul 11;12:1213–24.

314. Dorje T, Zhao G, Scheer A, Tsokey L, Wang J, Chen Y, et al. SMARTphone and social media-based Cardiac Rehabilitation and Secondary Prevention (SMART-CR/SP) for patients with coronary heart disease in China: a randomised controlled trial protocol. BMJ Open. 2018 Jun;8(6):e021908.

315. Duan YP, Liang W, Guo L, Wienert J, Si GY, Lippke S. Evaluation of a Web-Based Intervention for Multiple Health Behavior Changes in Patients With Coronary Heart Disease in Home-Based Rehabilitation: Pilot Randomized Controlled Trial. J Med Internet Res [Internet]. 2018 Nov 19 [cited 2020 Aug 31];20(11). Available from: https://www.ncbi.nlm.nih.gov/pmc/articles/PMC6277829/

© 2021 Singh K et al. *JAMA Network Open*.

316. Bushnell CD, Duncan PW, Lycan SL, Condon CN, Pastva AM, Lutz BJ, et al. A Person-Centered Approach to Poststroke Care: The COMprehensive Post-Acute Stroke Services Model: The COMPASS Care Model. J Am Geriatr Soc. 2018 May;66(5):1025–30.

317. Ezeugwu VE, Manns PJ. The Feasibility and Longitudinal Effects of a Home-Based Sedentary Behavior Change Intervention After Stroke. Archives of Physical Medicine and Rehabilitation. 2018 Dec;99(12):2540–7.

318. Graven LJ, Gordon G, Keltner JG, Abbott L, Bahorski J. Efficacy of a social support and problem-solving intervention on heart failure self-care: A pilot study. Patient Education and Counseling. 2018 Feb;101(2):266–75.

319. Guerra EDPH. Decision Making for the Management for the Symptoms in Adults of Heart Failure: Randomized Clinical Trial [Internet]. clinicaltrials.gov; 2018 Jun [cited 2020 Aug 27]. Report No.: NCT03549169. Available from: https://clinicaltrials.gov/ct2/show/NCT03549169

320. Herring LY, Dallosso H, Chatterjee S, Bodicoat D, Schreder S, Khunti K, et al. Physical Activity after Cardiac EventS (PACES) – a group education programme with subsequent text-message support designed to increase physical activity in individuals with diagnosed coronary heart disease: study protocol for a randomised controlled trial. Trials. 2018 Dec;19(1):537.

321. European Stroke Organisation Conference: Abstracts. European Stroke Journal. 2018 May;3(1_suppl):3–204.

322. Hornnes N. Prevention After Stroke - a Nurse-led Physician-Supervised Model. A Feasibility Study [Internet]. clinicaltrials.gov; 2018 Dec [cited 2020 Aug 27]. Report No.: NCT03782857. Available from: https://clinicaltrials.gov/ct2/show/NCT03782857

323. Bhattacharjee. Poster Presentations. International Journal of Stroke. 2014 Jul;9(1_suppl):27–48.

324. Liu C-Y, Du J-Z, Rao C-F, Zhang H, Liu H-N, Zhao Y, et al. Quality Measurement and Improvement Study of Surgical Coronary Revascularization: Medication Adherence (MISSION-2). Chinese Medical Journal. 2018 Jun;131(12):1480–9.

325. Manap NA, Sharoni SKA, Rahman PA, Majid HAMA. Effect of an Education Programme on Cardiovascular Health Index among Patients with Myocardial Infarction: A Preliminary Study. Malays J Med Sci. 2018 Mar;25(2):105–15.

326. Maru S, Byrnes J, Carrington MJ, Chan Y-K, Stewart S, Scuffham PA. Economic evaluation of a nurse-led home and clinic-based secondary prevention programme to prevent progressive cardiac dysfunction in high-risk individuals: The Nurse-led Intervention for Less Chronic Heart Failure (NIL-CHF) randomized controlled study. European Journal of Cardiovascular Nursing. 2018 Jun;17(5):439–45.

327. on the behalf of Strokavenir network, Mendyk A-M, Duhamel A, Bejot Y, Leys D, Derex L, et al. Controlled Education of patients after Stroke (CEOPS)- nurse-led multimodal and long-term interventional program involving a patient's caregiver to optimize secondary prevention of stroke: study protocol for a randomized controlled trial. Trials. 2018 Dec;19(1):137.
© 2021 Singh K et al. *JAMA Network Open*.

328. Mohammadi N, Aghayousefi A, Nikrahan GR, Adams CN, Alipour A, Sadeghi M, et al. A randomized trial of an optimism training intervention in patients with heart disease. General Hospital Psychiatry. 2018 Mar;51:46–53.

329. Moon MK, Yim J, Jeon MY. The Effect of a Telephone-Based Self-management Program Led by Nurses on Self-care Behavior, Biological Index for Cardiac Function, and Depression in Ambulatory Heart Failure Patients. Asian Nursing Research. 2018 Dec;12(4):251–7.

330. Medical University of South Carolina. Tailored Hospital-based Risk Reduction to Impede Vascular Events After Stroke [Internet]. clinicaltrials.gov; 2018 May [cited 2020 Aug 27]. Report No.: NCT01900756. Available from: https://clinicaltrials.gov/ct2/show/NCT01900756

331. Nguyen T, Nguyen TH, Nguyen PT, Tran HT, Nguyen NV, Nguyen HQ, et al. Pharmacist-Led Intervention to Enhance Medication Adherence in Patients With Acute Coronary Syndrome in Vietnam: A Randomized Controlled Trial. Front Pharmacol [Internet]. 2018 Jun 21 [cited 2020 Aug 31];9. Available from: https://www.ncbi.nlm.nih.gov/pmc/articles/PMC6021484/

332. Norman J, Fu M, Ekman I, Björck L, Falk K. Effects of a mindfulness-based intervention on symptoms and signs in chronic heart failure: A feasibility study. European Journal of Cardiovascular Nursing. 2018 Jan;17(1):54–65.

333. Østergaard B, Mahrer-Imhof R, Wagner L, Barington T, Videbæk L, Lauridsen J. Effect of family nursing therapeutic conversations on health-related quality of life, self-care and depression among outpatients with heart failure: A randomized multi-centre trial. Patient Education and Counseling. 2018 Aug;101(8):1385–93.

334. Schmidt C, Öner A, Mann M, Krockenberger K, Abbondanzieri M, Brandewiede B, et al. A novel integrated care concept (NICC) versus standard care in the treatment of chronic cardiovascular diseases: protocol for the randomized controlled trial CardioCare MV. Trials. 2018 Dec;19(1):120.

335. Soleimani F, Anbohi SZ, Esmaeili R, Pourhoseingholi MA, Borhani F. Person-centered Nursing to Improve Treatment Regimen Adherence in Patients with Myocardial Infarction. JCDR [Internet]. 2018 [cited 2020 Aug 31]; Available from: http://jcdr.net/article_fulltext.asp?issn=0973-709x&year=2018&volume=12&issue=1&page=LC01&issn=0973-709x&id=11131

336. Thames_2018_Incorporating Technology to Decrease Heart Failure Readmission Rates.pdf [Internet]. [cited 2020 Aug 31]. Available from: https://regroup-production.s3.amazonaws.com/documents/ReviewReference/149748231/Thames_2018_Incorporating%20Technology%20to%20Decrease%20Heart%20Failure%20Readmission%20Rates.pdf?AWSAccessKeyId=AKIAJBZQODCMKJA4H7DA&Expires=1598876751&Signature=sdrEPKJYpm2vl5KDYIm%2Bef1%2FY%2F8%3D

337. Tonet E, Maietti E, Chiaranda G, Vitali F, Serenelli M, Bugani G, et al. Physical activity intervention for elderly patients with reduced physical performance after acute coronary

© 2021 Singh K et al. *JAMA Network Open*.

syndrome (HULK study): rationale and design of a randomized clinical trial. BMC Cardiovasc Disord. 2018 Dec;18(1):98.

338. Wu W-C, Parent M, Dev S, Hearns R, Taveira TH, Cohen L, et al. Group medical visits after heart failure hospitalization: Study protocol for a randomized-controlled trial. Contemporary Clinical Trials. 2018 Aug;71:140–5.

339. Zhang P, Xing F-M, Li C-Z, Wang F-L, Zhang X-L. Effects of a nurse-led transitional care programme on readmission, self-efficacy to implement health-promoting behaviours, functional status and life quality among Chinese patients with coronary artery disease: A randomised controlled trial. J Clin Nurs. 2018 Mar;27(5–6):969–79.

340. Kirkevold M, Kildal Bragstad L, Bronken BA, Kvigne K, Martinsen R, Gabrielsen Hjelle E, et al. Promoting psychosocial well-being following stroke: study protocol for a randomized, controlled trial. BMC Psychol. 2018 Dec;6(1):12.

341. Van Spall HGC, Lee SF, Xie F, Ko DT, Thabane L, Ibrahim Q, et al. Knowledge to action: Rationale and design of the Patient-Centered Care Transitions in Heart Failure (PACT-HF) stepped wedge cluster randomized trial. American Heart Journal. 2018 May;199:75–82.

342. Kaiser Permanente. An Evaluation of Clinical Pharmacist-led Intervention on Clinical Outcomes in Patients With Ischemic Stroke [Internet]. clinicaltrials.gov; 2018 Dec [cited 2020 Aug 25]. Report No.: NCT01876667. Available from: https://clinicaltrials.gov/ct2/show/NCT01876667

343. World Stroke Congress Abstracts, 2018, 2018 [Internet]. [cited 2020 Aug 29]. Available from: https://journals.sagepub.com/doi/10.1177/1747493018789543

344. Dean SG, Poltawski L, Forster A, Taylor RS, Spencer A, James M, et al. Community-based rehabilitation training after stroke: results of a pilot randomised controlled trial (ReTrain) investigating acceptability and feasibility. BMJ Open. 2018 Feb;8(2):e018409.

345. Jyotishana K, Sharma K, Hote M. A pilot study to assess the effectiveness of cardiac rehabilitative teaching program on quality of life and physiological parameters among patients undergoing coronary artery bypass grafting in tertiary care hospital. J Clin Prev Cardiol. 2018;7(4):137.

346. Aronov D, Bubnova M, Iosseliani D, Orekhov A. Clinical Efficacy of a Medical Centre- and Home-based Cardiac Rehabilitation Program for Patients with Coronary Heart Disease After Coronary Bypass Graft Surgery. Archives of Medical Research. 2019 Apr;50(3):122–32.

347. Boden-Albala B, Goldmann E, Parikh NS, Carman H, Roberts ET, Lord AS, et al. Efficacy of a Discharge Educational Strategy vs Standard Discharge Care on Reduction of Vascular Risk in Patients With Stroke and Transient Ischemic Attack: The DESERVE Randomized Clinical Trial. JAMA Neurol. 2019 Jan 1;76(1):20.

348. Britto-2019-Effectiveness of home-based cardia.pdf [Internet]. [cited 2020 Aug 31]. Available from: https://regroup-production.s3.amazonaws.com/documents/ReviewReference/149749695/Britto-2019-
© 2021 Singh K et al. *JAMA Network Open*.

Effectiveness%20of%20home-based%20cardia.pdf?AWSAccessKeyId=AKIAJBZQODCMKJA4H7DA&Expires=1598879204&Signature=1b%2BNbRr1NxBCrd0i02P1kX1GrfM%3D

349. Carlson B, Austel Nadeau C, Glaser D, Fields W. Evaluation of the effectiveness of the healthy heart tracker on heart failure self-care. Patient Education and Counseling. 2019 Jul;102(7):1324–30.

350. Corones-Watkins KM, Theobald KA, White KM. Outcomes of a randomised pilot trial of a nurse-led clinic for patients after percutaneous coronary intervention. Australian Critical Care. 2019 Jul;32(4):285–92.

351. Cramer SC, Dodakian L, Le V, See J, Augsburger R, McKenzie A, et al. Efficacy of Home-Based Telerehabilitation vs In-Clinic Therapy for Adults After Stroke. JAMA Neurol. 2019 Sep;76(9):1079–87.

352. Cui X, Zhou X, Ma L, Sun T-W, Bishop L, Gardiner FW, et al. A nurse-led structured education program improves self-management skills and reduces hospital readmissions in patients with chronic heart failure: a randomized and controlled trial in China [Internet]. Vol. 19. 2019 [cited 2020 Aug 31]. Available from: https://www.rrh.org.au/journal/article/5270/

353. Dahl Stephanie. Abstract WP471: Increasing Stroke Education Retention and Quality of Life in the Outpatient Setting. Stroke. 50(Suppl_1):AWP471–AWP471.

354. Dorsch MP, Farris KB, Hummel SL, Koelling TM. A Patient-Centered Mobile Intervention to Promote Self-Management and Improve Patient Outcomes in Chronic Heart Failure: The ManageHF Trial. Journal of Cardiac Failure. 2019 Aug;25(8):S104.

355. Dunn SL, Robbins LB, Smith SW, Ranganathan R, DeVon HA, Collins EG, et al. Enhancing physical activity in cardiac patients who report hopelessness: Feasibility testing of an intervention. Health Education Journal. 2019 Mar;78(2):226–37.

356. Lang CC, Smith K, Wingham J, Eyre V, Greaves CJ, Warren FC, et al. A randomised controlled trial of a facilitated home-based rehabilitation intervention in patients with heart failure with preserved ejection fraction and their caregivers: the REACH-HFpEF Pilot Study. Open Access. :12.

357. Gong E, Gu W, Sun C, Turner EL, Zhou Y, Li Z, et al. System-integrated technology-enabled model of care to improve the health of stroke patients in rural China: protocol for SINEMA—a cluster-randomized controlled trial. American Heart Journal. 2019 Jan;207:27–39.

358. Gonzalez M, Sjölin I, Bäck M, Ögmundsdottir Michelsen H, Tanha T, Sandberg C, et al. Effect of a lifestyle-focused electronic patient support application for improving risk factor management, self-rated health, and prognosis in post-myocardial infarction patients: study protocol for a multi-center randomized controlled trial. Trials. 2019 Dec;20(1):76.

359. Irewall A-L, Ögren J, Bergström L, Laurell K, Söderström L, Mooe T. Nurse-led, telephone-based secondary preventive follow-up benefits stroke/TIA patients with low education: a randomized controlled trial sub-study. Trials. 2019 Dec;20(1):52.

360. Jayawardena R, Sooriyaarachchi P, Punchihewa P, Lokunarangoda N, Pathirana AK. Effects of “plate model” as a part of dietary intervention for rehabilitation following myocardial infarction: a randomized controlled trial. Cardiovasc Diagn Ther. 2019 Apr;9(2):179–88.

361. Kim KA, Hwang SY. Effects of a Daily Life-Based Physical Activity Enhancement Program for Middle-Aged Women at Risk for Cardiovascular Disease. J Korean Acad Nurs. 2019;49(2):113.

362. McCreight MS, Lambert-Kerzner A, O’Donnell CI, Grunwald GK, Hebert P, Gillette M, et al. Improving anti-platelet therapy adherence in the Veterans Health Administration: A randomized multi-site hybrid effectiveness-implementation study protocol. Contemporary Clinical Trials. 2019 Feb;77:104–10.

363. Nordin NAM, Aziz NA, Sulong S, Aljunid SM. Effectiveness of home-based carer-assisted in comparison to hospital-based therapist-delivered therapy for people with stroke: A randomised controlled trial. NRE. 2019 Sep 25;45(1):87–97.

364. Oscalices MIL, Okuno MFP. Discharge guidance and telephone follow-up in the therapeutic adherence of heart failure: randomized clinical trial. :9.

365. Abstracts Programme - 2019 - European Journal of Heart Failure - Wiley Online Library [Internet]. [cited 2020 Aug 31]. Available from: https://onlinelibrary.wiley.com/doi/abs/10.1002/ejhf.1488

366. Qian C, Zhong D, Shen Y, Du Q. Evaluation of clinical efficacy of transitional care mode for patients with strokes. :8.

367. Redfern J, Hyun K, Singleton A, Hafiz N, Raeside R, Spencer L, et al. ITM support for patients with chronic respiratory and cardiovascular diseases: a protocol for a randomised controlled trial. BMJ Open. 2019 Mar;9(3):e023863.

368. Rodgers H, Shaw L, Bhattarai N, Cant R, Drummond A, Ford GA, et al. 137A trial to evaluate an eXTended RehAbilitation service for Stroke patients (EXTRAS): main patient results. Age and Ageing. 2019 Feb 1;48(Supplement_1):i40–i40.

369. Ruiz-Bustillo S, Ivern C, Badosa N, Farre N, Marco E, Bruguera J, et al. Efficacy of a nurse-led lipid-lowering secondary prevention intervention in patients hospitalized for ischemic heart disease: A pilot randomized controlled trial. European Journal of Cardiovascular Nursing. 2019 Jun;18(5):366–74.

370. Shao J-H, Chen S-H. Randomized control trial of a self-management intervention for heart failure older adults in Northern Taiwan. Collegian. 2019 Apr;26(2):288–94.

371. E-Poster Viewing – Miscellaneous. European Stroke Journal. 2019 May;4(1_suppl):790–821.

372. Tsuyuki RT, Lockwood EE, Shibata MC, Simpson SH, Tweden KL, Gutierrez R, et al. A Randomized Trial of Video-based Education in Patients With Heart Failure: The Congestive Heart Failure Outreach Program of Education (COPE). CJC Open. 2019 Mar;1(2):62–8.

373. Late Breaking Abstracts. European Stroke Journal. 2019 May;4(1_suppl):779–89.

374. Effect of Continuous Nursing Intervention on Rehabilitation after Coronary Artery Bypass Grafting in Elderly Patients with Coronary Heart Disease. Acta Medica Mediterranea [Internet]. 2019 [cited 2020 Aug 31];(1s). Available from: http://doi.org/10.19193/0393-6384_2019_1s_74

375. VA Office of Research and Development. Stroke Self-Management: Effect on Function and Stroke Quality of Life [Internet]. clinicaltrials.gov; 2019 Aug [cited 2020 Aug 25]. Report No.: NCT01507688. Available from: https://clinicaltrials.gov/ct2/show/NCT01507688

376. Cuesta-Vargas DAI. Clinical Effect Size of an Educational Intervention in the Home and Compliance With a Smartphone-based Reminder on People Who Suffer From Stroke: a Protocol Study. [Internet]. clinicaltrials.gov; 2019 Sep [cited 2020 Aug 27]. Report No.: NCT01980641. Available from: https://clinicaltrials.gov/ct2/show/NCT01980641

377. Kennelty K. MEDication Focused Outpatient Care for Underutilization of Secondary Prevention [Internet]. clinicaltrials.gov; 2019 Feb [cited 2020 Aug 27]. Report No.: NCT02215408. Available from: https://clinicaltrials.gov/ct2/show/NCT02215408

378. Post-discharge short message service improves short-term clinical outcome and self-care behaviour in chronic heart failure - Chen - 2019 - ESC Heart Failure - Wiley Online Library [Internet]. [cited 2020 Aug 31]. Available from: https://onlinelibrary.wiley.com/doi/full/10.1002/ehf2.12380

379. Tang W. Virtual Visits in Heart Failure Care Transitions [Internet]. clinicaltrials.gov; 2019 Sep [cited 2020 Aug 30]. Report No.: NCT03675828. Available from: https://clinicaltrials.gov/ct2/show/NCT03675828

380. Spaulding Erin M., Marvel Francoise A., Lee Matthias A., Yang William E., Demo Ryan, Wang Jane, et al. Corrie Health Digital Platform for Self-Management in Secondary Prevention After Acute Myocardial Infarction. Circulation: Cardiovascular Quality and Outcomes. 2019 May 1;12(5):e005509.

381. Wolf A, Vella R, Fors A. The impact of person-centred care on patients' care experiences in relation to educational level after acute coronary syndrome: secondary outcome analysis of a randomised controlled trial. European Journal of Cardiovascular Nursing. 2019 Apr;18(4):299–308.

382. Wonggom P, Nolan P, Clark RA, Barry T, Burdeniuk C, Nesbitt K, et al. Effectiveness of an avatar educational application for improving heart failure patients' knowledge and self-care behaviors: A pragmatic randomized controlled trial. J Adv Nurs. 2020 Sep;76(9):2401–15.

383. ISRCTN - ISRCTN15643456: Biopsychosocial Intervention for Stroke Carers [Internet]. [cited 2020 Aug 31]. Available from: http://www.isrctn.com/ISRCTN15643456

384. Passaglia L, Nascimento BR, Brant LC, Ribeiro AL. Impact of Text Messages in a Middle-Income Country to Promote Secondary Prevention After Acute Coronary Syndrome (impacs): A Randomized Trial. J Am Coll Cardiol. 2020 Mar 24;75(11 Supplement 1):2003.

© 2021 Singh K et al. *JAMA Network Open*.

385. Evaluate the Effectiveness of Self-care Multifaceted Strategy in Heart Failure Patients - Full Text View - ClinicalTrials.gov [Internet]. [cited 2020 Aug 31]. Available from: https://clinicaltrials.gov/ct2/show/NCT04062461

386. Coordinated, Collaborative, Comprehensive, Family-based, Integrated, Technology-enabled Stroke Care - Full Text View - ClinicalTrials.gov [Internet]. [cited 2020 Aug 31]. Available from: https://clinicaltrials.gov/ct2/show/NCT04000971

387. Tomsk National Research Medical Center of the Russian Academy of Sciences. Effect of Digital Technologies on Risk Factor Modification in Patients After Percutaneous [Internet]. clinicaltrials.gov; 2020 Jul [cited 2020 Aug 30]. Report No.: nct03871907. Available from: https://clinicaltrials.gov/ct2/show/nct03871907

388. ICTRP Search Portal [Internet]. [cited 2020 Aug 27]. Available from: https://apps.who.int/trialsearch/Trial2.aspx?TrialID=ACTRN12610000288022

389. Damush et al. Implementation of a stroke self-management program [Internet]. [cited 2020 Aug 27]. Available from: https://www.ncbi.nlm.nih.gov/pmc/articles/PMC3717676/

390. Depression Outpatient Cardiology Screening Study - Full Text View - ClinicalTrials.gov [Internet]. [cited 2020 Aug 27]. Available from: https://clinicaltrials.gov/ct2/show/NCT01552889

391. Trial of a Secondary Stroke Prevention Program - Full Text View - ClinicalTrials.gov [Internet]. [cited 2020 Aug 27]. Available from: https://clinicaltrials.gov/ct2/show/NCT01071408

392. ICTRP Search Portal [Internet]. [cited 2020 Aug 27]. Available from: https://apps.who.int/trialsearch/Trial2.aspx?TrialID=ACTRN12612001287820

393. ICTRP Search Portal [Internet]. [cited 2020 Aug 27]. Available from: https://apps.who.int/trialsearch/Trial2.aspx?TrialID=IRCT201107041464N4

394. ICTRP Search Portal [Internet]. [cited 2020 Aug 27]. Available from: https://apps.who.int/trialsearch/Trial2.aspx?TrialID=ISRCTN10823032

395. Clinical Decision Support for Medication Management and Adherence - Full Text View - ClinicalTrials.gov [Internet]. [cited 2020 Aug 27]. Available from: https://clinicaltrials.gov/ct2/show/NCT00979225

396. ICTRP Search Portal [Internet]. [cited 2020 Aug 27]. Available from: https://apps.who.int/trialsearch/Trial2.aspx?TrialID=IRCT201202128978N1

397. Telehealth Self Management for CHF - Full Text View - ClinicalTrials.gov [Internet]. [cited 2020 Aug 27]. Available from: https://clinicaltrials.gov/ct2/show/NCT01812512

398. Home visit improves knowledge, self-care and adhesion in heart failure: randomized Clinical Trial HELEN-I [Internet]. [cited 2020 Aug 28]. Available from: https://www.scielo.br/scielo.php?script=sci_arttext&pid=S0104-11692013000700004&lng=en&tlng=en

© 2021 Singh K et al. *JAMA Network Open*.

399. Promoting Adherence to Improve Effectiveness of Cardiovascular Disease Therapies - Full Text View - ClinicalTrials.gov [Internet]. [cited 2020 Aug 28]. Available from: https://clinicaltrials.gov/ct2/show/NCT01251757

400. Iran Jahanbin. ICTRP Search Portal [Internet]. [cited 2020 Sep 1]. Available from: https://apps.who.int/trialsearch/Trial2.aspx?TrialID=IRCT201306297531N2

401. Abstract 16922: Feasibility of Family Sodium Watcher Program to Improve Adherence to Low Sodium Diet in Patients with Heart Failure and Caregivers | Circulation [Internet]. [cited 2020 Aug 28]. Available from: https://www.ahajournals.org/doi/10.1161/circ.130.suppl_2.16922

402. The effect of integrated nursing program on coping strategies and quality of life in patients with heart failure | Cochrane Library [Internet]. [cited 2020 Aug 28]. Available from: https://www.cochranelibrary.com/central/doi/10.1002/central/CN-01063696/full?cookiesEnabled

403. Improving Care of Patients With Heart Failure - Full Text View - ClinicalTrials.gov [Internet]. [cited 2020 Aug 28]. Available from: https://clinicaltrials.gov/ct2/show/NCT01461681

404. Reza Shekarriz. ICTRP Search Portal [Internet]. [cited 2020 Sep 1]. Available from: https://apps.who.int/trialsearch/Trial2.aspx?TrialID=IRCT2014051717727N1

405. Innovation in stroke education in the TIA and minor stroke (TAMS) unit | Request PDF [Internet]. ResearchGate. [cited 2020 Aug 28]. Available from: https://www.researchgate.net/publication/296089242_Innovation_in_stroke_education_in_the_TIA_and_minor_stroke_TAMS_unit

406. Optimising Congestive Heart Failure Outpatient Clinic Project - Full Text View - ClinicalTrials.gov [Internet]. [cited 2020 Aug 28]. Available from: https://clinicaltrials.gov/ct2/show/NCT01671995

407. ANZCTR - Registration [Internet]. [cited 2020 Aug 28]. Available from: https://www.anzctr.org.au/Trial/Registration/TrialReview.aspx?id=368254

408. ICTRP Search Portal [Internet]. [cited 2020 Aug 28]. Available from: https://apps.who.int/trialsearch/Trial2.aspx?TrialID=IRCT2015032021521N1

409. Medication Adherence Telemonitoring to Reduce Heart Failure Readmissions - Full Text View - ClinicalTrials.gov [Internet]. [cited 2020 Aug 28]. Available from: https://clinicaltrials.gov/ct2/show/NCT02378571

410. The Use of Texting Messaging to Improve the Hospital-to-community Transition Period in Cardiovascular Disease Patients - Full Text View - ClinicalTrials.gov [Internet]. [cited 2020 Aug 28]. Available from: https://clinicaltrials.gov/ct2/show/NCT02336919

411. Short Message Service (SMS) Reminders for Stroke Secondary Preventative Medications - Full Text View - ClinicalTrials.gov [Internet]. [cited 2020 Aug 28]. Available from: https://clinicaltrials.gov/ct2/show/NCT02336412

© 2021 Singh K et al. *JAMA Network Open*.

412. Interventions to Support Long-Term Adherence aNd Decrease Cardiovascular Events Post-Myocardial Infarction - Full Text View - ClinicalTrials.gov [Internet]. [cited 2020 Aug 28]. Available from: https://clinicaltrials.gov/ct2/show/NCT02382731

413. Effects of Training Caregivers on the Outcomes of Stroke Survivors and Caregivers in Zimbabwe - Full Text View - ClinicalTrials.gov [Internet]. [cited 2020 Aug 28]. Available from: https://clinicaltrials.gov/ct2/show/NCT02569099

414. ALLiance for sEcondary PREvention After an Episode of Acute Coronary Syndrome (ALLEPRE) - Full Text View - ClinicalTrials.gov [Internet]. [cited 2020 Aug 28]. Available from: https://clinicaltrials.gov/ct2/show/NCT02522182

415. Effects of a Community-based Group Rehabilitation Program for Dynamic Balance and Mobility Post Stroke - Full Text View - ClinicalTrials.gov [Internet]. [cited 2020 Aug 28]. Available from: https://clinicaltrials.gov/ct2/show/NCT01818271

416. The impact of clinical pharmacist support on patients receiving multi-drug therapy for coronary heart disease in China: a long-term follow-up study | European Journal of Hospital Pharmacy [Internet]. [cited 2020 Aug 28]. Available from: https://ejhp.bmj.com/content/22/6/323

417. VA Office of Research and Development. Group Medical Visits in Heart Failure for Post-Hospitalization Follow-Up [Internet]. clinicaltrials.gov; 2020 Apr [cited 2020 Aug 30]. Report No.: NCT02481921. Available from: https://clinicaltrials.gov/ct2/show/NCT02481921

418. Saiede Fatemeh Gheiasi. ICTRP Search Portal [Internet]. [cited 2020 Sep 1]. Available from: https://apps.who.int/trialsearch/Trial2.aspx?TrialID=IRCT2015020320940N1

419. Collaborative goal-setting and action plans in therapeutic patient education for stroke patients in rehabilitation phase [Internet]. [cited 2020 Aug 29]. Available from: https://iris.unimore.it/handle/11380/1117582

420. NYU Langone Health. Empowering Individuals Post-Cardiopulmonary Outpatient Rehabilitation to Continue to Live a Heart Healthy Lifestyle: Utilizing Mobile Health Technology [Internet]. clinicaltrials.gov; 2020 May [cited 2020 Aug 27]. Report No.: NCT02671669. Available from: https://clinicaltrials.gov/ct2/show/NCT02671669

421. Webster J, Osborne S, Rickard CM, New K. Clinically-indicated replacement versus routine replacement of peripheral venous catheters. Cochrane Database of Systematic Reviews [Internet]. 2015 [cited 2020 Aug 29];(8). Available from: https://www.cochranelibrary.com/cdsr/doi/10.1002/14651858.CD007798.pub4/full

422. Chen Y. Effectiveness of a multidisciplinary disease management program on outcomes in patients with heart failure in China: a randomized controlled single center study | Cochrane Library [Internet]. [cited 2020 Aug 29]. Available from: https://www.cochranelibrary.com/central/doi/10.1002/central/CN-01443094/full

423. Tayebe. ICTRP Search Portal [Internet]. [cited 2020 Aug 29]. Available from: https://apps.who.int/trialsearch/Trial2.aspx?TrialID=IRCT2016052628089N1

© 2021 Singh K et al. *JAMA Network Open*.

424. Living Well With Stroke - Full Text View - ClinicalTrials.gov [Internet]. [cited 2020 Aug 29]. Available from: https://clinicaltrials.gov/ct2/show/NCT01133106

425. Impact of a Therapeutic Education Program of Patient on the Evolution of the Cardiovascular Chronic Disease - Full Text View - ClinicalTrials.gov [Internet]. [cited 2020 Aug 29]. Available from: https://clinicaltrials.gov/ct2/show/NCT02819791

426. The Congestive Heart Failure Adherence Redesign Trial - Full Text View - ClinicalTrials.gov [Internet]. [cited 2020 Aug 29]. Available from: https://clinicaltrials.gov/ct2/show/NCT01698242

427. Abstract 15002: Hospital Strategies to Address Non-Adherence to Guideline-Recommended Dual Antiplatelet Therapy After Myocardial Infarction: Findings From the Affordability and Real-World Antiplatelet Treatment Effectiveness After Myocardial Infarction Study (ARTEMIS) | Circulation [Internet]. [cited 2020 Aug 29]. Available from: https://www.ahajournals.org/doi/10.1161/circ.134.suppl_1.15002

428. SPRITE - A Feasibility and Pilot Study - Full Text View - ClinicalTrials.gov [Internet]. [cited 2020 Aug 31]. Available from: https://clinicaltrials.gov/ct2/show/NCT02712385

429. Abstract 164: The Michigan Stroke Transitions Trial: Assessment of Unmet Needs and Case Management Activities from an In-home Patient-Centered Social Work Case Management Program | Stroke [Internet]. [cited 2020 Aug 31]. Available from: https://www.ahajournals.org/doi/10.1161/str.48.suppl_1.164

430. Tuulikki Sjögren. ICTRP Search Portal [Internet]. [cited 2020 Aug 31]. Available from: https://apps.who.int/trialsearch/Trial2.aspx?TrialID=ISRCTN61225589

431. Ashok Kumar. ICTRP Search Portal [Internet]. [cited 2020 Aug 31]. Available from: https://apps.who.int/trialsearch/Trial2.aspx?TrialID=CTRI/2017/08/009267

432. Boyoung Hwang. ICTRP Search Portal [Internet]. [cited 2020 Aug 31]. Available from: https://apps.who.int/trialsearch/Trial2.aspx?TrialID=KCT0002400

433. Aliya Hisam. ICTRP Search Portal [Internet]. [cited 2020 Aug 31]. Available from: https://apps.who.int/trialsearch/Trial2.aspx?TrialID=ACTRN12617000832370

434. Tahereh Najafi Ghezeljeh. ICTRP Search Portal [Internet]. [cited 2020 Aug 31]. Available from: https://apps.who.int/trialsearch/Trial2.aspx?TrialID=IRCT2016071328920N1

435. AACVPR 32nd Annual Meeting Scientific Abstract Presentations : Journal of Cardiopulmonary Rehabilitation and Prevention [Internet]. [cited 2020 Aug 31]. Available from: https://journals.lww.com/jcrjournal/Citation/2017/09000/AACVPR_32nd_Annual_Meeting_Scientific_Abstract.12.aspx

436. Stroke Patients' Outpatient Rehabilitation Therapy (SPORT) - Full Text View - ClinicalTrials.gov [Internet]. [cited 2020 Aug 31]. Available from: https://clinicaltrials.gov/ct2/show/NCT03165630

© 2021 Singh K et al. *JAMA Network Open*.

437. 代(武汉市中西医结合医院，中国)智烈. Open Journal Systems. [cited 2020 Sep 1]; Available from: https://ojs.s-p.sg/index.php/yzlcyxzz/article/view/4410

438. 病人照顾者综合康复护理培训联合延续护理对缺血性脑卒中病人居家生活质量及日常生活能力的影响-【维普官方网站】-www.cqvip.com-维普网 [Internet]. [cited 2020 Sep 1]. Available from: http://www.cqvip.com/qk/87333x/201707/672564599.html

439. A Randomized Trial of Heart.pdf [Internet]. [cited 2020 Aug 31]. Available from: https://regroup-production.s3.amazonaws.com/documents/ReviewReference/149746040/A%20Randomized%20Trial%20of%20Heart.pdf?AWSAccessKeyId=AKIAJBZQODCMKJA4H7DA&Expires=1598868929&Signature=wCsRYe%2FUXe5KEds%2BMkOevuaqHQE%3D

440. (PDF) Home-based physical activity incentive and education program in subacute phase of stroke recovery (Ticaa’dom): Study protocol for a randomized controlled trial [Internet]. [cited 2020 Aug 31]. Available from: https://www.researchgate.net/publication/322712026_Home-based_physical_activity_incentive_and_education_program_in_subacute_phase_of_stroke_recovery_Ticaa'dom_Study_protocol_for_a_randomized_controlled_trial

441. Effectiveness of a multidisciplinary disease management program on outcomes in patients with heart failure in China: A randomized controlled single center study - Heart & Lung: The Journal of Cardiopulmonary and Acute Care [Internet]. [cited 2020 Aug 31]. Available from: https://www.heartandlung.org/article/S0147-9563(17)30095-X/fulltext

442. Selfcare Management Intervention in Heart Failure - Full Text View - ClinicalTrials.gov [Internet]. [cited 2020 Aug 31]. Available from: https://clinicaltrials.gov/ct2/show/NCT03484286

443. Cardiac Care Solution for Coronary Disease Follow up - Full Text View - ClinicalTrials.gov [Internet]. [cited 2020 Aug 31]. Available from: https://clinicaltrials.gov/ct2/show/NCT03565978

444. Interventions to Support Long-Term Adherence aNd Decrease Cardiovascular Events Post-Myocardial Infarction - Full Text View - ClinicalTrials.gov [Internet]. [cited 2020 Sep 2]. Available from: https://clinicaltrials.gov/ct2/show/NCT02382731

445. Kitsiou S. iCardia4HF: A Patient-centered Mobile Health Intervention to Promote Self-care and Improve Patient Outcomes in Chronic Heart Failure [Internet]. clinicaltrials.gov; 2020 Apr [cited 2020 Aug 27]. Report No.: NCT03642275. Available from: https://clinicaltrials.gov/ct2/show/NCT03642275

446. Muyiwa-Ojo_2018_Thirty-Day Readmissions Reduction Using Teach-Back Telephone Education.pdf [Internet]. [cited 2020 Aug 31]. Available from: https://regroup-production.s3.amazonaws.com/documents/ReviewReference/149744808/Muyiwa-Ojo_2018_Thirty-Day%20Readmissions%20Reduction%20Using%20Teach-Back%20Telephone%20Education.pdf?AWSAccessKeyId=AKIAJBZQODCMKJA4H7DA&Expires=1598875736&Signature=n0GYbeQk7z9qrBiGqE2zJ2RL%2FHs%3D

© 2021 Singh K et al. *JAMA Network Open*.

447. Saman Parvar. ICTRP Search Portal [Internet]. [cited 2020 Aug 31]. Available from: https://apps.who.int/trialsearch/trial2.Aspx?Trialid=actrn12618000250235

448. Intervention to Enable Stroke Survivors in Los Angeles County Hospitals to “Stay Within the Guidelines” - Full Text View - ClinicalTrials.gov [Internet]. [cited 2020 Aug 31]. Available from: https://clinicaltrials.gov/ct2/show/NCT00861081

449. Dominique Cadilhac. ICTRP Search Portal [Internet]. [cited 2020 Sep 1]. Available from: https://apps.who.int/trialsearch/Trial2.aspx?TrialID=ACTRN12618001468213

450. Lyvia da Silva. ICTRP Search Portal [Internet]. [cited 2020 Sep 1]. Available from: https://apps.who.int/trialsearch/Trial2.aspx?TrialID=RBR-9c3ssc

451. Reza Haji Ali. ICTRP Search Portal [Internet]. [cited 2020 Sep 1]. Available from: https://apps.who.int/trialsearch/Trial2.aspx?TrialID=IRCT20171223038022N1

452. Rohit Bhatia. ICTRP Search Portal [Internet]. [cited 2020 Sep 1]. Available from: https://apps.who.int/trialsearch/Trial2.aspx?TrialID=CTRI/2018/11/016312

453. “Quality improvement: improving primary care follow-up for stroke/TIA p” by Muhammad S. Khan, Gamaleldin Osman et al. [Internet]. [cited 2020 Sep 1]. Available from: https://scholarlycommons.henryford.com/neurology_mtgabstracts/20/

454. Journal of General Internal Medicine [Internet]. Springer. [cited 2020 Aug 31]. Available from: https://www.springer.com/journal/11606

455. Jain YS. Drug Utilization and Quality of Life Study in Coronary Artery Disease Patients in a North Indian Tertiary Care Center [Internet]. clinicaltrials.gov; 2020 Feb [cited 2020 Aug 30]. Report No.: NCT04271566. Available from: https://clinicaltrials.gov/ct2/show/NCT04271566

456. Shirin Madadkar. ICTRP Search Portal [Internet]. [cited 2020 Sep 1]. Available from: https://apps.who.int/trialsearch/Trial2.aspx?TrialID=IRCT20181122041720N2

© 2021 Singh K et al. *JAMA Network Open*.

eFigure 1. Number and Types of Studies by Publication Year

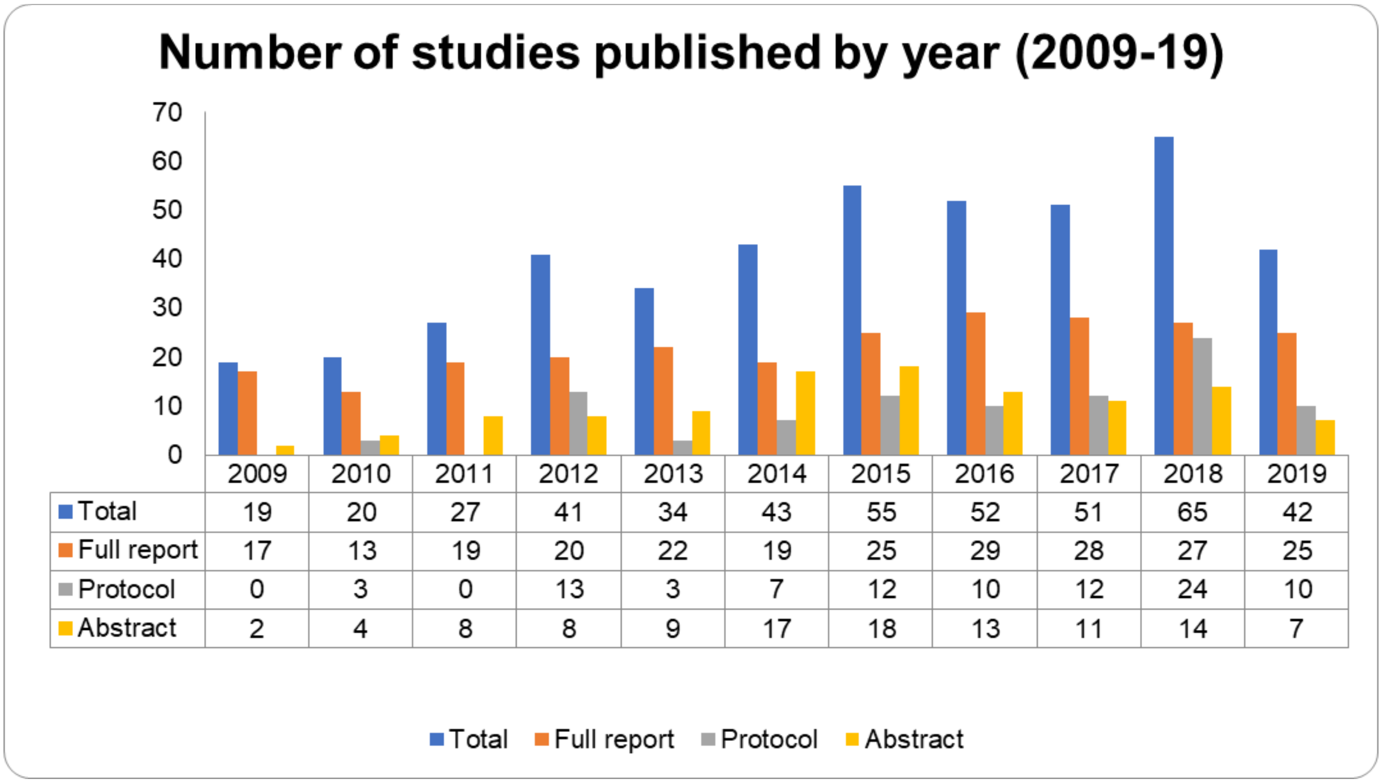

	2009	2010	2011	2012	2013	2014	2015	2016	2017	2018	2019
Total	19	20	27	41	34	43	55	52	51	65	42
Full report	17	13	19	20	22	19	25	29	28	27	25
Protocol	0	3	0	13	3	7	12	10	12	24	10
Abstract	2	4	8	8	9	17	18	13	11	14	7

© 2021 Singh K et al. *JAMA Network Open*.

eFigure 2. Number of Studies by Location

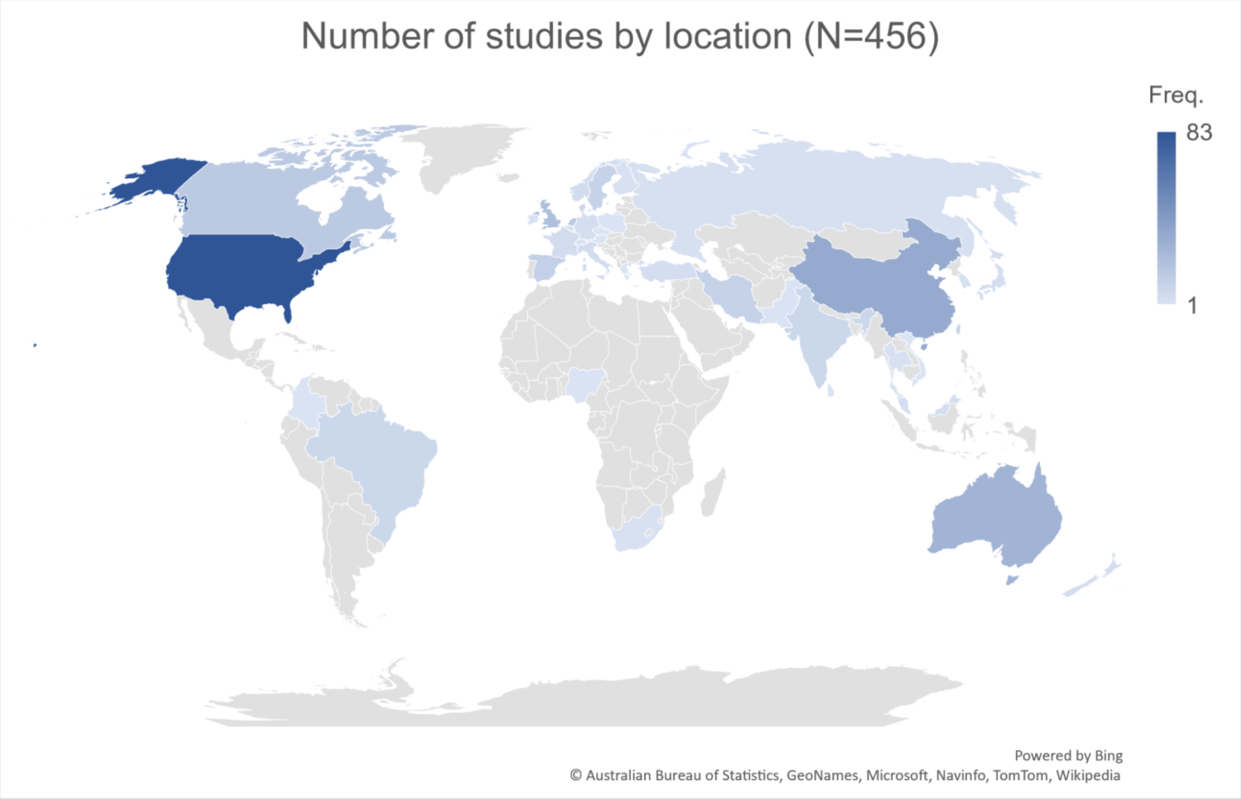

© 2021 Singh K et al. *JAMA Network Open*.

eFigure 3. Comparative Description of Intervention Type and Implementation Strategies

**Implementation strategies**	Patient Support	ICT-Health	Community Support	High intensity training	Supervision	Group problem solving	Printed information	Strengthening infrastructure	Financial incentives	Other mgmt techniques
Train and educate stakeholders	146	34	13	12	8	6	2		1	1
Engage consumers	64	11	1		2		2	2		
Provide interactive assistance	91	17	2	1	2	1	1			
Change infrastructure	8	8			1			1		
Adapt and tailor to the context	6	2			1					
Use evaluative and iterative strategies	1	3	3		1					
Utilize financial strategies	1							1	2	
Develop stakeholder interrelationship	2			1						
Support clinicians		2								
**Chronic Care Model elements**										
Combination	118	31	10	8	7	2	2	1	1	1
Decision support	86	11	1		2	2	1	1		
Delivery system design	57	15	3	2	4		1	2	1	
Self-management support	34	10	3	4	1	3	1		1	
Clinical information systems	15	10								
Community resources	1		1							
Organizational support		1		1						

Abbreviations: HCPPR=Health care provider performance review, ICT=Information communication and technology, Other mgmt techniques=management techniques

>25 studies
<25 to 5 studies
<5 to 1 studies
0 studies

© 2021 Singh K et al. *JAMA Network Open*.

eFigure 4. Intervention Type and Patient Population

**Intervention type**	**Heart Failure**	**Stroke**	**Post-MI**	**Stable CAD**	**CVD**	**CVD+DM**	**PAD**
Patient Support	121	83	43	32	27	4	1
ICT-Health	34	17	11	6	8	1	1
Community Support	4	9	2	2	1		
Supervision	5	2	3	3	2		
High intensity training	4	5	2	1	2		
Group problem solving	2	3	1	1			
Printed information		2	1				
Strengthening infrastructure	2	2		1			
Financial incentives	1	2	1				
Other management technique		1					

*MI=myocardial infarction, CAD=Coronary artery disease, CVD=cardiovascular disease, DM=diabetes mellitus, PAD=Peripheral arterial disease; ICT-Health=information communication and technology

>25 studies
<25 to 5 studies
<5 to 1 studies
0 studies

© 2021 Singh K et al. *JAMA Network Open*.

eFigure 5. Intervention Type and Clinical Setting

**Intervention**	**Tertiary care Hospital**	**Hospital + Home care**	**Secondary care Hospital**	**Primary care hospital**	**Community Level hospital**
Patient Support	146	92	6	10	4
ICT-Health	43	17	0	5	3
Community Support	2	8	1	4	1
Supervision	9	4			
High intensity training	11	2			
Group problem solving	3	1	1		
Printed information	2	1			
Strengthening infrastructure	4	3			
Financial incentives	1	1			
Other management technique		1			

*ICT=information communication and technology

>25 studies
<25 to 5 studies
<5 to 1 studies
0 studies

© 2021 Singh K et al. *JAMA Network Open*.

eFigure 6. Intervention Type and Study Location

Intervention Type	ECA	NA	EAP	MENA	LAC	SA	SSA
Patient Support	67	58	61	11	7	7	2
ICT-Health	20	26	16	1	2		
Community Support	4	3	7			2	
Supervision	9	3	1		1		
High intensity training	4	4	3			1	
Group problem solving	4		2				
Printed information	1	1	1				
Strengthening infrastructure		2	1				1
Financial incentives	1	0	1				
Other management technique							1

*ICT=Information communication and technology

N=Total number of studies; *146 studies did not provide details on study location.

ECA=Europe and Central Asia, NA=North America, EAP=East Asia and Pacific; MENA=Middle East and North Africa, LAC=Latin American and Caribbean, SA=South Asia, SSA=Sub-Saharan Africa

>25 studies
<25 to 5 studies
<5 to 1 studies
0 studies

© 2021 Singh K et al. *JAMA Network Open*.

eFigure 7. Detailed Summary of Overall Study Results Using Matrix Framework for the Cardiovascular Quality Improvement Strategies and Primary Outcomes Evaluated in the Included Studies (N = 456)

QI Strategies/Outcomes	Deaths				MACE				QoL				CE				BP				Lipids			
	+	0	-	NA	+	0	-	NA	+	0	-	NA	+	0	-	NA	+	0	-	NA	+	0	-	NA
Patient Support	6	7		1	6	2	1	4	19	10		8	5				10	6		4	3	6		2
IT & CT-Health	3	4							7	1		3		1			3	1		2	1			2
Community Support		1		1	1					1		1	1							1				
High intensity training	1								1	1								1						
Supervision								1		1		1	1											
Group problem solving									1			1												
Printed info																								
Strengthening infrastructure													1											
Financial incentives																				1				
Other management techniques									1															
**Intervention-Delivery mode**																								
Combination	3	6		1	3		1	1	8	4		4	2	2			7	2		5	2	4		2
Non-physician health worker	2	5		1	3	1		2	10	4		4	2				2	4		1		2		1
Phone calls	2				1	1			2	3		2					1			1				1
Technology (DS-EHR)	1								4	1		2	1				1			1	1			
Text messages																								

QI Strategies/Outcomes	Meds adh				Tob cess				Self-care				Exercise				Wt				Anx/Dep			
	+	0	-	NA	+	0	-	NA	+	0	-	NA	+	0	-	NA	+	0	-	NA	+	0	-	NA
Patient Support	20	7	1	7	5	6			17	7		4	8	1	1	5	4	3		1	13	7		5
IT & CT-Health	2	1		3	2				1	2		2	3	1		3					1	1		
Community Support								1						1		1								
High intensity training				1	1				1			1	2	3				2			1	1		
Supervision	2											1	2	1		2	1							
Group problem solving									3															
Printed info		1		1																				
Strengthening infrastructure																					1			
Financial incentives																1								
Other management techniques																								
**Intervention-Delivery mode**																								
Combination	8	2		5	4	5		1	12	5		1	4	4		3	2	3		1	5	3		2
Non-physician health worker	7	2	1	2	2				7	1		2	8	2	1	4	2	2			7	5		1
Phone calls	5				1				1			1	1	1							3	1		
Technology (DS-EHR)		2		2					1	2		1	1											
Text messages	3			1												2								

QI Strategies/Outcomes	Pt edu				Rx satisf.				Hosp re-adm				LOS				Feasibility				Others			
	+	0	-	NA	+	0	-	NA	+	0	-	NA	+	0	-	NA	+	0	-	NA	+	0	-	NA
Patient Support	12	2		2	3	1			21	10	2	7	1	5			2			1	40	24	2	19
IT & CT-Health		4							10	6		1	2			2				1	7	12		4
Community Support	1								2	1							3				1	2		1
High intensity training		1															1				3	5		
Supervision		1							2				1										1	
Group problem solving																					3	1		1
Printed info										1											2			
Strengthening infrastructure					1																	1		
Financial incentives										1														
Other management techniques																								
**Intervention-Delivery mode**																								
Combination	2	5			1				8	9		5		2			3				18	23		8
Non-physician health worker	7				3				14	5	1	1	2	2			3				17	16		7
Phone calls	1	2							4	1		1	1								5	4		1
Technology (DS-EHR)	1	1			1	1			4	4	1		1			2				1	6	4		3
Text messages	1											1								1				

*IT&CT=information, communication, and technology, DS-EHR=decision supported electronic health records, Printed info=printed information, Combination=combining more than one form of intervention delivery mode, e.g., non-physician health worker, phone calls, technology, or text-messages.

N=number of studies
Positive results (+)
Neutral results (0)
Negative results (-)
Results not available (NA)

© 2021 Singh K et al. *JAMA Network Open*.

eTable 1. Domains and Frameworks Used to Map Intervention Components, Implementation Strategies, and Context

**Intervention components (HCPPR)**	**Implementation strategies (ERIC study)**	**Context (Chronic care model)**
**Patient support**<br>Examples include patient health education via health worker or home visits.	**Engage consumers.** Examples include involve patients and family members in care delivery, use mass media.	**Organizational support.** Examples include develop agreements that support care coordination, provide incentive based on quality of care.
**Community support**<br>Examples include community health education or social marketing of health services.	**Use evaluative and iterative strategies.** Examples include conduct local need assessment, audit and provide feedback, implement tools for quality monitoring.	**Community resources.** Examples include form partnerships with community organizations to support and develop interventions.
**Information and communication technology for health**<br>This category includes mHealth and eHealth. Examples include computerized decision aids or text message reminders sent to care providers' or patients' phones.	**Change infrastructure.** Examples include change patient record systems, change physical structure or equipment or service sites	**Self-Management Support**. Examples include use of strategies that involve assessment, goal setting, problem solving and follow-up.
**Strengthening infrastructure**<br>Examples include provision of medicines or implementation of an improved data collection system.	**Adapt and tailor to context.** Examples include tailor strategies, use data experts.	**Delivery System Design.** Examples include provide clinical case management services, ensure regular follow-up by the care team.
**Health-care provider-directed financial incentives**<br>Examples include performance-based payments.	**Develop stakeholder interrelationships.**<br>Examples include use advisory boards and workgroups, inform local opinion leaders.	**Decision Support.** Examples include embed evidence-based guidelines into daily clinical practice, use proven education methods.
**Health system financing and other incentives**<br>Examples include social health insurance or reducing a consultation fee.	**Utilize financial strategies.** Examples include use payment schemes.	**Clinical Information Systems.** Examples include provide timely reminders to patients and providers.
**Regulation and governance**<br>Examples include accreditation or introducing standard drug quality requirements.	**Support clinicians**. Examples include facilitate relay of clinical data to providers, remind clinicians,	
**Group problem solving**<br>Examples include collaborative improvement or group problem solving with or without formal teams.	**Provide interactive assistance**. Examples include provide technical assistance, and clinical supervision.	

© 2021 Singh K et al. *JAMA Network Open*.

**Supervision**<br>Examples include improving routine supervision, benchmarking, or audit with feedback.	**Train and educate stakeholders.** Examples include conduct training and develop educational materials.	
**High-intensity training**<br>Training with a duration greater than 5 days (or ongoing training) and at least one interactive educational method.		
**Other management techniques**<br>These include techniques that do not include group problem solving and supervision, such as health-care provider self-assessment		
**Printed information** for patients and health-care providers. Examples include health information provided as a pamphlet.		

*HCPPR=Healthcare provider performance review; ERIC=Expert recommendations for Implementing change study

© 2021 Singh K et al. *JAMA Network Open*.

eTable 2. Descriptive Summary of Study Outcomes, Follow-up Duration, Sample Size, and Overall Results in the Included Studies (N = 456)

**Outcomes (primary)**	**N**	**%**
Deaths	36	7.9
Major adverse cardiovascular events	17	3.7
Medication adherence	51	11.2
Blood pressure	36	7.9
Blood cholesterol	21	4.6
Blood sugar	5	1.1
Physical activity/exercise	39	8.6
Weight / BMI (body mass index)	15	3.3
Smoking/tobacco cessation	17	3.7
Self-care behaviors	47	10.3
Anxiety/depression	32	7.0
Quality of life	71	15.6
Cost-effectiveness	11	2.4
Hospital re-admission rate	75	16.4
Length of hospital stay	13	2.9
Patient education	24	5.3
Treatment satisfaction	6	1.3
Waist circumference	4	0.9
Feasibility/Acceptability/Fidelity	8	1.8
**Follow-up duration, mean (in months)**	9.1	
Sample size, mean (range)	372	10 - 21752
Mean age (years, SD)	64.6	7.1
Female (%)	38.1	
**Economic data reported, yes (%)**	84	17.2
**Overall Results**		
Significant/Positive	227	49.7
Non-significant/Negative	76	16.6
Mixed	45	9.8
Not available	108	23.7

*SD=standard deviation.

© 2021 Singh K et al. *JAMA Network Open*.
